# Revisiting evidence for an attentional tunneling effect caused by scarcity

**DOI:** 10.1038/s41467-026-78074-y

**Published:** 2026-09-26

**Authors:** Fiona tho Pesch, Antonia F. Langenhoff, Mahesh Srinivasan

**Affiliations:** 1 Department of Psychology, University of Cologne, Cologne, Germany; 2 Département d’études cognitives, École normale supérieure – PSL, Paris, France; 3 Max Planck Institute for Research on Collective Goods, Bonn, Germany; 4 Department of Psychology, University of California Berkeley, California, USA; 5 Department of Psychology, Stanford University, California, USA

**Keywords:** Human behaviour, Decision making

## Abstract

People in poverty sometimes behave in ways that perpetuate their socioeconomic condition. By one influential account, these behaviors arise from psychological effects of scarcity on attention that create greater focus toward managing scarce resources, at the cost of attention to potentially-beneficial information outside of this ‘tunnel’. Although the tunneling hypothesis has been widely discussed in academia and beyond, its direct empirical support derives primarily from a landmark study by Shah et al. (2012) in which participants played games while their access to resources was experimentally manipulated, rendering participants either ‘rich’ or ‘poor’. Here, we present new analyses that contest the original interpretation of the data, showing that the behavioral differences between ‘rich’ and ‘poor’ participants are more parsimoniously explained by the game’s structure than by an attentional shift caused by the scarcity manipulation—a conclusion we substantiate through an additional experiment. We contextualize our findings within the literature on scarcity and attention, and highlight the need for greater clarity regarding the tunneling hypothesis itself.

## Abstract (web summary)

Scarcity is thought to create a tunneling effect, focusing attention on managing scarce resources to the neglect of other useful information. Here the authors reanalyse and report a conceptual replication of existing evidence for this effect, contesting the attribution of previously observed behavioral differences to an attentional shift.

## Introduction

People living in poverty sometimes exhibit behaviors that perpetuate their socioeconomic condition, including borrowing money at exorbitant interest rates^1^, and failing to enroll in subsidized insurance programs^2,3^. While previous accounts attributed these behaviors to personality traits^4^ or preference patterns^5^ of low-income individuals, a recent and influential account proposes that the experience of resource scarcity creates its own mindset, changing how anyone facing such conditions would think and make decisions^6–8^. The first key proposal of this scarcity theory is that the experience of scarcity induces a tax on cognitive capacities (i.e., a *bandwidth tax*): people are argued to experience more cognitive load when faced with scarcity^9–11^, and to consequently exhibit reduced cognitive control^12–14^. The second key proposal of scarcity theory–and the focus of the present paper–is the *tunneling effect*, according to which scarcity increases attention toward managing scarce resources themselves, resulting in some positive outcomes (*greater focus*; e.g., less susceptibility to framing effects)^15^, at the cost of attention to other potentially-important information, leading to some negative outcomes like the poverty-perpetuating behaviors mentioned in our opening sentence (*attentional neglect*; e.g., taking on payday loans due to not noticing their high interest rates).

While there is some evidence for the proposal of a bandwidth tax (for a review, see de Bruijn & Antonides)^6^, evidence for the positive and negative effects of tunneling primarily derives from a single, widely-cited paper published by Shah and colleagues in *Science* in 2012,^16^ which has been highly influential in academia and beyond^17–19^. Indeed, this work has shaped policy around international development and poverty alleviation^20^. Although several other studies have been cited as providing evidence for tunneling, these other studies have only offered partial support for the hypothesis, finding that individuals under scarcity exhibit greater focus (leading to positive outcomes including better memory for prices^7^, less susceptibility to framing effects^15^, and faster detection of words related to scarce resources)^12,21,22^, without demonstrating that this greater focus takes away from attention to other potentially-beneficial information (for more information, see the Discussion section). Thus, only Shah et al.^16^, have directly tested and substantiated the idea that scarcity makes people approach a task with a unique mindset, which leads them to focus their attention on managing their scarce resources, to the neglect of other information.

In Shah et al.^16^, participants played lab-based games while scarcity was experimentally manipulated. Here, we re-analyze the data from a key study in this paper–the ‘Angry Blueberries’ experiment (Study 2 of the original paper)–which, according to the authors, provides a cornerstone for their account by establishing the positive and negative effects of scarcity on attention within a single study^16,23^. We also reanalyze data from a large-scale replication of this experiment conducted by the same authors in 2019^24^. Our re-analyses contest the original interpretation of the data as providing evidence of an attentional tunneling effect, and we also present additional experimental evidence from a conceptual replication study supporting our alternative interpretation.

In the ‘Angry Blueberries’ game, participants shot blueberries, earning points for hitting targets (for specificities of the game, see Table 1). Scarcity was experimentally manipulated by randomly assigning some participants large and others small budgets of shots. ‘Poor’ participants (i.e., those endowed with fewer shots) spent significantly more time aiming at the targets and earned significantly more points per shot throughout the game than ‘rich’ participants (i.e., those endowed with more shots). This combination of longer aiming times and higher points-per-shot by the ‘poor’ ostensibly provides evidence for the positive consequences of tunneling, i.e., that participants under scarcity exhibit *greater focus* on tasks related to the scarce resource, which allows them to be more efficient at using that resource. Indeed, Shah and colleagues^16^ infer the attentional mechanism of focusing from observing these behavioral effects, claiming to find “scarcity’s focusing effect” (p. 684)^16^.

**Table 1:** Game specifications of ‘Angry Blueberries’

	‘Rich’	‘Poor’
**Aim of the game**	Earning points by shooting blueberries and hitting targets; each additional point increases the chance of winning a raffle	
**Targets**	7 targets on each level; targets disappear when hit, without replacement; targets are randomly spatially distributed on each level	
**Points**	Each cleared target equates to one point; 3 extra points are awarded if all targets for that level are cleared (i.e., a maximum of 10 points per level)	
**Experimental manipulation**	2 (‘poor’ vs. ‘rich’) × 2 (‘no borrow’ vs. ‘borrow’) design	
**Overall budget of shots**	150	30
**Budget of shots per level**	15	3
**Moving between levels**	Participants have the option to move to the next level after firing their first shot. In the no-borrow condition, participants automatically move to the next level when their budget of shots for that level is exhausted, or all targets are cleared	
**Overall number of levels**	Participants have to take at least one shot per level, but otherwise can move through as many levels as they want	
**Borrowing decision**	In the borrow condition, participants automatically “borrow” additional shots when they exceed their allotted shots for a given level. Borrowing only occurs after their budget is depleted, with a 100% interest rate applied: for each extra shot used on a given level, two shots are deducted from the overall budget. This process is automatic, with no option for participants to initiate borrowing manually.	
**Points-per-shot**	Measure for efficiency at shooting; if ‘rich’ and ‘poor’ would be equally efficient, they should show similar values on this measure	

A second key manipulation allowed a subset of ‘poor’ and ‘rich’ participants to borrow shots from future levels at a 100% interest rate (Table 1). Borrowing was only possible once participants exhausted their budget on a given level. ‘Poor’ participants borrowed a significantly larger proportion of their budget than ‘rich’ participants, resulting in worse overall performance. Thus, having the option to borrow from future rounds hurt the performance of the ‘poor’, but not the ‘rich’. Shah and colleagues^16^ take these data as providing evidence for the negative consequences of tunneling, and argue that scarcity causes individuals to neglect information outside their tunnel, like high interest rates, leading to suboptimal behaviors like over-borrowing: “…scarcity, of any kind, will create a tendency to borrow, with insufficient attention to whether the benefits outweigh the costs” (p. 683). Again, the original authors infer the mechanism of *attentional neglect* from observing borrowing behavior.

We note that the original claims put forward by Shah and colleagues^16^ have been widely accepted and integrated into subsequent, canonical works. For example, in their landmark book presenting the scarcity theory, Mullainathan and Shafir^7^ attribute the better performance of the ‘poor’ in the ‘Angry Blueberries’ study to heightened attentional focus: “…the poor did better: they were more accurate with their shots. This was not because of some magical improvement in visual acuity. The poor took more time on each shot. […] They aimed more carefully. They had fewer shots, so they were more judicious. The rich, on the other hand, just let the blueberries fly” (p. 25)^7^. Similarly, the tendency of ‘poor’ participants to overborrow has been attributed to attentional neglect. For example, in a recent review of the scarcity literature, de Bruijn and Antonides^6^ write that the ‘Angry Blueberries’ study “…suggests that people may pay too little attention to the future implications of borrowing as a result of facing financial scarcity” (p. 14). As these examples illustrate, Shah and colleagues’ claims–which we interrogate here–have figured prominently into subsequent presentations of the tunneling effect and its evidentiary basis.

In sum, the original authors inferred the attentional signatures of tunneling (i.e., greater focus and attentional neglect) from several behavioral outcomes in their ‘Angry Blueberries’ study. Our re-analysis reproduces these effects, both in the original dataset as well as in the large-scale replication from the same authors in 2019^24^. However, through additional analyses, we show that these behavioral outcomes do not provide evidence for the attentional shift described as tunneling (a critique that we show also applies to other studies within the original publication; see Supplementary Note 5)^16^. Instead, we show that the observed behavioral pattern can be explained more parsimoniously by specific elements of the game’s structure that constrained how ‘poor’ vs. ‘rich’ participants could play the game. We also present additional experimental evidence from a conceptual replication study supporting our alternative interpretation. Taken together, our findings undermine the extant empirical support for the tunneling hypothesis and raise questions about the clarity of the construct more generally, which we consider in the Discussion.

## Results

We conducted additional analyses that contest interpretations of the original data^16^ in terms of the tunneling hypothesis (see Table 2). These additional analyses undermine the assumption that the observed behavioral results (differences in performance and borrowing behavior) were caused by an ‘attentional shift’ in response to scarcity, which is the central claim of the original study. First, we show that the longer aiming times of the ‘poor’ did not actually translate into better performance, casting doubt on the original claim that these behavioral effects stemmed from *greater focus* (Table 2, Claims 1.1–1.3)^16^. Second, we show that the behavioral effects of overborrowing from this study cannot be attributed to *attentional neglect*, but were instead due to differences in base rate frequencies with which the ‘poor’ and the ‘rich’ faced the option to borrow (Table 2, Claims 2.1–3.1).

**Table 2:** Primary claims made by Shah et al.^16^ (Column A), original analyses supporting those claims reproduced in our re-analysis (Column B), and our new, additional analyses contesting the original claims (Column C)

A. Claims presented in original study	B. Originally reported analyses as reproduced by us	C. Our additional analyses contesting the original claims
1.1 Scarcity leads to greater focus.	Amongst participants who could not borrow, ‘poor’ participants spent significantly more time aiming the first shot of each level (log-transformed milliseconds, 8.08 ± 0.42) than did ‘rich’ participants (7.73 ± 0.39), *F*(1, 66) = 12.49, *P* < 0.001, *η*^2^(1) = 0.159, 95% CI [0.03, 0.31].	Although these ‘poor’ participants who could not borrow spent longer aiming their first shot of each level compared to the ‘rich’, they did not earn significantly more points on this shot compared to the ‘rich’ (‘poor’: 2.80 ± 0.99; ‘rich’: 2.91 ± 0.44), *F*(1, 31) = 0.13, *p* = 0.718, *η*^2^(1) =0 .004, 95% CI [0.00, 0.13]. They also did not earn significantly more points on their second (‘poor’: 1.93 ± 1.67; ‘rich’: 1.77 ± 0.48), *F*(1, 31) = 0.61, *p* = 0.440, *η*^2^(1) = .019, 95% CI [0.00, 0.19], or third shot of each level (‘poor’: 1.92 ± 0.74; ‘rich’: 1.73 ± 0.41), *F*(1, 31) = 0.73, *p* = 0.401, *η*^2^(1) = 0.024, 95% CI [0.00, 0.20]. (Additional comparisons are not possible because ‘poor’ participants who could not borrow only had three shots per level.) Further, there was no significant difference in points-per-shot between the ‘rich’ and the ‘poor’ when averaging across the first three shots of each level (‘poor’: 2.63 ± 0.19; ‘rich’: 2.53 ± 14), *F*(1, 31) = 0.13, *p* = 0.724, *η*^2^(1) = 0.004, 95% CI [0.00, 0.13].
1.2 Scarcity leads to greater focus.	The significantly longer aiming time of ‘poor’ participants who could not borrow emerged on the very first shot of the game (‘poor’: 8.19 ± 0.52; ‘rich’: 7.86 ± 0.52), *F*(1, 66) = 6.38, *p* = 0.014, *η*^2^(1) = 0.088, 95% CI [0.00, 0.23].	On the very first shot of the game, ‘poor’ participants who could not borrow did not earn significantly more points than their ‘rich’ counterparts (‘poor’: 1.85 ± 1.63; ‘rich’: 2.38 ± 2.90), *F*(1, 31) = 0.46, *p* = .503, *η*^2^(1) = .015, 95% CI [0.00, 0.17].
1.3 Scarcity leads to greater focus.	Among participants who could not borrow, the ‘poor’ earned significantly more points-per-shot (2.31 ± 0.60) than did the ‘rich’ (1.67 ± 0.37), F (1, 31) = 11.91, *P*= .002, *η*^2^(1) = 0.278, 95% CI [0.05, 0.48].	Among participants—both ‘rich’ and ‘poor’—who could not borrow, later shots on each level were significantly less profitable compared to earlier shots, *β* = −0.19, *t* = −14.85, *p* < .001 (−0.22, −0.17). ‘Poor’ participants employed significantly fewer shots on each level (‘poor’: 2.51 ± 0.45; ‘rich’: 5.67 ± 1.79), *F*(1, 31) = 57.60, *P* < 0.001, *η*^2^(1) = 0.650, 95% CI [0.42, 0.76], such that they did not shoot the later, less-profitable shots that ‘rich’ participants shot, resulting in a higher points-per-shot average.
2.1 Scarcity leads to over-borrowing	As a fraction of their budget, ‘poor’ participants borrowed significantly more shots (0.24 ± 0.15) than did the ‘rich’ (0.02 ± 0.05), *F*(1, 33) = 27.53, *P* < 0.001, *η*^2^(1) = 0.455, 95% CI [0.19, 0.62].	'Poor’ participants were significantly more likely to face the decision of whether to borrow or not (45% of all levels) compared to the ‘rich’ (2% of all levels), Χ^2^(1) = 184.42, *P* < 0.001. When considering only cases in which participants could have decided to borrow, ‘poor’ participants were, if anything, more likely to resist the temptation to borrow (31%) compared to ‘rich’ participants (0%), though this effect wasn’t significant, *Χ*^2^(1) = 3.79, *p* = 0.052. This was despite the fact that the option to borrow was arguably more attractive for ‘poor’ participants, as they had significantly more targets left to clear, on average, after exhausting their budget (2.54 ± 1.322) than ‘rich’ participants did (0.54 ± 0.637), *F*(1, 33) = 29.15, *P* < 0.001, *η*^2^(1) = 0.469, 95% CI [0.21, 0.63].
2.2 Scarcity leads to over-borrowing	'Rich’ participants performed similarly whether they could borrow (1.67 ± 0.37) or not (1.81 ± 0.53), while ‘poor’ participants fared significantly better when they could not borrow (2.31 ± 0.60) than when they could (1.77 ± 0.97) [scarcity × borrowing interaction: *F*(1, 64) = 4.04, *p* = 0.049, *η*^2^(1) = 0.059, 95% CI [0.00, 0.19].	Overall, across both ‘rich’ and ‘poor’ participants, performance was significantly negatively related to frequency of borrowing, *r*(33) = −0.688, *P* < 0.001, as well as the relative amount of borrowing, *r*(33) = −0.730, *P* < 0.001. Thus, borrowing was suboptimal for all participants, and ‘poor’ participants’ performance was particularly affected by borrowing because they faced the decision to borrow more often (see cell above).
3.1 When facing scarcity, a greater focus on current demands predicts neglect of future demands.	On levels where ‘poor’ participants borrowed, the average amount of time they spent aiming each shot was significantly positively correlated with how many shots they subsequently borrowed, *r*(38) = 0.43, *p* = 0.008. This could suggest that the attention they spent managing the scarce resource (in aiming) detracted from their attention to information outside the tunnel (interest rates for borrowing)	This correlation is not statistically significant when investigating all observations from participants (both ‘rich’ and ‘poor’) who could borrow, *r*(585) = 0.03, *p* = 0.452, all observations of the ‘poor’ who could borrow, *r*(122) = 0.06, *p* = 0.505, or all levels on which participants borrowed, *r*(47) = 0.15, *p* = 0.329.

The analyses presented next are all based on the original dataset^16^. But the findings and conclusions we report here also extend to a more recent replication of the original study conducted by the original authors^24^. We further confirmed these findings in a new conceptual replication study we conducted. For a summary of all analyses, see Table S1 in Supplementary Note 1.

### Re-analysis

Note that in their original publication, Shah et al.^16^ presented multiple studies using similar, but distinct designs, manipulating different kinds of budgets (e.g., time or number of guesses in a game). In our re-analysis reported here, we focus on Study 2, as the authors state that this study, together with Study 1 of the paper, which fails to replicate^23–25^, is the cornerstone for their account in ostensibly establishing the cognitive mechanism driving the behavioral results observed in all studies^16,24^. We also conducted re-analyses of Studies 4 and 5, which, according to the original authors, provide additional evidence for tunneling. Our re-analyses of these other studies follow the same pattern as our re-analysis of Study 2 (see Supplementary Note 5 for more details). Thus, our general critique of Study 2 from the original paper also applies to Studies 4 and 5. Note that we were unable to reanalyze the dataset for Study 3 because it did not contain information about the exact time points at which correct guesses occurred. Consequently, we could not compute our primary outcome measure, points-per-second (the equivalent of points-per-shot in Study 2), at the second-by-second level.

### Greater focus?

‘Poor’ participants who could not borrow were only given three shots on each level, while ‘rich’ participants were given 15 shots (Table 1). As originally reported, these ‘poor’ participants spent significantly more time aiming their shots and earned significantly more points-per-shot throughout the game than did ‘rich’ participants (Table 2). However, averaging points-per-shot across the game is potentially misleading because ‘rich’ participants on average took significantly more shots on a given level than did ‘poor’ participants (due to the smaller budget that ‘poor’ participants had; Table 2), and it is likely that for these additional shots taken by ‘rich’ participants, there were fewer targets remaining to hit on the level, necessarily decreasing their points-per-shot average. Thus, our additional analyses concentrated on the first three shots of each level, which both the ‘rich’ and the ‘poor’ could take (since ‘poor’ participants who could not borrow could only use up to three shots on each level).

**Fig. 1:**
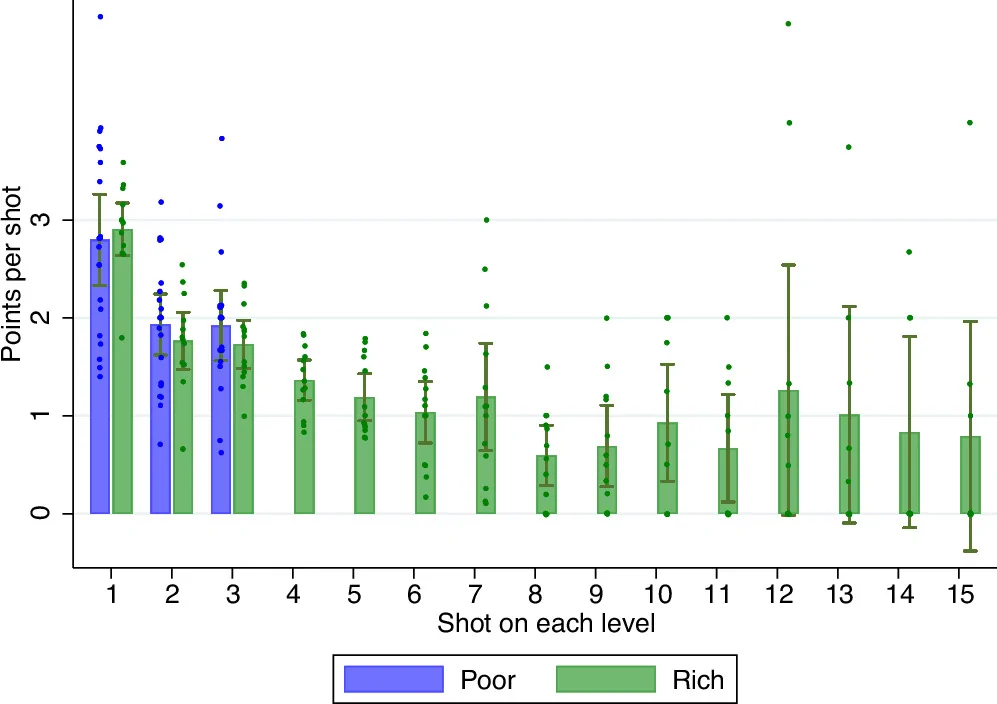
Points earned per shot on all shots individually comparing ‘rich’ (*n* = 28, in green) and ‘poor’ (*n* = 40, in blue) participants who could not borrow in the original study^16^. Note that ‘poor’ participants had only three shots per level. Data are presented as mean values ± SEM.

We reasoned that if the ‘poor’ were more focused than the ‘rich’, as argued by the original authors^16^, they should have been more efficient than the ‘rich’ for these first three shots. However, when looking at these first three shots per level, there were no significant differences in points-per-shot between the ‘poor’ and ‘rich’, neither when looking at the points-per-shot averaged across shots 1 to 3, nor when looking at each of the shots individually (all *p*s > .337; Fig. 1 and Table 2, Claim 1.1 and 1.2). Note that a sensitivity analysis revealed that the study had 80% power to detect effects with a minimum effect size of *η*^2^ = 0.106. The large-scale replication of Shah et al.^24^ had 80% power to detect effects down to a size of *η*^2^ = 0.008.

Using equivalence testing, we investigated whether indeed the performance of the ‘rich’ and the ‘poor’ was statistically equivalent when assuming a smallest effect size of interest (SESOI) of *d* = 0.374 (Following the guidelines of Lakens et al.^26^, the SESOI was set to the smallest effect size that the original study^16^ would have 33% power to detect. This SESOI was used for all equivalence tests reported here; see Supplemental Note 2 for more details). Insignificant results of equivalence testing revealed that the ‘poor’ and the ‘rich’ were not statistically equivalent either in their average performance of the first three shots or in their performance on each of the first three shots individually, all *p*s > 0.241. This suggests that the original study^16^ was underpowered to allow any conclusion. However, Shah and colleagues’ large-scale replication^24^ was sufficiently powered to reveal that there was no meaningful difference, in that study, in performance between the ‘rich’ and the ‘poor’ on these first three shots, all *p*s < 0.005.

Thus, the longer aiming time of ‘poor’ participants did not actually translate into better outcomes for those shots. This casts doubt on whether ‘poor’ participants’ longer aiming time reflected their greater focus on the task, as originally claimed, or whether this could be explained by other factors, such as increased inattention or hesitation among ‘poor’ participants. Even if the longer aiming time of ‘poor’ participants reflected increased focus, it was not the kind of increased focus that led to better performance at shooting.

How, then, did ‘poor’ participants ultimately earn more points-per-shot throughout the game? As alluded to above, on each level, earlier shots were significantly more profitable for all participants, presumably because there were more targets to hit (Fig. 1 and Table 2, Claim 1.3). Across conditions, participants scored an average of 2.81 points on their first shot of each level, but this dropped to 1.80 points by shot 3, and to 0.69 points on shot 15 for ‘rich’ participants. Thus, because ‘poor’ participants who could not borrow had only three shots on each level, they could not shoot the later, less-profitable shots that ‘rich’ participants employed, which resulted in a higher points-per-shot average for ‘poor’ participants. Due to the diminishing returns of shots used on each level, the optimal strategy for all participants (in terms of optimizing points-per-shot) would have been to move onto the next level after firing only one shot (since there were infinite levels, see Table 1). However, our additional analyses show that in the condition without borrowing, neither the ‘poor’ (shots used per level: *M* = 2.51, *SD* = 0.45), nor the ‘rich’ (shots used per level: *M* = 5.67, *SD* = 1.79) consistently employed this strategy.

In sum, our additional analyses suggest that the better performance of the ‘poor’ compared to the ‘rich’ in the original experiment did not stem from their greater focus on the task, as proposed by the original authors and repeated in subsequent discussions of these findings^6,7^. Instead, the ‘poor’ achieved a higher point-per-shot average due to a combination of two elements of the game’s design: first, the fact that returns diminished with each shot per level, and second, the fact that the ‘poor’ were restricted to taking only three shots per level (Table 1).

### Attentional neglect?

As originally reported, ‘poor’ participants who could borrow from future levels at a 100% interest rate borrowed relatively more than ‘rich’ participants who could borrow, which hurt their performance in the game. Shah and colleagues^16^ argued that these ‘poor’ participants borrowed more often because they were less likely than ‘rich’ participants to pay attention to the high costs of borrowing (i.e., they suffered from attentional neglect). Our re-analyses, however, reveal that ‘poor’ participants were significantly more likely to face the decision of whether to borrow, as they were more likely to exhaust their budget—which was a prerequisite for borrowing (Table 1)—while still having targets left to hit (Table 2, Claim 2.1). Because ‘poor’ participants were only given three shots per level, they quickly ran out of shots and received the option to borrow on 45% of levels. By contrast, ‘rich’ participants, who were given 15 shots per level, only ran out of shots and received the option to borrow on 2% of all levels.

If ‘poor’ participants were more likely to borrow due to attentional neglect, one might expect them to have borrowed more frequently than ‘rich’ participants even after controlling for this difference in base rate. Yet, our additional analyses show that when examining only those cases in which participants had the opportunity to borrow, ‘rich’ participants always borrowed, while ‘poor’ participants only borrowed in 69% of cases (Table 2, Claim 2.2). Hence, the data do not support the interpretation that ‘poor’ participants borrowed more frequently due to attentional neglect of information outside the tunnel. If anything, the ‘poor’ were less likely to fall prey to high-interest borrowing opportunities than the ‘rich’ (Table 2, Claim 2.2). Note that to further support the hypothesis that greater focus on current demands goes hand-in-hand with attentional neglect of information ‘outside the tunnel’, Shah and colleagues^16^ also reported a significant positive correlation between ‘poor’ participants’ aiming times and how many shots they subsequently borrowed, on levels where ‘poor’ participants did borrow. Yet it is not clear why the authors only analyzed this specific subset of observations. When considering any other subset of observations, or all observations together, there is no significant correlation between aiming times and shots borrowed (Table 2, Claim 3.1). Further, the correlation does not replicate in Shah et al.’s large-scale replication study from 2019^24^.

### Conceptual replication study

We note that comparing the behavior of the ‘poor’ vs. ‘rich’ on levels in which they could borrow is potentially misleading because only 40% of ‘rich’ participants (vs. 90% of ‘poor’ participants) ever faced the decision of whether to borrow. It is possible that this subset of ‘rich’ participants differed from ‘rich’ participants who never faced this decision, e.g., in their level of engagement. To address this issue, we conducted a conceptual replication experiment, which used the same general setup as the ‘Angry Blueberries’ study, while holding the base rate frequencies in which ‘poor’ and ‘rich’ participants could engage with information outside the hypothesized tunnel constant. To this end, instead of providing participants with an option to borrow, they were given an option to use their shot(s) on a level to purchase insurance against a future ‘drought’ that could potentially destroy additional points that they would receive. Crucially, both ‘rich’ and ‘poor’ participants in our study equally often received the opportunity to purchase this insurance, namely on the first ten levels of the game. Furthermore, we varied the expected value of the insurances: while eight of the insurances had positive expected values, we also included two ‘scam’ insurances, in which the benefits of the insurance did not exceed its costs. This means that a risk-neutral agent should buy only the eight insurances with positive expected value.

We note that, just as in the case of the borrowing option presented in the original study, the insurance purchase option in our conceptual replication study had an intertemporal component: While participants had to pay the costs of purchasing insurance by sacrificing shots on their current level, the gains would only be realized in the future. Recall that scarcity theory claims that scarcity leads individuals to focus on immediate, pressing needs, while neglecting potential effects for their future well-being. Thus, by this account, the ‘poor’ should generally not purchase insurance, reflecting their attentional neglect of future outcomes, which means that they could miss out on insurance with positive expected value that could help them in the future. The ‘rich’, however, should selectively purchase only insurance with positive expected value, as they should recognize insurance with negative expected value as exploitative. As such, scarcity theory predicts that there should be a difference between the ‘poor’ and the ‘rich’ particularly in their purchasing of insurances with a positive expected value.

Contrary to this hypothesis, we did not find a significant difference in the likelihood to purchase insurance with a positive expected value between the ‘poor’ (4.74 ± 2.84) and ‘rich’ participants (4.92 ± 2.92) in our conceptual replication study, *F*(1, 172) = 0.17, *p* = 0.682, *η*^2^(1) = 0.001, 95% CI [0.00, 0.03]. Indeed, equivalence testing revealed that the likelihood of purchasing insurance with a positive expected value was statistically equivalent between the ‘rich’ and the ‘poor’, *t*(169.56) = −2.052, *p* = 0.021, given our SESOI of *d* = 0.374 (see above). Moreover, comparing purchasing rates of insurances with negative expected value revealed that, if anything, ‘poor’ participants were *more discerning* in their decision making: they were significantly less likely than ‘rich’ participants to purchase ‘scam’ insurances in which costs were higher than expected returns, *F*(1, 172) = 8.24, *p* = 0.005, *η*^2^(1) = 0.046, 95% CI [0.00, 0.12]. These findings provide additional evidence against the hypothesis that scarcity leads to attentional neglect of potentially beneficial information that does not pertain to present needs.

Importantly, our conceptual replication study also replicated the original analyses of Shah et al.^16,24^, as well as the additional analyses we conducted supporting our alternative interpretation (for an overview of all results, see Table S1 in the Supplementary Note 1). In particular, we found that, compared to the ‘rich’, the ‘poor’ took significantly more time aiming, *F*(1, 172) = 10.93, *p* = 0.001, *η*^2^(1) = 0.060, 95% CI [0.01, 0.14], though this difference did not reach significance on the very first shot of the game (‘poor’: 8.46 ± 0.57; ‘rich’: 8.35 ± 0.69), *F*(1, 172) = 1.30, *p* = 0.256, *η*^2^(1) = 0.007. ‘Poor’ participants also earned more points-per-shot throughout the game, *F*(1, 172) = 21.95, *p* < 0.001, *η*^2^(1) = 0.113, 95% CI [0.04, 0.21]. However, we again found evidence for our alternative interpretation, as there was no meaningful difference in points-per-shot between the ‘poor’ and the ‘rich’ on the first three shots of each level, all *p*s > 0.439. Performing equivalence tests revealed statistical equivalence between the ‘rich’ and the ‘poor’ on the first shot, *t*(170.6) = −1.851, *p* = 0.033, as well as the second, *t*(129.53) = 1.668, *p* = 0.049, and third shot, *t*(80.7) = 1.980, *p* = 0.026. Rather than an increase in attention in the ‘poor’ leading to better performance, later shots were significantly less profitable for all participants, *β* = −29, *t* = −34.28, *p* < 0.001 (−0.30, −0.27). Because ‘poor’ participants were restricted to taking the first three shots of each level, they could not employ the less-profitable, later shots that ‘rich’ participants used, resulting in a higher point-per-shot average for ‘poor’ participants. Thus, our conceptual replication study was able to replicate the effects of the scarcity manipulation, setting it up to critically investigate whether scarcity causes attentional neglect, which we found no evidence of.

## Discussion

Scarcity theory proposes that individuals who are experiencing scarcity exhibit a tunneling effect–allocating greater attention toward managing their resources in the present moment (leading to positive outcomes), to the neglect of other potentially beneficial information that pertains to the future (leading to negative outcomes)^6,7,16^. Though widely accepted and highly influential, support for the tunneling hypothesis primarily derives from a single landmark study^16^. Yet our re-analysis of this study presented several findings suggesting that both the positive (better performance) and negative (overborrowing) behavioral effects of scarcity observed in this study could be more parsimoniously explained by the structure of the game that participants played, as opposed to a psychological shift in attention (i.e., tunneling), as it is typically interpreted. We do not contest the effect of the manipulation on behavioral outcomes (such as improved performance and increased borrowing among the ‘poor’); however, we suggest that these outcomes were driven by constraints on how ‘poor’ vs. ‘rich’ participants could play the game, rather than by a shift in attention caused by scarcity. In what follows, we review our findings, contextualize them within the broader literature on scarcity and attention, and discuss how the theoretical construct of tunneling could be clarified and tested further.

Regarding the hypothesis that scarcity leads to greater focus on managing scarce resources, our re-analysis found that although ‘poor’ participants spent significantly more time aiming their shots, this did not translate into better performance on those shots, conflicting with the original authors’ claim that “this engagement had some benefits for the poor” (p. 684)^16^. Our re-analysis instead suggested that ‘poor’ participants earned significantly more points-per-shot throughout the game because the shots they were restricted to taking (the first three shots of each level) were necessarily more profitable than the later shots only available to the ‘rich’ (shots 4–15), due to the structure of the game in which there were diminishing returns of later shots on each level for all participants. Note that these results also replicate in the large-scale replication study^24^ conducted by the original authors, as well as in our conceptual replication study. Furthermore, applying this logic to the data from Studies 4 and 5 yields comparable results (see Supplementary Note 5). Our alternative interpretation stands in opposition to the presentation of these findings in Mullainathan and Shafir’s influential book on scarcity^7^, which claimed that the ‘poor’ profited from an “accuracy advantage” (p. 26); indeed, our re-analysis showed that the accuracy of each of the first three shots individually did not meaningfully differ between the two participant groups. Of course, ‘poor’ participants could in principle have taken longer than ‘rich’ participants to aim their shots due to greater focus on the task, but absent a connection to better shooting performance, this interpretation is only one of many. It is equally plausible, given the data, that ‘poor’ participants were more inattentive or hesitant than ‘rich’ participants.

With regards to the claim that scarcity causes attentional neglect of potentially beneficial information unrelated to the present, our re-analysis showed that ‘poor’ participants in the original study did not overborrow because, as the original authors claimed, the “focus on some problems leads to neglect of others” (p. 684)^16^, but rather because they were significantly more likely than ‘rich’ participants to face the decision of whether to borrow. Because borrowing was counterproductive for all participants, and the ‘poor’ engaged in borrowing more frequently due to more often facing this decision, having the option to borrow hurt the performance of the ‘poor’, but not of the ‘rich’. Consequently, the data do not support the interpretation in Mullainathan and Shafir’s book that “the powerful impulse to borrow […] is a direct consequence of tunneling” (p. 115)^7^, according to which the effect was driven by attentional differences between the ‘rich’ and the ‘poor’ towards the cost of borrowing. Indeed, when examining only those cases in which participants had the opportunity to borrow, ‘poor’ participants were, if anything, less likely to borrow than ‘rich’ participants, which is unexpected on the account that the ‘poor’ suffered from attentional neglect. The detrimental effects of borrowing on ‘poor’ participants’ performance, again, were a consequence of how the game was structured: ‘rich’ participants exhausted their budget and received the option to borrow on only 2% of all levels, while the ‘poor’ received this option on about half of all levels. (Our re-analyses of Studies 4 and 5 also support this argument: ‘poor’ participants in those studies were consistently more likely than ‘rich’ participants to encounter the decision of whether to borrow, but were no more likely to borrow than ‘rich’ participants when controlling for these base rates; see Supplementary Note 5).

Evidence from our conceptual replication study further supported this alternative interpretation. In this study, we held the base rate frequencies with which ‘rich’ and ‘poor’ participants were presented information ‘outside of the tunnel’ constant, by equally often giving participants the option to sacrifice their current resources toward purchasing insurance to protect against the future loss of points. Contrary to the hypothesis that scarcity causes individuals to focus on current needs while neglecting the future—which would predict that ‘rich’ participants should have purchased positive expected value insurance more so than ‘poor’ participants—there were no meaningful differences in ‘rich’ and ‘poor’ participants’ propensity to buy insurance with positive expected value. If anything, it was ‘poor’ participants who exhibited more attention on the insurance options, as they were significantly less likely to purchase ‘scam’ insurance. Although proponents of the tunneling hypothesis might respond that decisions about purchasing insurance fall inside of the scarcity-induced attentional tunnel, while borrowing decisions fall outside of it, both kinds of decisions share an intertemporal component in which decisions in favor of maintaining current resources take away from resources in the future. Indeed, prior work has invoked the psychological effects of scarcity to explain underinsurance among the poor^3^.

More generally, the scarcity theory lacks a clear definition of what kinds of information and decisions should fall within or outside of the ‘tunnel’. Shah and colleagues^16^ proposed that scarcity shifts attention to “pressing expenses today” and away from the future (p. 683). This definition would restrict the application of tunneling to behaviors and decisions with an intertemporal component. An alternative definition can be found in the review paper of de Bruijn and Antonides^6^, in which tunneling is characterized by “an attentional focus on poverty-related issues that enhances resource efficiency” (p. 10). One problem with the latter definition is that the scope of “poverty-related issues” is unclear. For example, decisions about borrowing could be seen as a poverty-related behavior that pertains to resource efficiency, and could arguably fall within the tunnel according to this definition. Absent a clear definition of what should fall within vs. outside the tunnel, it is impossible to falsify the theory, because it becomes possible, post-hoc, to attribute any decision that is enhanced among the poor to greater focus, and to attribute any decision that is impaired to attentional neglect.

Greater clarity is also needed in specifying the processes through which scarcity is hypothesized to drive attention. A first possibility is that scarcity creates a unique mindset that leads individuals experiencing scarcity to approach a task differently than individuals experiencing abundance approach the same task, resulting in different attentional patterns. We take Shah and colleagues^16^ as arguing for this mindset-based process, as they write that “resource scarcity creates its own mindset, changing how people look at problems and make decisions” (p. 682). But we have presented evidence here that the behavioral differences Shah and colleagues^16^ observed between ‘rich’ and ‘poor’ participants in their study (i.e., higher points-per-shot and overborrowing by ‘poor’ participants) did not arise from attentional differences caused by scarcity but instead from other elements of the game’s structure (i.e., because ‘poor’ participants were restricted to taking more profitable shots and more often faced the choice of borrowing at a high interest rate).

We are not aware of any other empirical evidence supporting the proposal that the experience of scarcity leads to a unique mindset toward a task that results in attentional tunneling. Some correlational evidence from changes around payday suggests that people are generally more likely to notice unexpected events (unrelated to resources, e.g., noticing the appearance of a gorilla in a video) when experiencing scarcity more strongly (i.e., right before payday)^27^. Although these data suggest that scarcity may change how people allocate attention, they do not directly support the tunneling hypothesis, which holds that scarcity induces greater attentional focus specifically on scarce resources, and that this greater focus takes away from attention to other potentially important information. Similarly, other studies that have been interpreted as providing support for the tunneling hypothesis only find that scarcity leads to greater focus (e.g., better memory for prices^7^, less susceptibility to framing effects^15^, better performance on word-search tasks^12,21,22^), without demonstrating any form of attentional neglect.

A second possible way in which scarcity could shape attention is not by changing an individual’s mindset toward a task, but instead by changing the basic nature of the task that they face, with changes to their attention resulting from changes to the task. For example, when ordering food in a restaurant, having a tight budget means that an individual must calculate the exact costs of their order, which they would not have to do (or would not have to do as carefully) if their budget were unrestricted. This task difference might very well lead to attentional differences: e.g., an individual with a restricted budget might be more likely to closely scrutinize the prices of items on the menu than an individual who lacks such restrictions. Indeed, Tomm and Zhao^28^ conducted an eye-tracking study involving a similar scenario, and found that participants who were randomly assigned a small budget dedicated a larger proportion of their time focusing on prices on a menu, while spending a smaller proportion of their time focusing on information on how to obtain a discount (though participants with a smaller budget were no less likely to ask for the discount or report seeing it, preventing an interpretation of attentional neglect). We suggest that ‘rich’ and ‘poor’ participants in this study faced different tasks, and deployed their visual attention in different ways as a result. However, this does not tell us whether individuals under scarcity adopt a unique mindset that causes them to approach the same task differently than individuals who are not experiencing scarcity.

As the preceding discussion suggests, while existing studies in this literature suggest that scarcity may increase attention toward scarce resources, they fail to show that scarcity causes attentional neglect and, in turn, the negative behavioral effects of tunneling, which have been held up as explanations of counter-productive behaviors among the poor, including overborrowing and underinsurance^6,29^. Future work is thus needed to demonstrate whether scarcity creates an attentional tunneling effect—including both its positive and negative effects—and to understand the specific processes through which scarcity may influence attention and behavior. As our re-analyses illustrate, it is crucial that any experimental manipulation of scarcity ensures that its observed behavioral effects genuinely stem from attentional shifts, rather than from alterations within the decision spaceof the 'poor' vs. 'rich'.

Although we have argued that the ‘Angry Blueberries’ experiment of Shah et al.^16,24^ does not provide evidence for the tunneling hypothesis, it might mirror important structural effects that scarcity has on people. Clearly, the environments we inhabit differ depending on one’s socio-economic status, and different structural factors of these environments shape our behavior. From this perspective, much of what people in poverty face may not simply be a case of decision-making shaped by a unique mindset; rather, it is constrained within a fundamentally different decision space than that of the rich. For example, in Shah and colleagues’ study^16,24^, as well as in the real world, the ‘poor’ face the decision of whether to borrow at inflated interest rates more often than the ‘rich’. As such, the experiment might tell us something about how having the option to borrow at high interest rates differentially affects the ‘poor’ and the ‘rich’, even though this disparity cannot be attributed to the proposed attentional mechanism of tunneling. Importantly, this effectively illustrates the importance of considering structural constraints when studying decision-making in contexts of adversity—an aspect largely neglected by the field. Moving forward, researchers could examine these structural constraints and how they shape behavior differently depending on an individual’s position within the social hierarchy.

While our reanalyses and conceptual replication study provide evidence that the behavioral effects reported by Shah et al.^16,24^ cannot be attributed to the proposed attentional tunneling mechanism, several limitations should be noted. Both the original studies and our replication rely on artificial laboratory paradigms that may not capture the complexity of real-world poverty, limiting external validity—although such approaches are common given the ethical and practical constraints of manipulating poverty in the field. In addition, both the original studies and our replication study used online samples recruited via MTurk, which raises general concerns about data quality and platform-specific influences. These considerations suggest caution regarding generalizability. Still, regardless of whether the paradigm of Shah et al.^16,24^ is seen as representative of real-world conditions, our reanalysis demonstrates that the authors’ original claims about their findings do not hold up.

Recent work has contested the replicability of research on the psychological consequences of scarcity^30,31^. By providing an alternative interpretation of a foundational study on the attentional and behavioral effects of scarcity, our findings provide an example of how study findings that do replicate can also be misleading. When trying to mimic the experience of scarcity in the lab to probe for attentional mechanisms, structural factors that covary with scarcity should be taken into account. Ultimately, a better understanding of the cognitive and behavioral consequences of experiencing scarcity will be important for informing policy recommendations about how to combat poverty around the globe^32,33^.

## Methods

All data were analyzed using STATA, version BE 17.0, except for equivalence testing, which was performed in R version 4.4.3 (2025-02-28, “Trophy Case”). All our analyses follow the analyses reported by Shah et al.^16,23^ to allow for direct comparison between the results, meaning that we used ANOVAs to report on mean differences between groups. All statistical tests were two-sided.

### Re-analysis of Shah et al.^16,24^

The original study^16^ had a sample size of *N* = 68, while the large-scale replication conducted by the original authors^24^ included a much larger sample of *N* = 1010. Neither sample was representative of the U.S. population. Please note that we have no information on participant dropout within the original publications^16,24^. Therefore, we cannot rule out potential bias due to systematic dropouts or incomplete randomization in that context. This issue also applies to our conceptual replication study, as we used the same program for the Angry Blueberries game, which did not retain information on dropouts. In their large-scale replication^24^, the authors note that participants were excluded from the analyses if they were missing subject ID numbers or if an error in the game led to an incorrect allotment of blueberries, such as if the game ended prematurely before they could use their blueberries or allowed them to continue playing after their blueberries were exhausted. After applying these exclusion criteria, 36 participants were removed, resulting in a final sample of 974 participants for the analyses. Within this final sample, participants reported a mean age of 34 years, with 56% identifying as female, and 44% as male (note that demographic information was missing for two participants). The randomization led to 423 participants in the ‘rich’ condition and 551 participants in the ‘poor’ condition. Informed consent was obtained from all participants prior to participation in both studies, and ethical approval was obtained from the respective institutional review boards at the University of Chicago for the original study^16^ (Protocol No. 12-1899) and Princeton University for the large-scale replication study^24^ (Protocol No. 4111). Finally, we note that we have no demographic information on the participants from the original study^16^.

### Conceptual replication study

For our conceptual replication study, we used the original ‘Angry Blueberries’ game^16^, adapting it to our new specifications. To ensure reasonable data quality, we included bot-detection questions at the beginning of the study, as well as a comprehension check—consisting of three multiple-choice questions—administered before participants entered the Blueberry Game. Participants who failed these comprehension checks were excluded from further participation (for the exact wording, see our materials on OSF).

On the first 10 levels of the game, an insurance option was displayed in the upper left corner of the screen. Participants were informed that they would have an additional pineapple field that was independent of their performance in the game but would nevertheless count toward their final points and, consequently, their payoffs. They were told that this field could be destroyed by a drought, but that they could purchase insurance against such a drought. Participants could click on a button to buy the insurance. Participants were informed that the probability of the drought destroying the points was set to 50%. The information on the insurance contained the prices of buying the insurance, as well as the amount of points that would be insured. The prices and the potentially insured points were relative to participants’ budgets. The price of the insurance was defined as the number of shots participants had to invest to purchase it. Furthermore, we varied the expected value of the insurances: eight of the insurances had positive expected values, whereas two had negative expected values, meaning that expected benefits did not exceed the costs of these insurances. Because participants could not lose more pineapples than they had in their pineapple field, this setup ensured that they did not risk falling below zero and that all participants were therefore incentivized to simply maximize their points. After finishing their budget, participants were asked to complete measures of their time and risk preferences (based on Falk et al.^34^; not reported here).

We collected data from 174 participants after they provided informed consent, using the reprogrammed ‘Angry Blueberries’ game hosted on a university server. We used Amazon’s MTurk as a recruitment platform to remain as consistent as possible with the original studies. Participants were randomly assigned to either the ‘rich’ condition (83 participants) or the ‘poor’ condition (91 participants). We initially recruited 200 participants, of whom 26 did not complete the game. Participants received a flat fee of $3.00 and additionally earned $0.02 per point accumulated during the game, resulting in a performance-based bonus ranging between $0.46 and $7.03 (*M* = $3.20, *SD* = $2.17). The study was approved by the review board of the University of California, Berkeley (CPHS Protocol Number: 2013-08-5546). Participants self-reported their gender identity (42.53% female, 56.32% male, 1.15% preferred not to disclose). No gender-specific analyses were conducted due to the absence of a priori hypotheses regarding gender differences. Participants also self-reported their age (*M* = 40.2 years). Age was collected to characterize the sample, and no age-specific analyses were performed. We report how we determined our sample size, all manipulations, and all measures in the study in our pre-registration. For all data, materials, analysis scripts and the pre-registration from November 1, 2021, see osf.io/8pzw3.

Please note that we deviated from our pre-registration in two ways. First, we pre-registered a sample of 200 participants from MTurk, but were only able to sample a total of 174 participants due to budgetary constraints at the time. Note that in our pre-registration, we explain that replicating the most central effect would require a sample size of *N* = 90 to detect the medium effect size reported in Shah et al. (2019) with 80% power. Second, we did not employ the pre-registered analysis plan. We had planned to conduct multi-level analyses, but did not consider the fact that participants who use fewer points per level would end up having more observations counted into the results compared to participants who use more shots per level. As such, we employed the same data analytic approach used by the original authors^16^. This also allows for easier comparison of results. For additional information, please refer to the Supplement.

### Reporting summary

Further information on research design is available in the Nature Portfolio Reporting Summary linked to this article.

## Supplementary information

Supplementary Information

Reporting Summary

Transparent Peer Review file

## Data Availability

The data for Shah et al.’s original study^16^ and replication study^24^ are both available under https://osf.io/vzm23/overview. The data for our conceptual replication study have been deposited under https://doi.org/10.17605/OSF.IO/8PZW3.

## Code availability

Our analysis scripts re-analyzing all data available from Shah et al.’s two studies^16,24^ can be accessed at https://doi.org/10.17605/OSF.IO/9M7KP. For the analysis scripts concerning our conceptual replication study, please see https://doi.org/10.17605/OSF.IO/8PZW3.

## References

[1] Annie, E. Casey Foundation. *Strengthening Rural Families: The High Cost of Being Poor* https://www.aecf.org/resources/strengthening-rural-families-the-high-cost-of-being-poor (2004).

[2] Escobar, M.-L., Griffin, C. C. & Shaw, R. P. *The Impact of Health Insurance in Low- and Middle-Income Countries*. (Brookings Institution Press, 2011).

[3] Schneider, P. Why should the poor insure? Theories of decision-making in the context of health insurance. *Health Policy Plan* **19**, 349–355 (2004). 10.1093/heapol/czh050 15459160

[4] Moynihan, D. P. & Sundquist, J. L. *Perspectives on Poverty*. vol. 1 (Basic Books, 1969).

[5] Lawrance, E. C. Poverty and the rate of time preference: evidence from panel data. *J. Polit. Econ.* **99**, 54–77 (1991).

[6] de Bruijn, E.-J. & Antonides, G. Poverty and economic decision making: a review of scarcity theory. *Theory Decis.* **1**, 33 (2021).

[7] Mullainathan, S. & Shafir, E. *Scarcity: The True Cost of Not Having Enough*. (Penguin Books, 2013).

[8] Park, Y. R. et al. The psychology of poverty: current and future directions. *Curr. Dir. Psychol. Sci.* **34**, 21–28 (2025).

[9] de Bruijn, E.-J. & Antonides, G. Determinants of financial worry and rumination. *J. Econ. Psychol.* **76**, 102233 (2020).

[10] Johar, G., Meng, R. & Wilcox, K. Thinking about financial deprivation: rumination and decision making among the poor. *NA-Adv. Consum. Res*. *43* (2015).

[11] Shah, A. K., Zhao, J., Mullainathan, S. & Shafir, E. Money in the mental lives of the poor. *Soc. Cogn.* **36**, 4–19 (2018).

[12] Lichand, G. & Mani, A. Cognitive droughts. *SSRN Sch.* 10.2139/ssrn.3540149 (2020). Paper at.

[13] Mani, A., Mullainathan, S., Shafir, E. & Zhao, J. Poverty impedes cognitive function. *Science* **341**, 976–980 (2013). 10.1126/science.1238041 23990553

[14] Ong, Q., Theseira, W. & Ng, I. Y. H. Reducing debt improves psychological functioning and changes decision-making in the poor. *Proc. Natl. Acad. Sci.* **116**, 7244–7249 (2019). 10.1073/pnas.1810901116 PMC6462060 30910964

[15] Shah, A. K., Shafir, E. & Mullainathan, S. Scarcity frames value. *Psychol. Sci.* **26**, 402–412 (2015). 10.1177/0956797614563958 25676256

[16] Shah, A. K., Mullainathan, S. & Shafir, E. Some consequences of having too little. *Science* **338**, 682–685 (2012). 10.1126/science.1222426 23118192

[17] Pollack, H. Being poor changes your thinking about everything. (Washington Post*,* 2013).

[18] Rosenberg, T. Escaping the cycle of scarcity. *The New York Times* (2013).

[19] Schulte, B. How busyness leads to bad decisions. (BBC, 2019).

[20] World Bank. *World development report 2015: Mind, society, and behavior*. (World Bank, 2014).

[21] Gutierrez, H. C. & Hershey, D. A. Impact of retirement worry on information processing. *J. Neurosci. Psychol. Econ.* **6**, 264–277 (2013).

[22] Shapiro, G. K. & Burchell, B. J. Measuring financial anxiety. *J. Neurosci. Psychol. Econ.* **5**, 92 (2012).

[23] Shah, A. K., Mullainathan, S. & Shafir, E. An opportunity for self-replication. *Nat. Hum. Behav.* **2**, 603–603 (2018). 10.1038/s41562-018-0405-5 31346269

[24] Shah, A. K., Mullainathan, S. & Shafir, E. An exercise in self-replication: replicating Shah, Mullainathan, and Shafir (2012). *J. Econ. Psychol.* **75**, 102127 (2019).

[25] Camerer, C. F. et al. Evaluating the replicability of social science experiments in Nature and Science between 2010 and 2015. *Nat. Hum. Behav.* **2**, 637–644 (2018). 10.1038/s41562-018-0399-z 31346273

[26] Lakens, D., Scheel, A. M. & Isager, P. M. Equivalence testing for psychological research: a tutorial. *Adv. Methods Pract. Psychol. Sci.* **1**, 259–269 (2018).

[27] Schmitt, S. Y. & Schlatterer, M. G. Poverty and limited attention. *Econ. Hum. Biol.* **41**, 100987 (2021). 10.1016/j.ehb.2021.100987 33601111

[28] Tomm, B. M. & Zhao, J. Scarcity captures attention and induces neglect: eyetracking and behavioural evidence. in *CogSci* 1199–1204 (2016).

[29] Adamkovič, M. & Martončik, M. A review of consequences of poverty on economic decision-making: a hypothesized model of a cognitive mechanism. *Front. Psychol.* **8**, 1784 (2017). 10.3389/fpsyg.2017.01784 PMC5641572 29075221

[30] O’Donnell, M. et al. Empirical audit and review, and an assessment of evidentiary value in research on the psychological consequences of scarcity. *Proc. Natl. Acad. Sci.* **118**, e2103313118 (2021). 10.1073/pnas.2103313118 PMC8612349 34711679

[31] Shah, A. K., Zhao, J., Mullainathan, S. & Shafir, E. A scarcity literature mischaracterized with an empirical audit. *Proc. Natl. Acad. Sci.* **120**, e2206054120 (2023). 10.1073/pnas.2206054120 PMC10293841 37339220

[32] Frankenhuis, W. E. & Nettle, D. The strengths of people in poverty. *Curr. Dir. Psychol. Sci.* **29**, 16–21 (2020).

[33] Sheehy-Skeffington, J. Inequality from the bottom up: Toward a “Psychological Shift” model of decision-making under socioeconomic threat. in *The social psychology of inequality* 213–231 (Springer, 2019)

[34] Falk, A., Becker, A., Dohmen, T., Huffman, D. & Sunde, U. The preference survey module: a validated instrument for measuring risk, time, and social preferences. *Manag. Sci.* **69**, 1935–1950 (2023).

