# Supplementary material for: Revisiting evidence for an attentional tunneling effect caused by scarcity
Source: Nat Commun. 2026 Sep 26;17:10233. doi: 10.1038/s41467-026-78074-y (PMC13615970; doi:10.1038/s41467-026-78074-y)
Supplement: Supplementary file 1 [file 41467_2026_78074_MOESM1_ESM.pdf]

Supplemental Materials for

"Revisiting evidence for an attentional tunneling effect caused by scarcity"

Fiona tho Pesch[1,2,3]*, Antonia F. Langenhoff[4,5] & Mahesh Srinivasan[4]

[1]Department of Psychology, University of Cologne, Germany

[2]Département d'études cognitives, Ecole normale supérieure – PSL, Paris, France

[3]Max Planck Institute for Research on Collective Goods, Bonn, Germany

[4]Department of Psychology, University of California Berkeley, USA

[5]Department of Psychology, Stanford University, USA

*Corresponding author. Email: fiona@thopesch.de

Table of contents

Supplementary Note 1: Results of Shah et al. (2012, 2019) and of our conceptual replication study - Original and additional analyses 2

Supplementary Note 2: Equivalence testing and power considerations 6

Supplementary Note 3: Sensitivity analyses 11

Supplementary Note 4: Additional preregistered analyses for our conceptual replication study 14

Supplementary Note 5: Re-Analyses of Studies 4 and 5 from Shah et al. (2012, 2019) 15

Supplementary Note 1: Results of Shah et al. (2012, 2019) and of our conceptual replication study - Original and additional analyses

Table S1. *Primary claims of the original interpretation (Column A), original analyses supporting those claims as reproduced by us (Column B1-B3)*, *and our additional analyses contesting those claims (Column C1-C3). All statistical tests were two-sided. No adjustments were made for multiple comparisons. Exact P values, test statistics (F, t, or χ²), corresponding degrees of freedom, effect sizes, and 95% confidence intervals are provided in the table.*

**A. Finding described in original study**	**B1. Reproduction of reported analyses in Shah et al. (2012)**	**B2. Reproduction of reported analyses in Shah et al. (2019)**	**B3. Reproduction of reported analyses from our conceptual replication study**	**C1. Our additional analyses on Shah et al. (2012)**	**C2. Our additional analyses on Shah et al. (2019)**	**C3. Our additional analyses on our conceptual replication study**
Scarcity leads to greater focus.	Amongst participants who could not borrow, 'poor' participants spent significantly more time aiming the first shot of each level (log-transformed milliseconds, 8.08 ± .42) than did 'rich' participants (7.73 ± .39), *F*(1, 66) = 12.49, *p* < .001, $\eta^2(1)$ = .159, 95% CI [0.03, 0.31].	Amongst participants who could not borrow, 'poor' participants spent significantly more time aiming the first shot of each level (log-transformed milliseconds, 8.11 ± .45) than did 'rich' participants (7.81 ± .40), *F*(1, 973) = 115.96, *p* < .001, $\eta^2(1)$ = .106, 95% CI [0.07, 0.14].	'Poor' participants spent significantly more time aiming the first shot of each level (log-transformed milliseconds, 8.32 ± .44) than did 'rich' participants (8.09 ± .46), *F*(1, 172) = 10.93, *p* = .001, $\eta^2(1)$ = .060, 95% CI [0.01, 0.14].	Although these 'poor' participants who could not borrow spent longer aiming their first shot of each level comparted to the 'rich', they did not earn significantly more points on this shot compared to the 'rich' ('poor': 2.80 ± .99; 'rich': 2.91 ± .44), *F*(1, 31) = .13, *p* = .718, $\eta^2(1)$ = .004, 95% CI [0.00, 0.13]. They also did not earn significantly more points on their second ('poor': 1.93 ± 1.67; 'rich': 1.77 ± .48), *F*(1, 31) = .61, *p* = .440, $\eta^2(1)$ = .019, 95% CI [0.00, 0.19], or third shot of each level ('poor': 1.92 ± .74; 'rich': 1.73 ± .41), *F*(1, 31) = .73, *p* = .401, $\eta^2(1)$ = .024, 95% CI [0.00, 0.20]. Additional comparisons are not possible because 'poor' participants who could not borrow only had three shots per level. Further, there was no significant difference in points-per-shot between the 'rich' and the 'poor' when averaging across the first three shots of each level ('poor': 2.63 ± .19; 'rich': 2.53 ± 14.), *F*(1, 31) = .13, *p* =	Although these 'poor' participants who could not borrow spent longer aiming their first shot of each level compared to the 'rich', they did not earn significantly more points on this shot ('poor': 3.12 ± .88; 'rich': 3.02 ± .69), *F*(1, 458) = 1.67, *p* = .197, $\eta^2(1)$ = .004, 95% CI [0.00, 0.02]. They also did not earn significantly more points on their second ('poor': 2.07 ± .72.; 'rich': 2.05 ± .49), *F*(1, 457) = .11, *p* = .735, $\eta^2(1)$ < .001, 95% CI [0.00, 0.01], or third shot of each level ('poor': 1.74 ± .78; 'rich': 1.72 ± .46), *F*(1, 453) = .08, *p* = .774, $\eta^2(1)$ < .001, 95% CI [0.00, 0.01]. Additional comparisons are not possible because 'poor' participants who could not borrow only had three shots per level. Further, there was no significant difference in points-per-shot between the 'rich' and the 'poor' when averaging across the first	Although 'poor' participants spent longer aiming their first shot of each level compared to the 'rich', they did not earn significantly more points on this shot ('poor': 3.14 ± .86; 'rich': 3.21 ± .71), *F*(1, 172) = .38, *p* = .538, $\eta^2(1)$ = .002, 95% CI [0.00, 0.04]. They also did not earn significantly more points on their second ('poor': 2.16 ± 1.01.; 'rich': 2.06 ± .52), *F*(1, 168) = .60, *p* = .439, $\eta^2(1)$ = .004, 95% CI [0.00, 0.04], or third shot of each level ('poor': 1.93 ± 1.43; 'rich': 1.89 ± .55), *F*(1, 145) = .04, *p* = .834, $\eta^2(1)$ < .001, 95% CI [0.00, 0.03]. Additional comparisons are not possible because 'poor' participants who could not borrow only had three shots per level. Further, there was no significant difference in points-per-shot between the 'rich' and the 'poor' when averaging across the

				.724, $\eta^2(1)$ = .004, 95% CI [0.00, 0.13]).	three shots of each level ('poor': 2.72 ± .05; 'rich': 2.62 ±.04), $F(1, 458)$ = 2.07, p = .151, $\eta^2(1)$ = .004, 95% CI [0.00, 0.02]).	first three shots of each level ('poor': 2.97 ± .09; 'rich': 2.88 ±.07), $F(1, 172)$ = .48, p = .490, $\eta^2(1)$ = .003, 95% CI [0.00, 0.04]).
Scarcity leads to greater focus.	The significantly longer aiming time of 'poor' participants who could not borrow emerged on the very first shot of the game ('poor': 8.19 ± .52; 'rich': 7.86 ± .52), $F(1, 66)$ = 6.38, p = .014, $\eta^2(1)$ = .088, 95% CI [0.00, 0.23].	The significantly longer aiming time of 'poor' participants who could not borrow emerged on the very first shot of the game, though not significantly so ('poor': 8.12 ± .63; 'rich': 8.06 ± .63), $F(1, 973)$ = 2.54, p = .111, $\eta^2(1)$ = .003, 95% CI [0.00, 0.13].	The significantly longer aiming time of 'poor' participants emerged on the very first shot of the game, though not significantly so ('poor': 8.46 ± .57; 'rich': 8.35 ± .69), $F(1, 172)$ = 1.30, p = .256, $\eta^2(1)$ = .007, 95% CI [0.00, 0.05].	On the very first shot of the game, 'poor' participants who could not borrow did not earn significantly more points than their 'rich' counterparts ('poor': 1.85 ± 1.63; 'rich': 2.38 ± 2.90), $F(1, 31)$ = 0.46, p = .503, $\eta^2(1)$ = .015, 95% CI [0.00, 0.17].	On the very first shot of the game, 'poor' participants who could not borrow did not earn significantly more points than their 'rich' counterparts ('poor': 3.10 ± 2.14; 'rich': 2.89 ± 2.22), $F(1, 458)$ = 1.09, p = .296, $\eta^2(1)$ =.002, 95% CI [0.00, 0.02].	On the very first shot of the game, 'poor' participants did not earn significantly more points than their 'rich' counterparts ('poor': 3.07 ± 2.17; 'rich': 3.00 ± 2.21), $F(1, 172)$ = .04, p = .843, $\eta^2(1)$ < .001, 95% CI [0.00, 0.02].
Scarcity leads to greater focus.	Among participants who could not borrow, the 'poor' earned significantly more points-per-shot (2.31 ± .60) than did the 'rich' (1.67 ± .37), $F(1, 31)$ = 11.91, p = .002, $\eta^2(1)$ = .278, 95% CI [0.05, 0.48].	Among participants who could not borrow, the 'poor' earned significantly more points-per-shot (2.42 ± .65) than did the 'rich' (1.91 ± .51), $F(1, 458)$ = 82.01, p < .001, $\eta^2(1)$ = .152, 95% CI [0.10, 0.21].	The 'poor' earned significantly more points-per-shot (2.73 ± .82) than did the 'rich' (2.20 ± .63), $F(1, 172)$ = 21.95, p < .001, $\eta^2(1)$ = .113, 95% CI [0.04, 0.21].	Among participants—both 'rich' and 'poor'—who could not borrow, later shots on each level were significantly less profitable compared to earlier shots, β = -.19, t = -14.85, p < .001 (-.22, -.17). 'Poor' participants employed significantly fewer shots on each level ('poor': 2.51 ± .45; 'rich': 5.67 ± 1.79), $F(1, 31)$ = 57.60, p < .001, $\eta^2(1)$ = .650, 95% CI [0.42, 0.76], such that they did not shoot the later, less-profitable shots that 'rich' participants shot, resulting in a higher points-per-shot average.	Among participants—both 'rich' and 'poor'—who could not borrow, later shots on each level were significantly less profitable compared to earlier shots, β = -22, t = -61.58, p < .001 (-.23, -.21). 'Poor' participants employed significantly fewer shots on each level ('poor': 2.59 ± .35; 'rich': 5.19 ± 1.64), $F(1, 458)$ = 619.34, p < .001, $\eta^2(1)$ = .575, 95% CI [0.52, 0.62], such that they did not shoot the later, less-profitable shots that 'rich' participants shot, resulting in a higher points-per-shot average.	Later shots on each level were significantly less profitable compared to earlier shots, β = -29, t = -34.28, p < .001 (-.30, -.27). 'Poor' participants employed significantly fewer shots on each level ('poor': 1.81 ± .51; 'rich': 3.83 ± 1.50), $F(1, 172)$ = 145.65, p < .001, $\eta^2(1)$ = .459, 95% CI [0.35, 0.54], such that they did not shoot the later, less-profitable shots that 'rich' participants shot, resulting in a higher points-per-shot average.
Scarcity leads to over-borrowi ng(or neglecting insurance option for conceptual	As a fraction of their budget, 'poor' participants borrowed significantly more shots (.24 ± .15) than the 'rich' (.02 ± .05), $F(1, 33)$ = 27.53, p < .001, $\eta^2(1)$ = .455, 95% CI [0.19, 0.62].	As a fraction of their budget, 'poor' participants borrowed significantly more shots (.20 ± .16) than the 'rich' (.03 ± .06), $F(1, 512)$ = 252.73, p < .001, $\eta^2(1)$ = .330, 95% CI [0.27, 0.39].	(not applicable - participants couldn't borrow in our conceptual replication study)	'Poor' participants were significantly more likely to face the decision of whether to borrow or not (45% of all levels) compared to the 'rich' (2% of all levels), □$^2(1)$ = 184.42, p < .001. When considering only cases in which participants could have decided to borrow, 'poor'	'Poor' participants were significantly more likely to face the decision of whether to borrow or not (48% of all levels) compared to the 'rich' (2% of all levels), □$^2(1)$ > 100, p < .001. When considering only cases in which	'Poor' participants did not purchase significantly fewer insurances ('poor': 5.24 ± 3.21; 'rich': 5.77 ± 3.49), $F(1, 172)$ = 1.08, p = .299, $\eta^2(1)$ = .006, 95% CI [0.00, 0.05].

replication study).				participants were if anything *more likely* to resist the temptation to borrow (31%) compared to 'rich' participants (0%), though this effect wasn’t significant, $\chi^2(1) = 3.79$, $p = .052$. This was despite the fact that the option to borrow was arguably more attractive for 'poor' participants, as they had significantly more targets left to clear, on average, after exhausting their budget (2.54 ± 1.322) than 'rich' participants did (.54 ± .637), $F(1, 33) = 29.15$, p < .001, $\eta^2(1) = .469$, 95% CI [0.21, 0.63].	participants could have decided to borrow, 'poor' participants were if anything *more likely* to resist the temptation to borrow (61%) compared to 'rich' participants (6%), $\chi^2(1) = 56.84$, $p < .001$. This was despite the fact that the option to borrow was arguably more attractive for 'poor' participants, as they had significantly more targets left to clear, on average, after exhausting their budget (1.92 ± 1.03) than 'rich' participants did (.37 ± .53), $F(1, 513) = 421.26$, $p < .001$, $\eta^2(1) = .451$, 95% CI [0.39, 0.50].	
Scarcity leads to over-borrowing/ neglecting insurance option.	'Rich' participants performed similarly whether they could not borrow (1.67 ± .37) or could (1.81 ± .53), while 'poor' participants fared significantly better when they could not borrow (2.31 ± .60) than when they could (1.77 ± .97) [scarcity×borrowing interaction: $F(1, 64) = 4.04$, $p = .049$, $\eta^2(1) = .059$, 95% CI [0.00, 0.19]].	'Rich' participants performed similarly whether they could not borrow (1.91 ± .51) or could (1.95 ± .57), while 'poor' participants fared better when they could not borrow (2.42 ± .65) than when they could (2.09 ± .74) [scarcity×borrowing interaction: $F(1, 971) = 19.93$, $p < .001$, $\eta^2(1) = .020$, 95% CI [0.01, 0.04]].	(not applicable - participants couldn’t borrow in our conceptual replication study)	Overall, across both 'rich' and 'poor' participants, performance was significantly negatively related to frequency of borrowing, $r(33) = -.688$, $p < .001$, as well as the relative amount of borrowing, $r(33) = -.730$, $p < .001$. Thus, borrowing was suboptimal for all participants, and 'poor' participants’ performance was particularly affected by borrowing because they faced the decision to borrow more often (see cell above).	Overall, across both 'rich' and 'poor' participants, performance was significantly negatively related to frequency of borrowing, $r(513) = -.665$, $p < .001$, as well as the relative amount of borrowing, $r(513) = -.648$, $p < .001$. Thus, borrowing was suboptimal for all participants, and 'poor' participants’ performance was particularly affected by borrowing because they faced the decision to borrow more often (see cell above).	While there is no significant difference in how likely the ‘poor’ (.59 ± .35) vs. the ‘rich’ (.61 ± .36) are to buy insurances with a positive expected value, $F(1, 172) = .17$, $p = .682$, $\eta^2(1) < .001$, 95% CI [0.00, 0.03], the ‘poor’ are significantly less likely to purchase insurances with a negative expected value ('poor': .25 ± .35; 'rich': .43 ± .46), $F(1, 172) = 8.24$, $p = .005$, $\eta^2(1) = .046$, 95% CI [0.00, 0.12].
When facing scarcity, greater focus on current demands predicts	On levels where 'poor' participants borrowed, the average amount of time spent aiming each shot in their paycheck was significantly positively	On levels where 'poor' participants borrowed, there was not a significant correlation between the average amount of time spent aiming each shot in	(not applicable - participants couldn’t borrow in our conceptual replication study)	This correlation is not statistically significant when investigating all observations from participants (both 'rich' and 'poor') who could borrow,		

neglect of future demands.	correlated with how many shots they subsequently borrowed, *r*(38) = .43, *p* = .008.	their paycheck and how many shots they subsequently borrowed, *r*(633) = -.034, *p* = .399.		*r*(585) = .03, *p* = .452, all observations of the 'poor' who could borrow, *r*(122) = .06, *p* = .505, or all levels on which participants borrowed, *r*(47) = .15, *p* = .329.

Note: Shah et al. (2019) is a large-scale replication of the original study of Shah et al. (2012). Our conceptual replication represents larger deviations from the original study setup, specifically we used insurance purchasing instead of borrowing as the secondary task.

Supplementary Note 2: Equivalence testing and power considerations

Equivalence testing is a statistical method used to assess whether the effects of two treatments or conditions are practically equivalent. It involves testing whether the difference between the two groups falls within a predefined range called the equivalence margin, which is based on the smallest effect size of interest (SESOI). This margin represents the maximum difference that would still be considered negligible for practical purposes. Unlike traditional hypothesis testing, which focuses on detecting significant differences, equivalence testing aims to demonstrate that any observed difference is smaller than the SESOI, thus showing that the treatments or conditions are practically equivalent.

For conducting equivalence testing, we generally followed the recommendations of Lakens et al. (2018). We used the two one-sided tests (TOST) procedure to test for equivalence, implemented in R using the TOSTER package (Lakens et al., 2018).

To calculate the SESOI, we followed the recommendations of Lakens et al. (2018) and calculated the smallest effect size that the original study (Shah et al., 2012) would have 33% power to detect. While we acknowledge the limitations of basing the SESOI on a statistical criterion, the highly artificial nature of the experimental design does not allow for a practical justification of it. Even though most analyses of Shah et al. (2012) only include a subset of the whole sample (i.e. participants who could not borrow), we decided to take the whole sample for estimating the SESOI to give us a conservative measure for whether we can claim statistical equivalence. This resulted in a SESOI of $d = .374$. We will use this as a benchmark for all the following equivalence tests. When applied to the measure of points-per-shot, his effect size corresponds to a mean difference of approximately 0.271 points-per-shot, given the pooled standard deviation of .724 points-per-shot observed in Shah et al. (2012). Sensitivity analyses exploring more liberal (e.g., ±0.5 d) and more conservative (e.g., ±0.2 d) SESOI values are provided below to evaluate the robustness of our conclusions across different thresholds of practical significance.

Performance on shots 1 to 3

Within our paper, we argue that there was no significant difference in the performance of ‘rich’ and ‘poor’ participants on each of the first three shots individually. Using equivalence testing, we set out to investigate whether indeed the performance of the ‘rich’ and the ‘poor’ are statistically equivalent, meaning that we can claim to find no meaningful differences between these two groups.

Equivalence tests on the original dataset of Shah et al. (2012) did not reveal statistical equivalence between the ‘rich’ and the ‘poor’ on either the average performance of the first three shots, $t(30.75) = 0.712$, $p = .241$, or on the first, $t(28.25) = -0.711$, $p = .241$, second, $t(30.52) = 0.248$, $p = .403$, or third shot, $t(28.96) = 0.150$, $p = .441$ individually. As both the condition factor within the ANOVA models and the equivalence tests were insignificant, this suggests that the study was underpowered to draw any conclusions regarding whether there were differences in performance between the ‘poor‘ and the ‘rich‘ on the first three shots of each level.

However, performing equivalence tests on the more highly-powered large-scale replication of Shah et al. (2019) revealed statistical equivalence between the ‘rich’ and the ‘poor’ on the average performance of the first three shots, $t(457.74) = 2.556$, $p = .005$, as well as on the first shot, $t(457.25) = 2.702$, $p = .004$, the second, $t(451.03) = 3.717$, $p < .001$, and third shot, $t(430.43) = 3.778$, $p < .001$ individually. This suggests that the observed differences in performance were smaller than the SESOI of d = 0.374, and thus can be considered practically negligible.

Similarly, performing equivalence tests on our conceptual replication study revealed statistical equivalence between the ‘rich’ and the ‘poor’ on the average performance of the first three shots, $t(165.43) = 1.779$, $p = .039$, as well as on the first, $t(170.6) = -1.851$, $p = .033$, second, $t(129.53) = 1.668$, $p = .049$, and third shot, $t(80.7) = 1.980$, $p = .026$ individually. This means that also in our conceptual replication study the performance of the

‘rich’ and the ‘poor’ were statistically equivalent on all three shots, meaning that there was no meaningful difference between the two groups. These findings indicate that any differences between groups in the conceptual replication are also smaller than our SESOI of *d* = 0.374 and therefore any differences observed can be considered not meaningful, given this criterion.

Buying insurance

Performing equivalence testing on the difference between the ‘poor’ and the ‘rich’ in buying insurances in our conceptual replication study, we find statistical equivalence in participants’ likelihood to buy ‘reasonable’ insurances with a positive expected value, $t(169.56) = -2.052$, $p = .021$. This implies that within our SESOI of d = 0.374, there was no meaningful difference between the ‘rich’ and the ‘poor’.

Power curve for hypothetical sets of effect sizes and sample sizes

To gain a better understanding of the power achieved over the three different studies reported on in our manuscript, we plotted a power curve for hypothetical sets of effect sizes and sample sizes (see Figure S1). Additional sensitivity analyses taking into account the distribution of participants over different conditions indicate that the original study[1] had 80% power to detect only large effect sizes of up to *d* = .619. Our replication study had 80% power to find effect sizes of up to *d* = .379. Crucially, the large-scale replication of Shah et al. (2019) had 80% power to detect very small effect sizes of up to *d* = .161.

Figure S1

Power curve for different effect sizes.

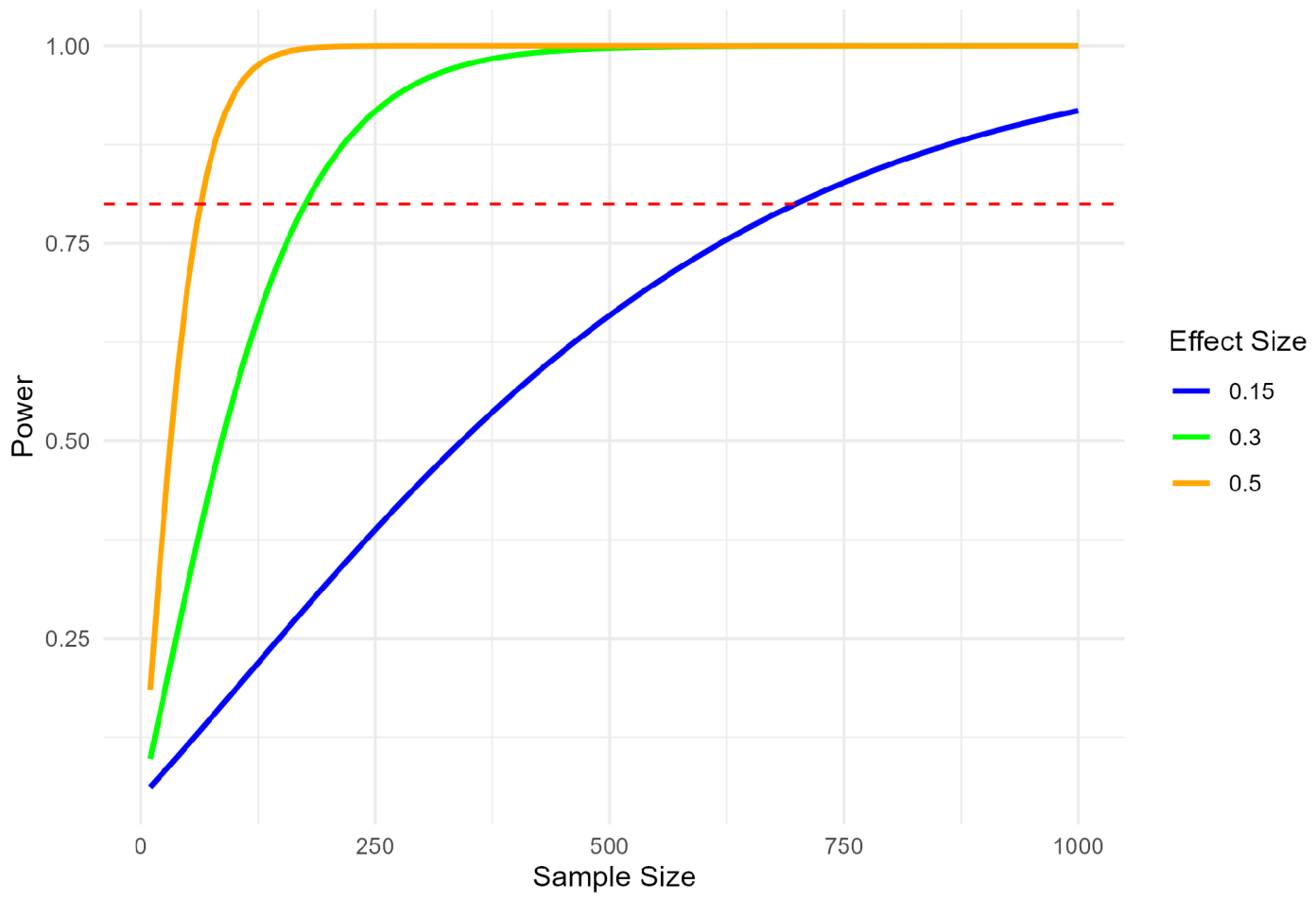

Finally, given that the SESOI we used was based on a statistical criterion and could thus be seen as somewhat arbitrary, we explored different SESOI values to test equivalence in performance on shot 3, which showed numerically the largest (non-significant) difference between conditions in the large-scale replication of Shah et al. (2019) with d = 0.064. As shown in Figure S2, even with an SESOI of 0.2 (a small effect size), the study is well-powered to detect equivalence. However, for much smaller SESOIs, such as d = .1, even the large-scale replication lacks sufficient power to demonstrate statistical equivalence (see Figure S2). In other words, if we would be interested in effects up to the size of .1, we could not conclude that there is actually no meaningful difference between the performance of the ‘poor’ and the ‘rich’. However, it is important to note that at these minimal effect sizes, it is unlikely that such small differences would carry meaningful implications for real-world decision-making (even when putting aside concerns of the ecological validity of the experiment more broadly).

Figure S2

Power curve for Two One-Sided Tests (TOST) equivalence test displaying different SESOIs

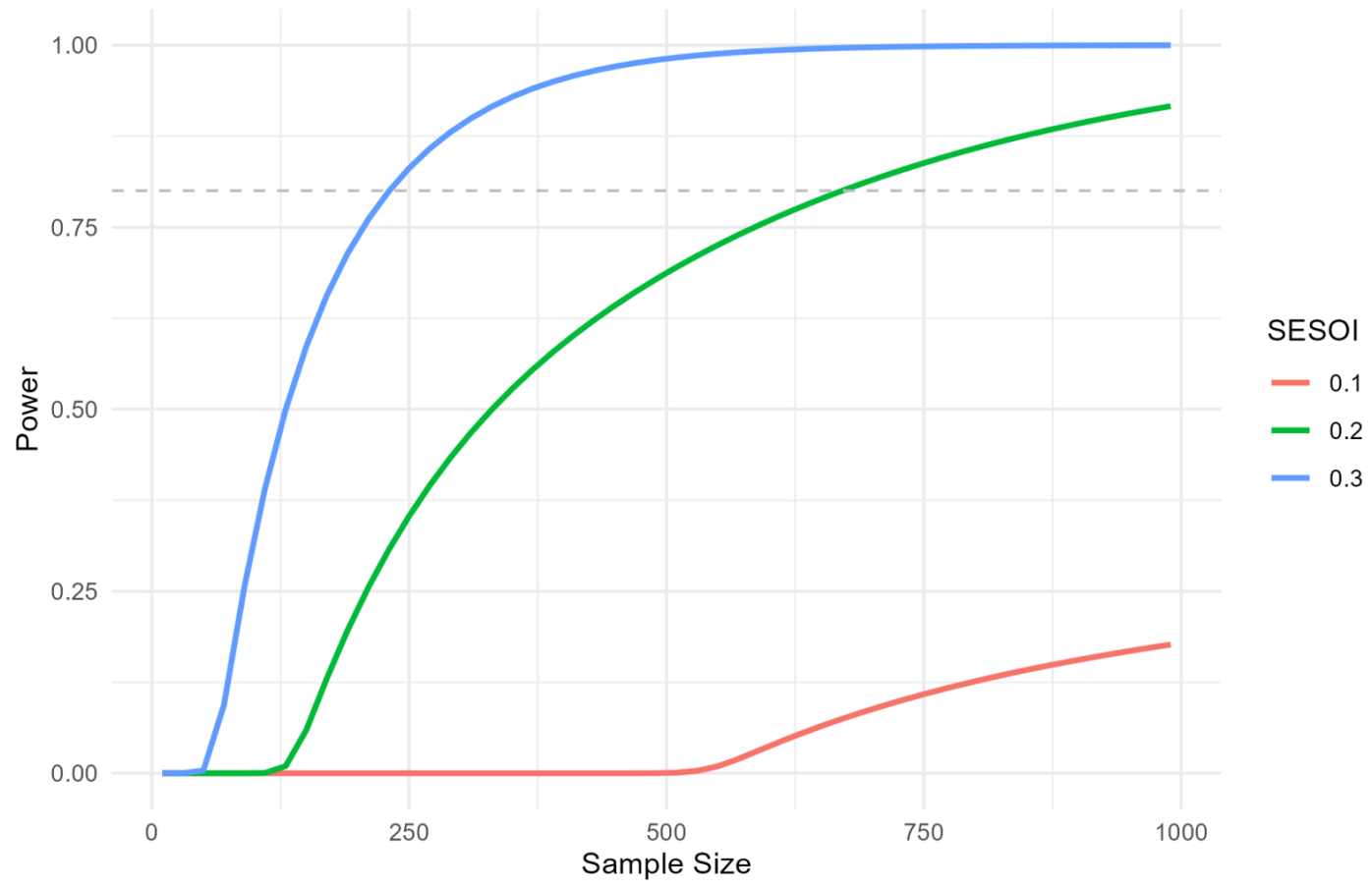

Supplementary Note 3: Sensitivity analyses

To assess the robustness of our findings, we conducted several sensitivity analyses based on different inclusion and exclusion criteria. The results are presented in Table S2. Column 1 replicates the main analyses reported in the manuscript, with no data excluded. Column 2 shows the results after excluding observations where participants took more than two standard deviations longer than the average to fire their shot (317 observations excluded). Column 3 applies a similar exclusion based on one standard deviation (710 observations excluded). In Column 4, we exclude the five participants who achieved perfect scores, meaning they earned 10 points on every level, as well as two participants who engaged excessively in borrowing.

Table S2. *Sensitivity analyses on Shah et al. (2012). All statistical tests were two-sided. No adjustments were made for multiple comparisons. Exact P values, test statistics (F or χ²), corresponding degrees of freedom, effect sizes, and 95% confidence intervals are provided in the table.*

Finding	No exclusions	Excluding very slow responses (>2SD)	Excluding slow responses (>1SD)	Excluding perfect scores and excessive borrowers
Performance on shots 1-3	Compared to the 'rich', 'poor' participants did not earn significantly more points on the first ('poor': 2.80 ± .99; 'rich': 2.91 ± .44), $F(1, 31) = .13$, $p = .718$, $\eta^2(1) = .004$, 95% CI [0.00, 0.13], second ('poor': 1.93 ± 1.67; 'rich': 1.77 ± .48), $F(1, 31) = .61$, $p = .440$, $\eta^2(1) = .019$, 95% CI [0.00, 0.19], or third shot of each level ('poor': 1.92 ± .74; 'rich': 1.73 ± .41), $F(1, 31) = .73$, $p = .401$, $\eta^2(1) = .024$, 95% CI	Compared to the 'rich', 'poor' participants did not earn significantly more points on the first ('poor': 2.80 ± .99; 'rich': 2.87 ± .44), $F(1, 31) = .06$, $p = .804$, $\eta^2(1) = .002$, 95% CI [0.00, 0.11], second ('poor': 1.92 ± .72; 'rich': 1.77 ± .49), $F(1, 31) = .46$, $p = .502$, $\eta^2(1) = .015$, 95% CI [0.00, 0.17], or third shot of each level ('poor': 1.97 ± .85; 'rich': 1.74 ± .42), $F(1, 31) = .81$, $p = .374$, $\eta^2(1) = .026$, 95% CI	Compared to the 'rich', 'poor' participants did not earn significantly more points on the first ('poor': 2.69 ± 1.02; 'rich': 2.85 ± .42), $F(1, 31) = .30$, $p = .589$, $\eta^2(1) = .010$, 95% CI [0.00, 0.16], second ('poor': 2.00 ± .81; 'rich': 1.77 ± .49), $F(1, 31) = .89$, $p = .352$, $\eta^2(1) = .028$, 95% CI [0.00, 0.20], or third shot of each level ('poor': 1.88 ± .87; 'rich': 1.74 ± .43), $F(1, 31) = .28$, $p = .603$, $\eta^2(1) = .009$, 95% CI [0.00, 0.16]	None of the exclusions were in the no borrow condition.

	[0.00, 0.20] within the no borrow condition.	[0.00, 0.20] within the no borrow condition.	within the no borrow condition.	
Likelihood of facing borrowing decision	'Poor' participants were significantly more likely to face the decision of whether to borrow or not (45% of all levels) compared to the 'rich' (2% of all levels), $\chi^2(1) = 184.42$, $p < .001$.	No changes, since only individual shots have been excluded.	No changes, since only individual shots have been excluded.	'Poor' participants were significantly more likely to face the decision of whether to borrow or not (41% of all levels) compared to the 'rich' (2% of all levels), $\chi^2(1) = 158.18$, $p < .001$.

Supplementary Note 4: Additional preregistered analyses for our conceptual replication study

Hypotheses 4 and 5. In addition to the hypotheses reported in the manuscript, we preregistered two further hypotheses (H4 and H5) in the “Other” section of the preregistration. In H4, we predicted that time preferences and risk preferences would mediate the relationship between the scarcity manipulation and insurance purchasing. However, there are several reasons why testing this mediation is not warranted. First, we do not observe a main effect of the scarcity manipulation on insurance purchasing ($t(172) = 1.041$, $p = .150$). Second, we find no significant differences between participants in the ‘poor’ versus ‘rich’ conditions with respect to time preferences ($t(172) = 0.510$, $p = .306$) or risk preferences ($t(172) = -1.132$, $p = .130$).

With respect to H5, we employed two different scarcity manipulations: one involving income shocks and one without income shocks (see Materials for details). We predicted that scarcity induced via income shocks would produce stronger behavioral effects than scarcity manipulations based solely on high versus low income levels. Specifically, we hypothesized an interaction between the type of scarcity manipulation (income shock vs. no income shock) and condition (scarce vs. non-scarce) across our three main hypotheses (H1–H3). However, similar to H4, we do not observe differences attributable to the income shock manipulation in key behavioral outcomes (e.g., points per shot or time spent aiming; all *p*s > .157). Consequently, conducting mediation analyses is not warranted.

Supplementary Note 5: Re-analyses of additional studies

Introduction

In our manuscript, we re-analyze data from Study 2 of the seminal paper by Shah et al. (2012), which is one of five studies reported in this publication. All five studies aimed to demonstrate the cognitive mechanisms of greater focus and attentional neglect. In our manuscript, we focus on Study 2 because Shah et al. (2012) identify it—alongside the now non-replicable Study 1 (see Shah et al., 2019; Camerer et al., 2018)—as foundational to their argument about the cognitive mechanisms purportedly driving the behavioral effects observed across all five studies. While Studies 3 through 5 are presented as additional support, the authors acknowledge that these do not provide direct evidence for the proposed cognitive mechanism. However, as we explain below, the same underlying logic that we applied in our re-analysis of Study 2 should, in principle, extend to Studies 3 through 5. That is, the behavioral effects of better performance, but also increased borrowing behavior of the 'poor' can be attributed to the game structure rather than to the cognitive mechanisms of greater focus and attentional neglect. In what follows, we briefly describe the game structures of Study 2 (Angry blueberries) and Studies 3 to 5 (Family Feud), highlight key similarities and differences, and then proceed to re-analyze the data for some of the latter studies in both Shah et al.'s original publication (2012), as well as their large-scale replication study (Shah et al., 2019).

Game structures of each study

'Angry blueberries' (Study 2)

In Study 2 of the original Shah et al. (2012) paper, participants played a custom video game called Angry Blueberries, in which they fired blueberries from a slingshot at waffles scattered across each level. Each level contained seven waffles, and participants earned one point per waffle hit, with an additional three-point bonus for clearing the entire level—yielding a maximum of 10 points per level. Participants loaded blueberries into the

slingshot via mouse click and could aim and adjust the velocity of their shots by dragging the projectile before release. Importantly, a blueberry was only subtracted from the participant's total budget once it was fired; unfired blueberries remained available for future rounds.

Participants were randomly assigned to either a 'poor' condition, with 30 total blueberries (3 per round), or a 'rich' condition, with 150 blueberries (15 per round). In addition, they were assigned to either a no-borrow condition or a borrowing condition with interest (R = 2), where each additional shot beyond the round's budget cost two blueberries from the overall budget. This means that participants could only borrow if they exhausted their budget and still had targets to clear on a level. Participants could also bank unused blueberries by exiting a round early. There were no time constraints. Participants were continuously shown their score, round-level usage, and remaining budget. Gameplay continued until the total blueberry allocation was exhausted.

'Family Feud' (Studies 3 to 5)

In Studies 3 to 5 of Shah et al. (2012), participants played a modified version of Family Feud, where they attempted to guess the five most popular responses to various survey questions (e.g., "Name things you take on a picnic"), previously answered by a panel of 100 people. Participants earned one point per correct guess, with a maximum of five points per round. Across studies, participants were categorized as 'poor' or 'rich,' with 'poor' participants receiving 300 seconds of total time (15 seconds per level) and 'rich' participants receiving 1000 seconds (50 seconds per level). In all studies, participants could bank unused time by exiting a round early. The banked time was reallocated to their remaining total budget, and this option became available after they had used 40% of their budget on each level (6 seconds for 'poor', 20 for 'rich' on each level).

The borrowing conditions varied across Studies 3 to 5. In Study 3, participants were assigned to one of three borrowing conditions: no borrowing, borrowing with no interest (R = 1; meaning no interest is being charged), or borrowing with interest (R = 2; essentially, 100% interest). In Study 4, participants either could not borrow or could borrow with R = 2.

Additionally, in this study, budget sizes on each level were dynamic and determined by averaging the remaining time over the remaining number of rounds, which introduced a shrinking-budget mechanism as debt accumulated. Study 5 followed the same basic structure as Study 3 but introduced a single borrowing condition at a higher cost (R = 3; essentially, 200% interest) and manipulated whether participants could preview the next round's question. Across all studies, participants played until their time budget was exhausted or until they completed 20 rounds (in Study 4), with scores and remaining time displayed throughout.

Comparing game structures

There are several structural similarities between the Angry Blueberries and Family Feud game paradigms. First, in both tasks, participants were given a limited budget (of shots or seconds) with which to complete a specific objective, either clearing seven targets or generating five correct guesses. A difference, however, is that in Angry Blueberries, clearing all targets awarded a three-point bonus, whereas no such bonus was given for identifying five correct answers in Family Feud.

Second, both games allowed participants to transition between levels and conserve unused resources for future rounds. However, the mechanics differed. In Angry Blueberries, participants could advance at any time. In Family Feud, progression was only possible after 40 percent of the round's time budget had elapsed. This imposed a minimum time expenditure of 6 seconds for "poor" participants and 20 seconds for "rich" participants. As we will discuss later, this structural constraint in the Family Feud paradigm may have prevented "rich" participants from implementing the optimal strategy, even if they understood it.

Third, in both study setups, resource scarcity was operationalized by assigning smaller budgets to "poor" participants compared to their "rich" counterparts, either in terms of shots in Angry Blueberries or time in Family Feud.

An additional difference is that in Family Feud, participants in borrowing conditions could spend their entire time budget on a single trial without making any guesses, simply by waiting. Some participants indeed engaged in this behavior. In contrast, the Angry Blueberries task required participants to actively engage by firing shots; otherwise, they could not progress.

Despite these differences, the two paradigms share key structural features, which allow us to apply some of the logic of our reanalysis of Study 2 (Angry Blueberries) to Studies 3 to 5 (Family Feud).

Re-analyses of additional studies

As described above, our focus was on re-analyzing the data from Study 2 (Shah et al., 2012; Shah et al., 2019) because it is most central to their tunneling argument. However, in what follows, we additionally report re-analyses for some of their additional studies (note that the study numbers in Shah et al., 2012 parallel the study numbers in Shah et al., 2019, i.e., Study 2 in Shah et al., 2019 is a replication of Study 2 in Shah et al., 2012, and so forth). Upon inspecting the datasets for Studies 3 to 5, we found that the datasets of Study 3 in Shah et al. (2012), as well as in Shah et al. (2019) lacked critical information required for our re-analyses. Specifically, we were unable to identify the exact time points at which correct guesses occurred (note that the variables in the uploaded data were not clearly labeled, and we had to infer variable identity from the data structure and content). Moreover, the design of Study 5 (in both Shah et al., 2012 and Shah et al., 2019) was such that it did not include a "no borrow" condition. Thus, results regarding the "greater focus" hypothesis cannot be directly compared to those of Study 2, as the authors used the "no borrow" condition to investigate the "greater focus" claims in Study 2. Consequently, the re-analyses presented below focus on Study 4 of the large-scale replication study (Shah et al., 2019). However, we additionally report results of our re-analyses for Study 4 in Shah et al., 2012, and Study 5 in both Shah et al., 2012 and Shah et al., 2019 in Table S3. All analysis scripts can be found in our OSF repository: osf.io/9m7kp.

Results of the re-analysis (Study 4; Shah et al., 2019)

Greater focus: diminishing returns and performance on first 15 seconds

To reiterate the general logic we applied to Study 2 (discussed in the manuscript): We showed that even though the 'poor' earned more points per shot throughout the game, this increase in performance was actually not due to better aiming resulting from greater focus: When looking at the first 3 shots of each level, there was no meaningful difference between the 'rich' and the 'poor'. Further, we showed that the increase in performance of the 'poor' can instead be attributed to the fact that the shots on each level show diminishing returns for all participants, with the first shots being more efficient than later shots on each level. Since the 'poor' were forced to spend their limited budget on only the first three shots (and not on the later, less efficient shots 4 to 15), they were more efficient on average throughout the game.

We can observe a similar pattern in the Family Feud setup: In Study 4, in the no borrow condition, we find that 'poor' participants are more efficient with their time: over the whole game, they make more correct guesses per second compared to the 'rich', $t(417.53) = 18.661$, $p < .001$. However, as in Study 2, we can show that this is not due to higher accuracy stemming from greater focus by zooming in on the first 15 seconds of each level, as both the 'rich' and the 'poor' could use these first 15 seconds. In fact, we find no significant difference in performance between the 'rich' and the 'poor' on these first 15 seconds of each level, $t(410.35) = -1.735$, $p = .084$ (in other datasets of Studies 4 and 5, these tests were also not significant, all ps > .195, see Table S3). Just as in Study 2, it seems that it was the diminishing returns of guesses in later seconds on each level that made the 'poor' more efficient: Guesses during earlier seconds on a level are more 'efficient', that is participants make more correct guesses during earlier seconds of each level compared to later seconds (see Figure S3), likely because easier-to-generate answers to the trivia questions have not yet been guessed. Since the 'poor' were forced to move to the next level after 15 seconds, they are restricted to making guesses during these "more

efficient" earlier seconds of each level (i.e., seconds 1 to 15), and do not "waste" their budget on the less efficient later seconds (i.e. seconds 16 to 50). In contrast, the 'rich' were able to continue guessing on these less efficient later seconds, rendering them less efficient overall.

Figure S3. Diminishing returns of seconds within each level (in Study 4, no borrow condition)

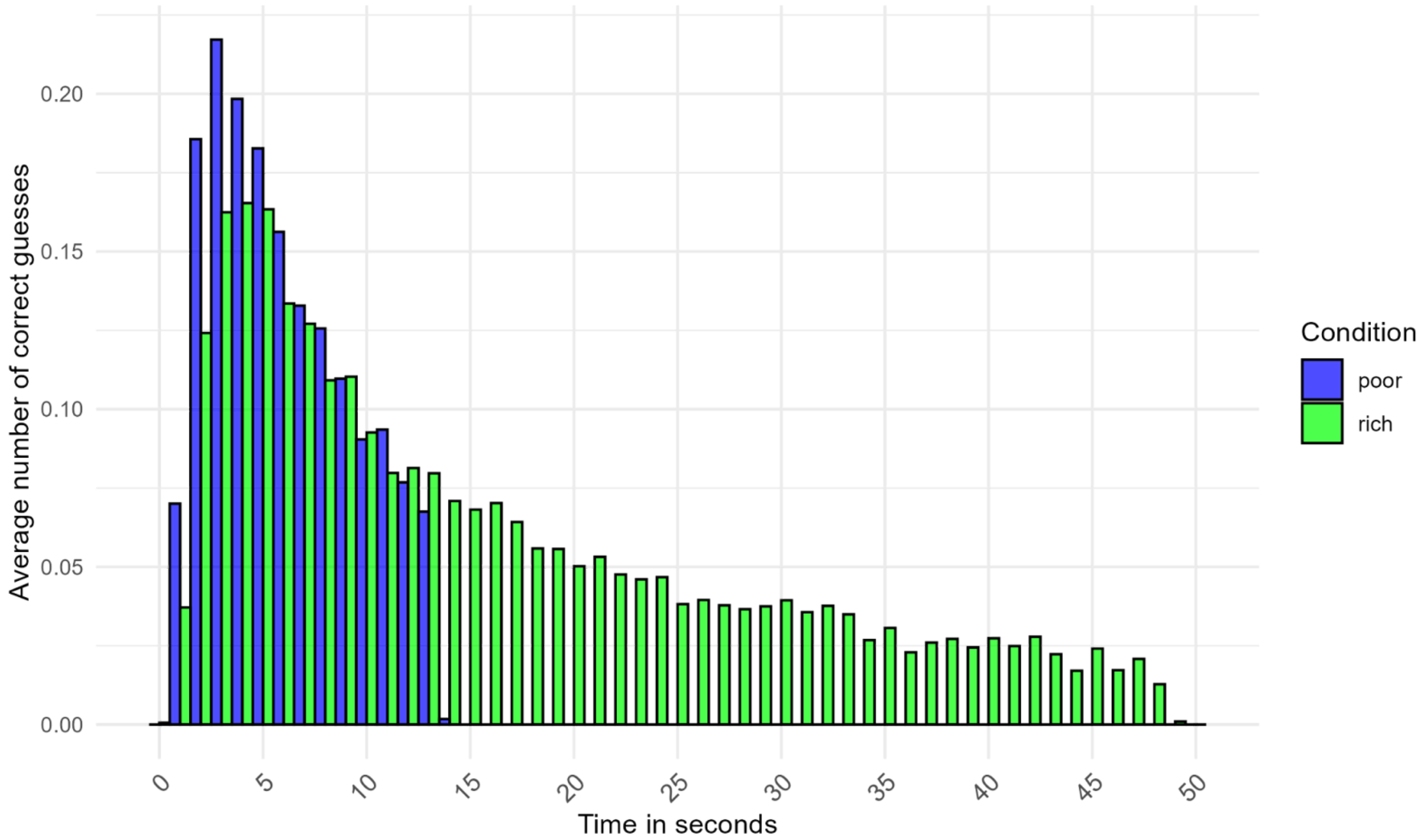

In fact, when examining how much time participants spent per level, we found that most participants in the no-borrow conditions used their entire available time budget in each round (Figure B2). This indicates that the 'rich' indeed spent a substantial portion of their budget on the later, less efficient seconds. It also suggests that both the 'rich' and the 'poor' generally adopted similar strategies, consistently using all the time available to them. As explained previously, given the task structure, this led to better performance among the 'poor', who were by design forced to stick to the earlier, more efficient seconds. Based on our data, it looks like the optimal strategy would have been to use only the first 10 seconds of each level before moving on, due to the diminishing returns of additional seconds on each level (see Figure S3). However, participants did not follow this strategy (see Figure S4). Crucially, even if they had recognized it, the 'rich' participants could not have implemented it:

all participants were only allowed to progress to the next level after using at least 40 percent of their time budget on each level. For the ‘poor’, this meant that the option to progress to the next level became available after 6 seconds. For the ‘rich’, however, it only became available after 20 seconds. Due to diminishing returns, this design feature meant that the ‘rich’ were structurally prevented from employing the optimal game strategy.

Figure S4. Average time participants in the no borrow conditions spent on each level.

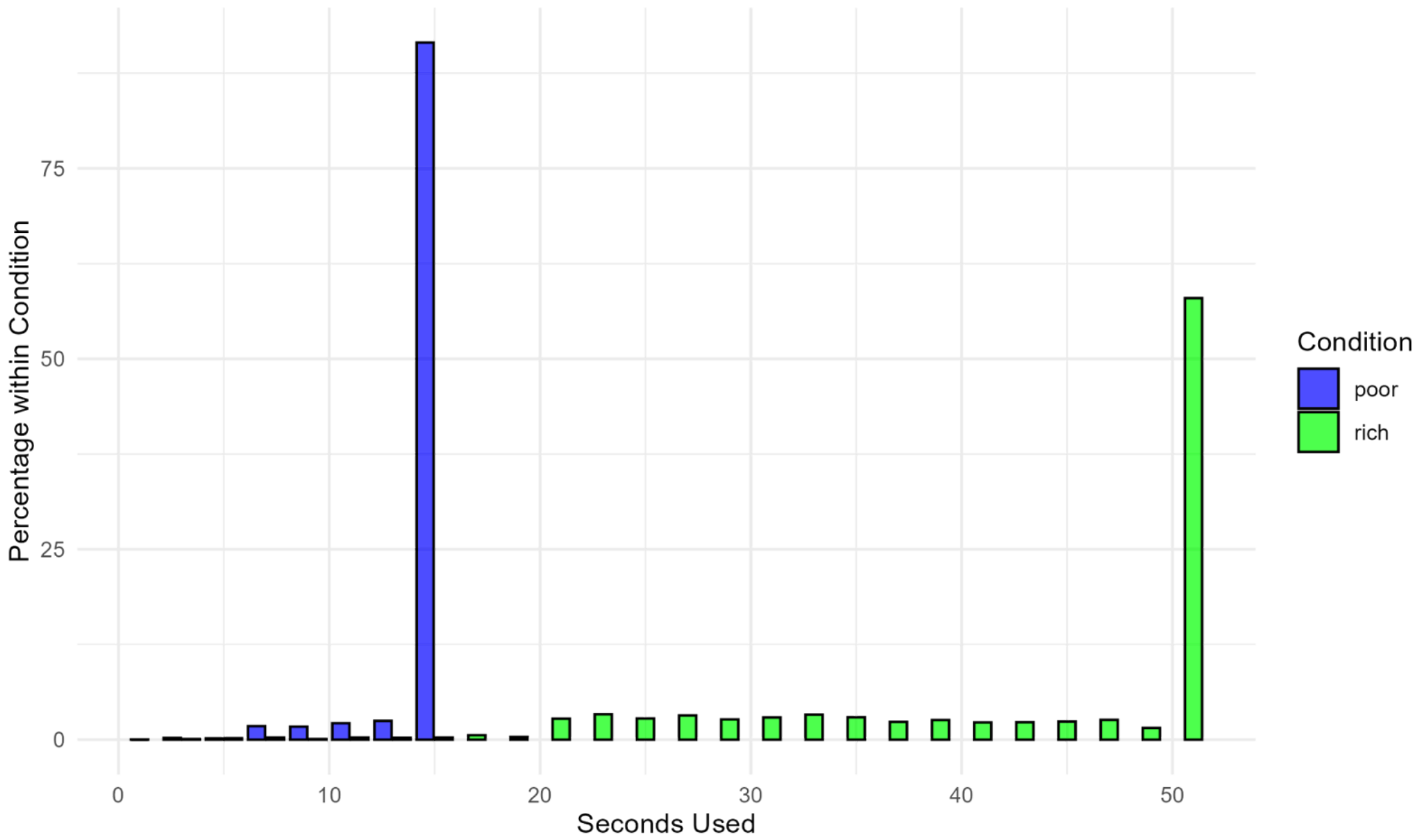

Attentional neglect: borrowing behavior

In Study 2, we demonstrated that the performance decline observed when participants were allowed to borrow was not due to attentional neglect but rather due to differences in the base rate at which participants encountered opportunities to borrow (i.e., borrowing was counter-productive for all participants but poor participants received this option much more than rich participants). In the Angry Blueberries setup, borrowing was only possible when participants had exhausted their level-specific budget but still had targets to clear. ‘Rich’ participants almost never faced this situation, whereas ‘poor’ participants encountered it in nearly half of their trials. Applying this logic to the borrow condition in Study 4 again revealed a clear and statistically significant difference between groups: participants

in the ‘poor’ condition reached a state where borrowing would have been possible in 58.4% of trials, compared to 28.0% in the ‘rich’ condition, $\chi^2(1) = 554.21$, $p < .001$. When zooming in on those levels on which participants could have borrowed, there was no significant difference between the ‘poor’ (82.21%) and the ‘rich’ (81.56%) in their propensity to borrow, $\chi^2(1) = 0.116$, $p = .734$. Thus, just as in Study 2, there was no evidence in Study 4 that the ‘poor’ were less attentive to the costs of borrowing than the ‘rich’; borrowing was counter-productive for all participants but the ‘poor’ received the option to borrow more often than the ‘rich’.

Moreover, it is not only the difference in base rates that contributes to borrowing being more detrimental to the performance of the ‘poor’ compared to the ‘rich,’ but also the diminishing returns of guesses in later seconds discussed above. Having the option to borrow meant that the ‘poor’ who could borrow in Study 4 were allowed to also make guesses during the less efficient later seconds of a level. As such, borrowing meant not only that participants had to pay interest rates on their seconds borrowed from future rounds, but also that they made more guesses during less-efficient later seconds of a level.

Additional results from Studies 3 and 5

The fact that borrowing in the Family Feud paradigm had two kinds of costs (incurring interest rates and making guesses during the less efficient later seconds of a level) is nicely illustrated in Study 3, which included a condition allowing participants to borrow without interest. In this condition, both ‘poor’ and ‘rich’ participants could continue playing each level for as long as they wanted without any penalty. The only difference was that the ‘poor’ received a borrowing warning after 15 seconds, while the ‘rich’ received it after 50 seconds. This may have served as a subtle nudge to move on. Though we were not able to re-analyze the data from this study, Shah et al. (2012), report that even in the absence of interest rates, this condition negatively affected the performance of the ‘poor’ but not of the ‘rich’. This finding is consistent with the idea of diminishing returns of guesses during later seconds of a level. In the no-borrow condition of the Family Feud setup, the ‘poor’ were constrained to a

strategy that happened to be beneficial, focusing on the first 15 seconds of each level. When allowed to play longer, their performance declined, due to the lower value of making guesses during later less efficient seconds of levels. In borrowing conditions with added interest costs, the decline in performance likely reflects both the effects of diminishing returns and the additional financial cost of borrowing. Again, this suggests that borrowing was a suboptimal strategy for all participants, but that the 'poor' were disproportionately more likely to engage in it due to the higher base rate at which they encounter borrowing opportunities.

In order to assess the robustness of our results within the Family Feud setup, we also replicated the analyses presented above on Study 4 using the data of Study 5, which contained two distinct borrow conditions. The observed pattern proves to be highly robust across these different experimental manipulations. Across all available datasets and conditions, we find no significant differences between the ‘rich’ and the ‘poor’ in performance in terms of points-per-second during the first 15 seconds of each level (see Table S3). Moreover, participants in the ‘poor’ conditions are consistently more likely than those in the ‘rich’ conditions to encounter the decision of whether to borrow, though ‘poor’ participants are no more likely to borrow than ‘rich’ participants when controlling for these base rates (see Table S3).

Table S3. Overview of results

Result	Study 4 - 2019 (no borrow)	Study 4 – 2019 (borrow)	Study 5 - 2019 (borrow, no preview)	Study 5 - 2019 (borrow, preview)	Study 5 - 2012 (borrow, no preview)	Study 5 -2012 (borrow, preview)
When looking at the first 15 seconds of each level, there is no significant difference in performance between the 'rich' and 'poor'.	$t(410.35) = -1.735$, $p = .084$.	$t(365.42) = 1.290$, $p = .198$	$t(467.9) = 0.883$, $p = .378$	$t(262.05) = 1.296$, $p = .196$	$t(67.90) = 1.470$, $p = .146$	$t(64.368) = 1.187$, $p = .240$
The 'poor' face the decision of whether to borrow significantly more often than the 'rich'.	Levels on which participants could borrow: 'poor': 90.385% 'rich': 58.493% $\chi^2(1) = 1088.2$, $p < .001$	Levels on which participants could borrow: 'poor': 58.381% 'rich': 27.962% $\chi^2(1) = 554.21$, $p < .001$	Levels on which participants could borrow: 'poor': 63.272% 'rich': 19.486% $\chi^2(1) = 1579.5$, $p < .001$	Levels on which participants could borrow: 'poor': 62.680% 'rich': 17.009% $\chi^2(1) = 1699.8$, $p < .001$	Levels on which participants could borrow: 'poor': 58.434% 'rich': 17.671% $\chi^2(1) = 219.52$, $p < .001$	Levels on which participants could borrow: 'poor': 49.020% 'rich': 10.795% $\chi^2(1) = 261.61$, $p < .001$
When zooming in on those levels on which participants could have borrowed, there was no significant difference between the 'poor' and the 'rich' in their propensity to borrow.	NA	There was no significant difference between the 'poor' (82.21%) and the 'rich' (81.56%) in their propensity to borrow on these levels, $\chi^2(1) = 0.116$, $p = .734$.	There was no significant difference between the 'poor' (83.79%) and the 'rich' (84.40%) in their propensity to borrow on these levels, $\chi^2(1) = 0.147$, $p = .701$.	There was no significant difference between the 'poor' (83.66%) and the 'rich' (85.04%) in their propensity to borrow on these levels, $\chi^2(1) = 0.758$, $p = .384$.	There was no significant difference between the 'poor' (85.57%) and the 'rich' (78.03%) in their propensity to borrow on these levels, $\chi^2(1) = 3.173$, $p = .075$.	There was no significant difference between the 'poor' (76.00%) and the 'rich' (80.00%) in their propensity to borrow on these levels, $\chi^2(1) = 0.465$, $p = .495$.

Discussion

We were able to apply the logic of our re-analyses for Study 2 to Studies 4 and 5, yielding a similar pattern of findings. The re-analyses of Studies 4 and 5 suggest that improved performance among the 'poor' observed in those studies was not the result of greater focus, nor was increased borrowing driven by attentional neglect, contrary to the tunneling hypothesis. Rather, and consistent with the findings of our re-analyses for Study 2 (presented in the main text), the evidence suggests that it was the structure of the Family Feud task itself in each of these studies that compelled participants in the 'poor' conditions to make guesses during the more efficient early seconds of each level, when it was easier to make correct guesses (contra the interpretation of increased focus). At the same time, the design also exposed the ‘poor’ to counter-productive borrowing opportunities more frequently than ‘rich’ participants received these opportunities, even though the “poor” were no more likely to borrow when presented these opportunities (contra the attentional neglect interpretation).

Supplementary Note 6: Tests for Normality of Residuals and Homogeneity of Variance

Table S4. Results of Tests for normality of residuals (Q-Q plot of residuals) and homogeneity of variance (Brown–Forsythe test) for main dependent variables across different datasets.

<table>
<tr><th></th><th>Shah et al., 2012</th><th>Shah et al., 2019</th><th>tho Pesch et al., 2026</th></tr>
<tr><td>Time per shot first shot of each level</td><td>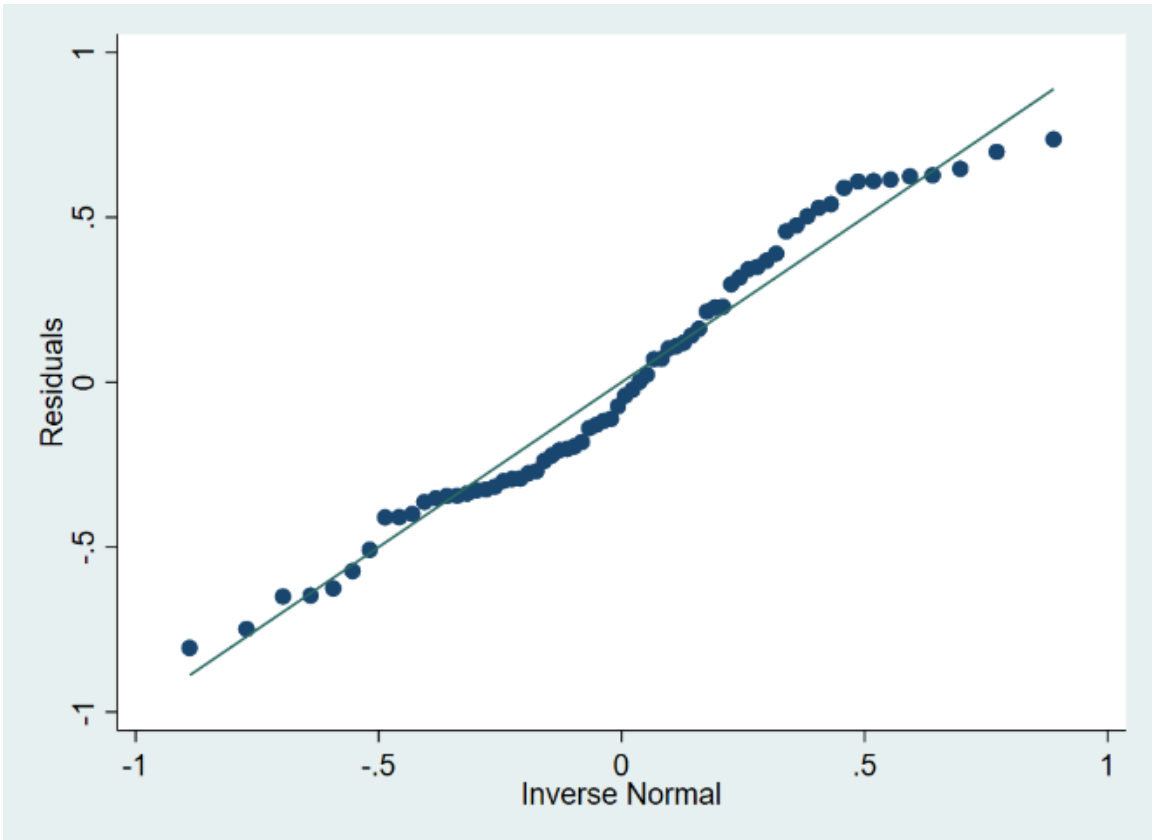
F(1, 66) = 0.51, p = .477</td><td>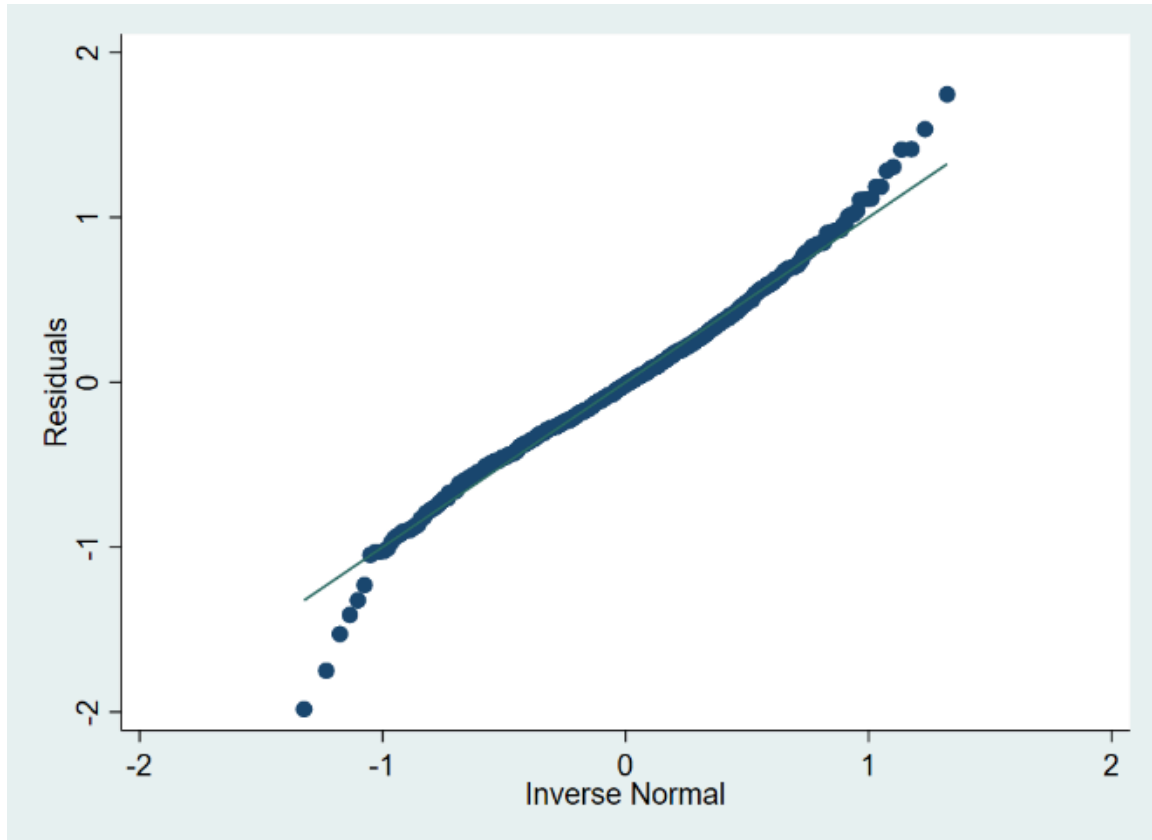
F(1, 973) = 8.32, p = .004</td><td>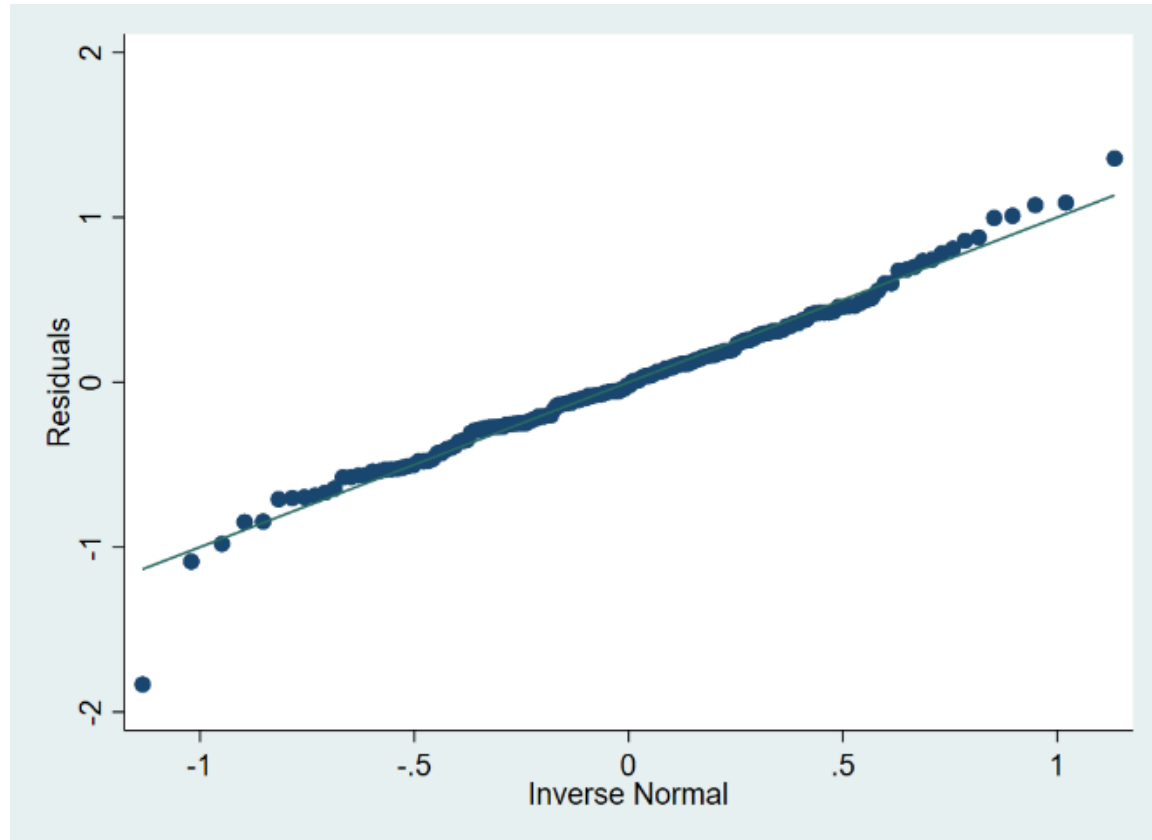
F(1, 172) = 0.04, p = .848</td></tr>
<tr><td>Points-per-shot across whole game</td><td>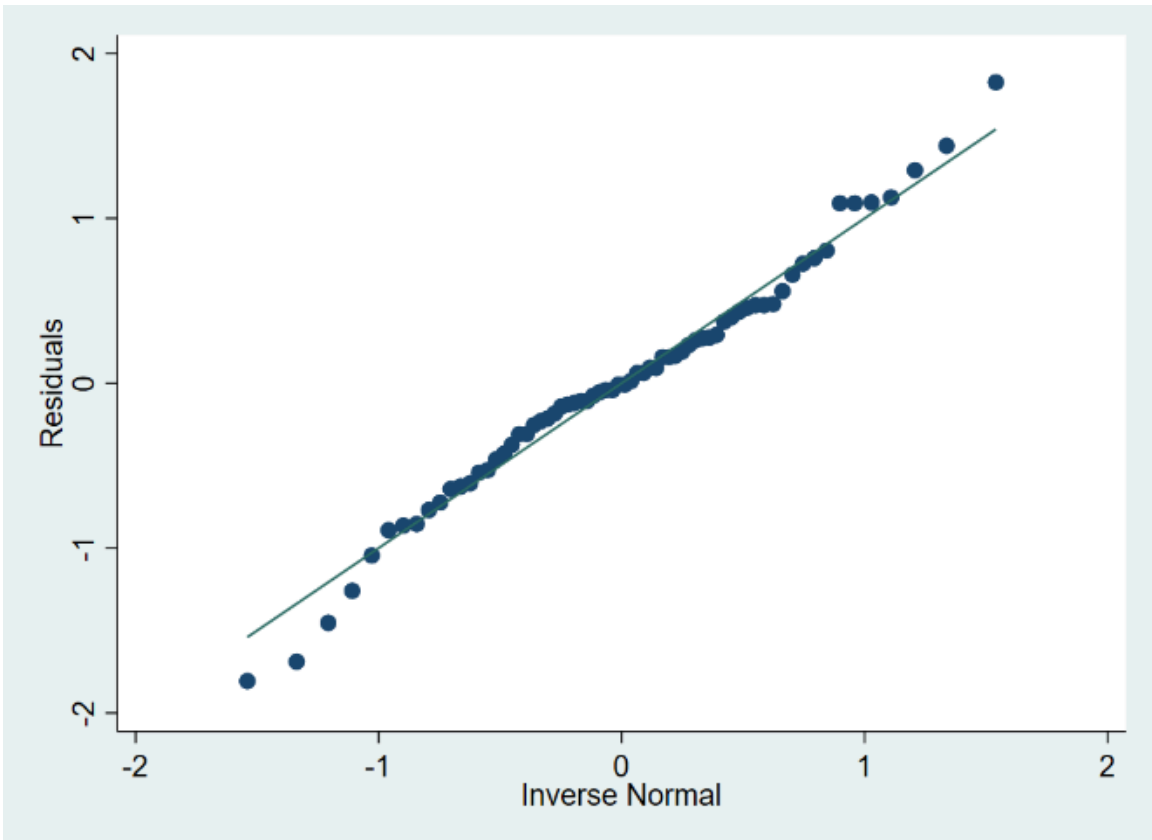</td><td>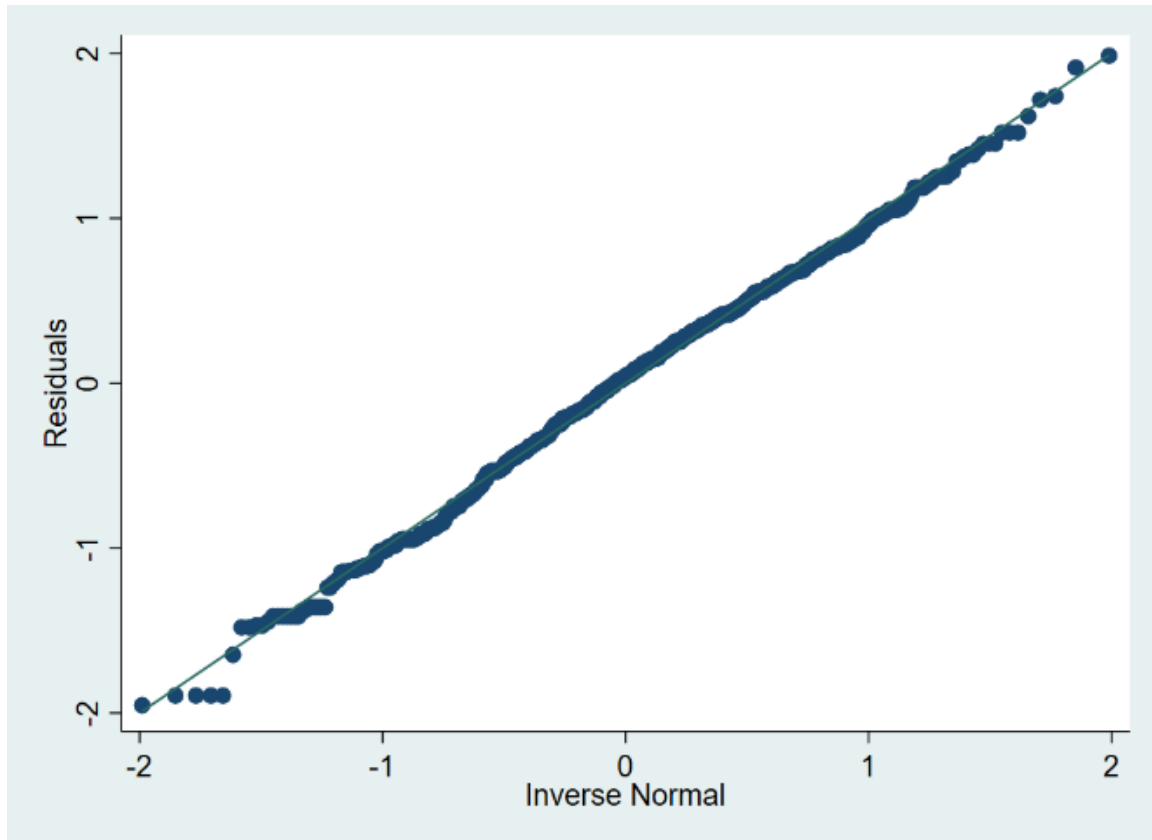</td><td>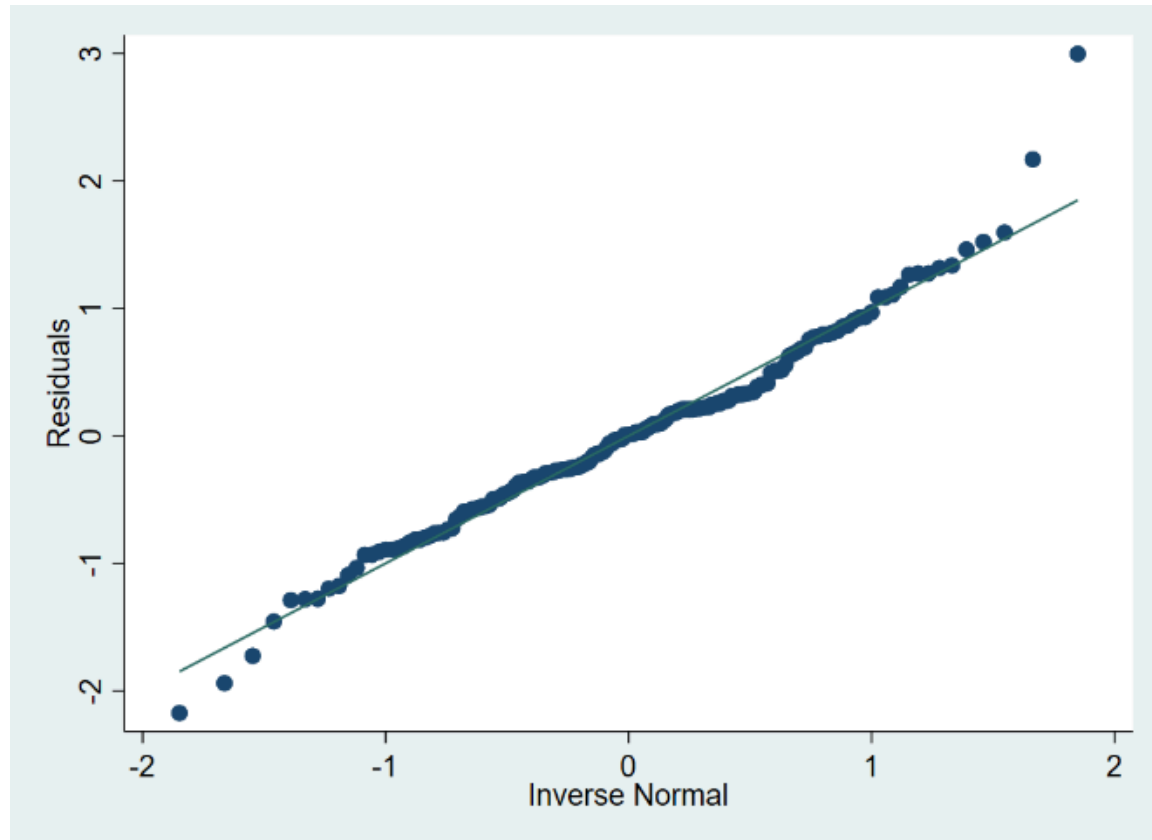</td></tr>
</table>

	F(1, 66) = 6.69, *p* = .012	*F*(1, 973) = 33.46, *p* < .001	*F*(1, 172) = 2.20, *p* = .140
Relative amount of borrowing/ Insurances bought	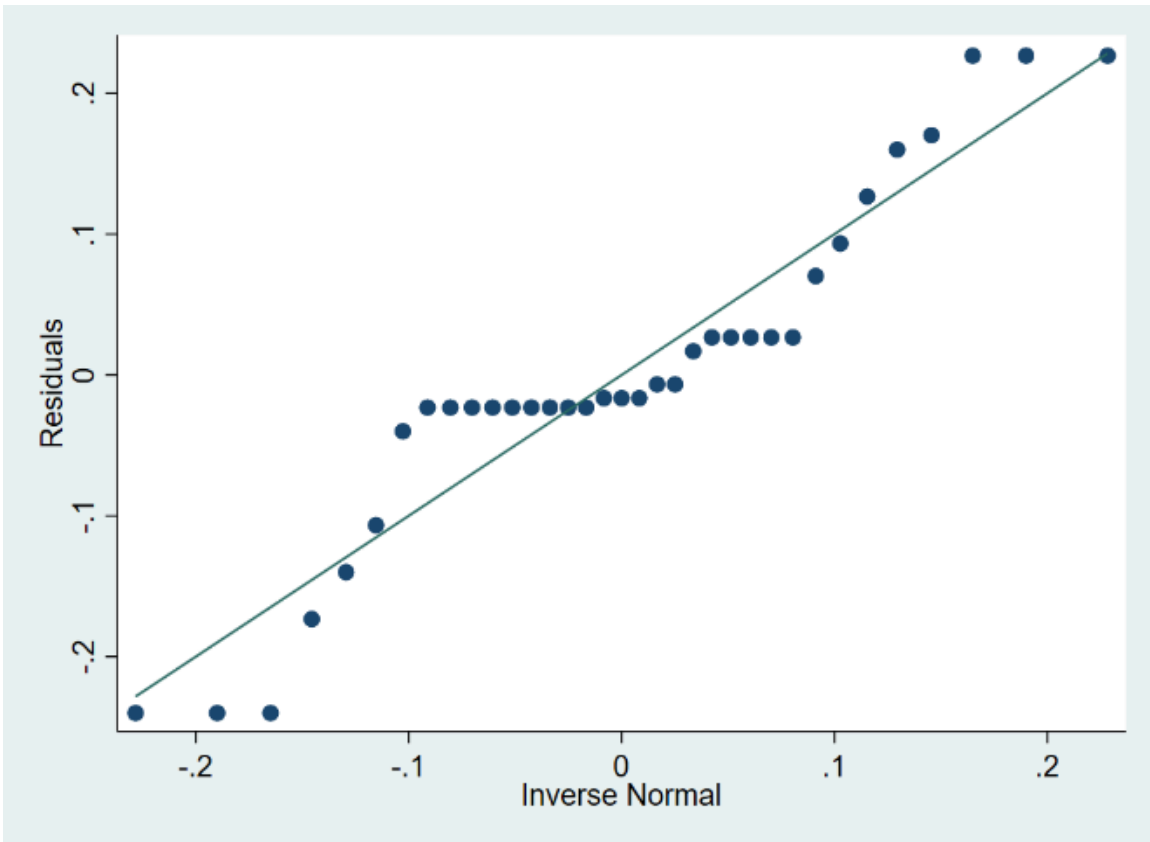<br><br>*F*(1, 33) = 10.99, *p* = .002	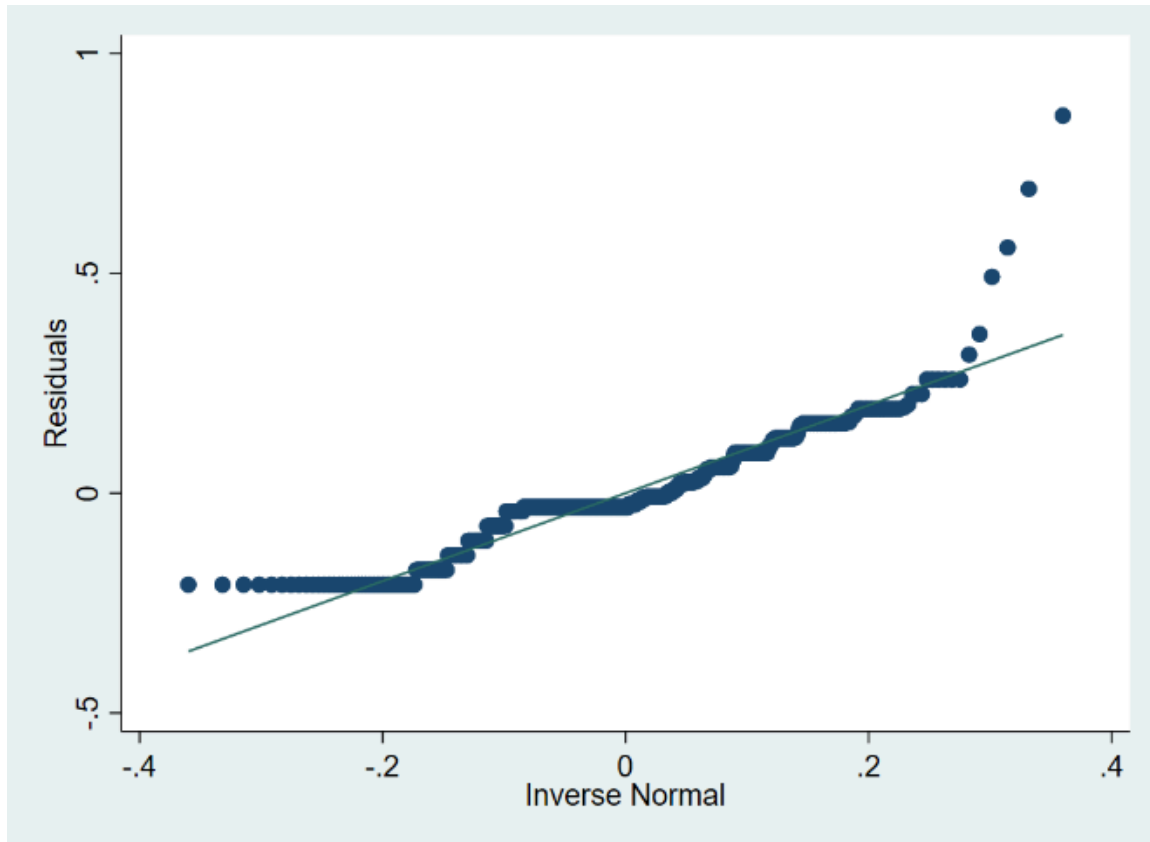<br><br>*F*(1, 512) = 165.65, *p* < .001	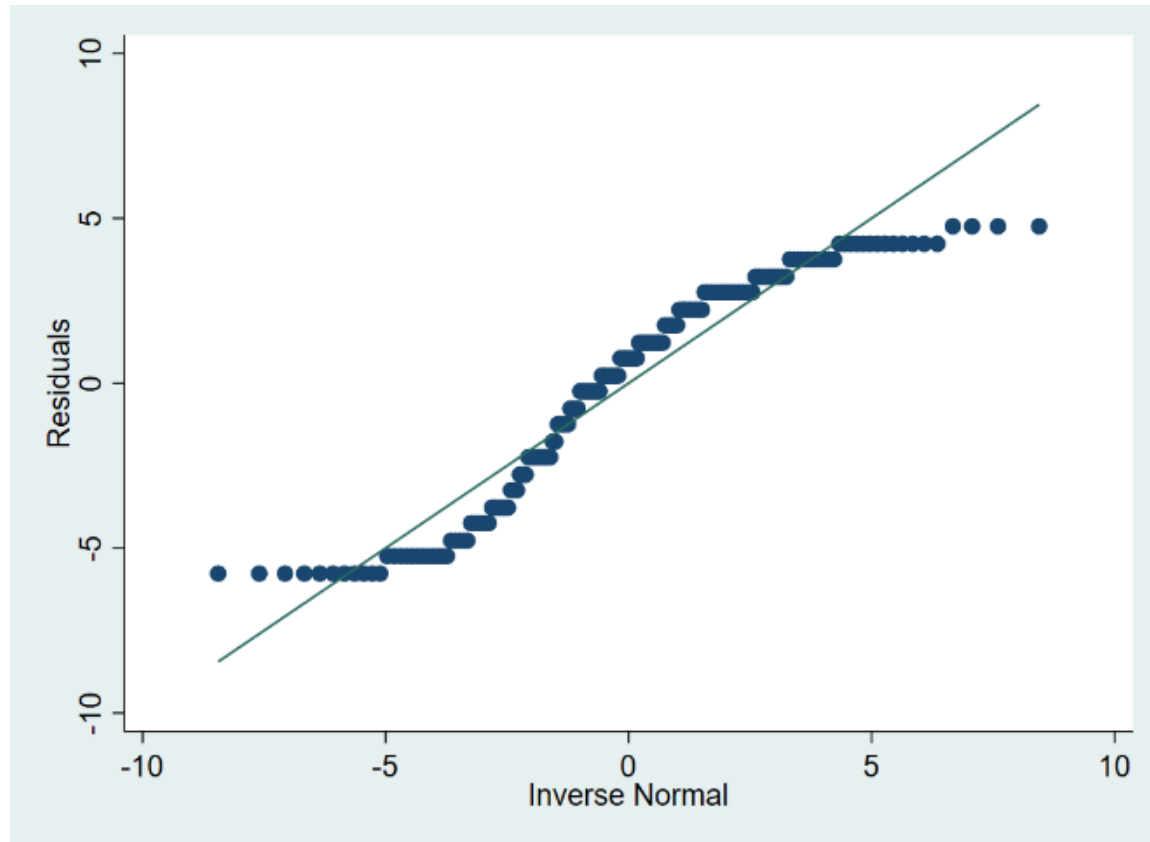<br><br>*F*(1, 172) = 0.18, *p* = .668
Average points-per-shot across first three shots	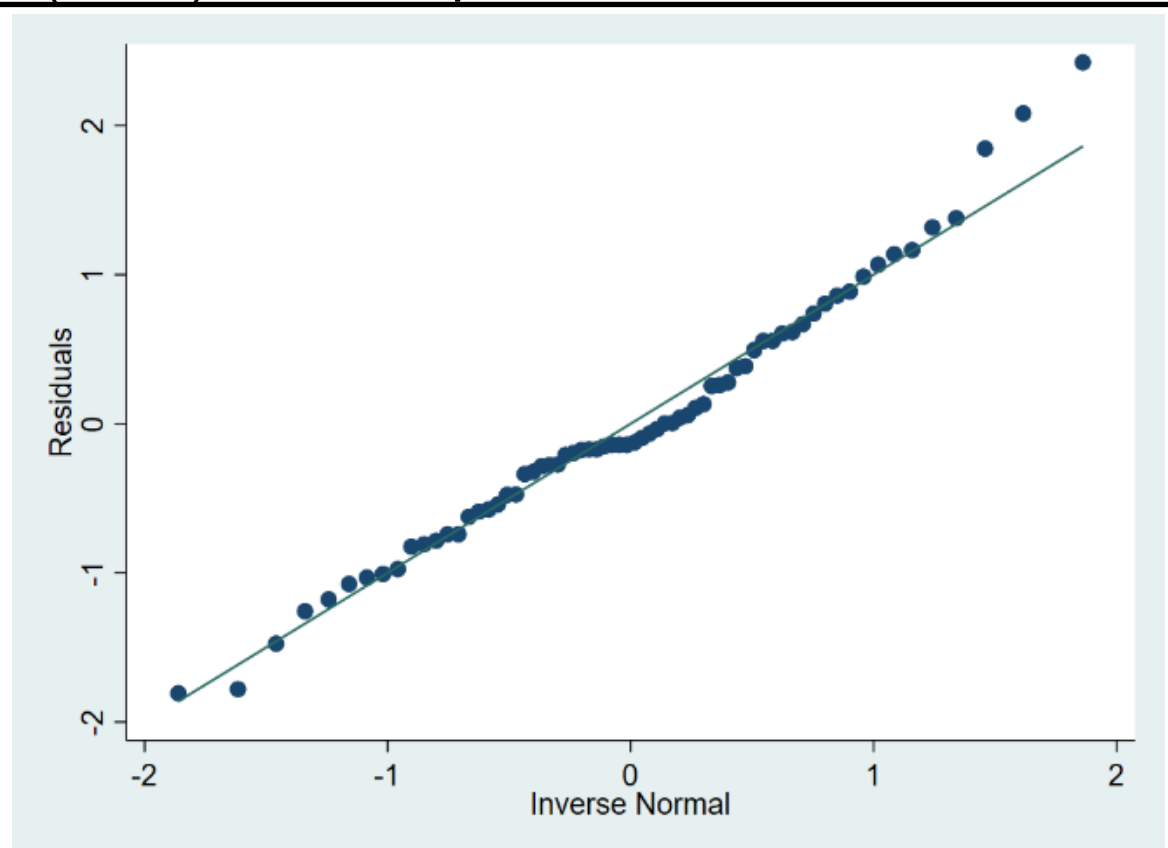<br><br>*F*(1, 66) = 5.47, *p* = .022	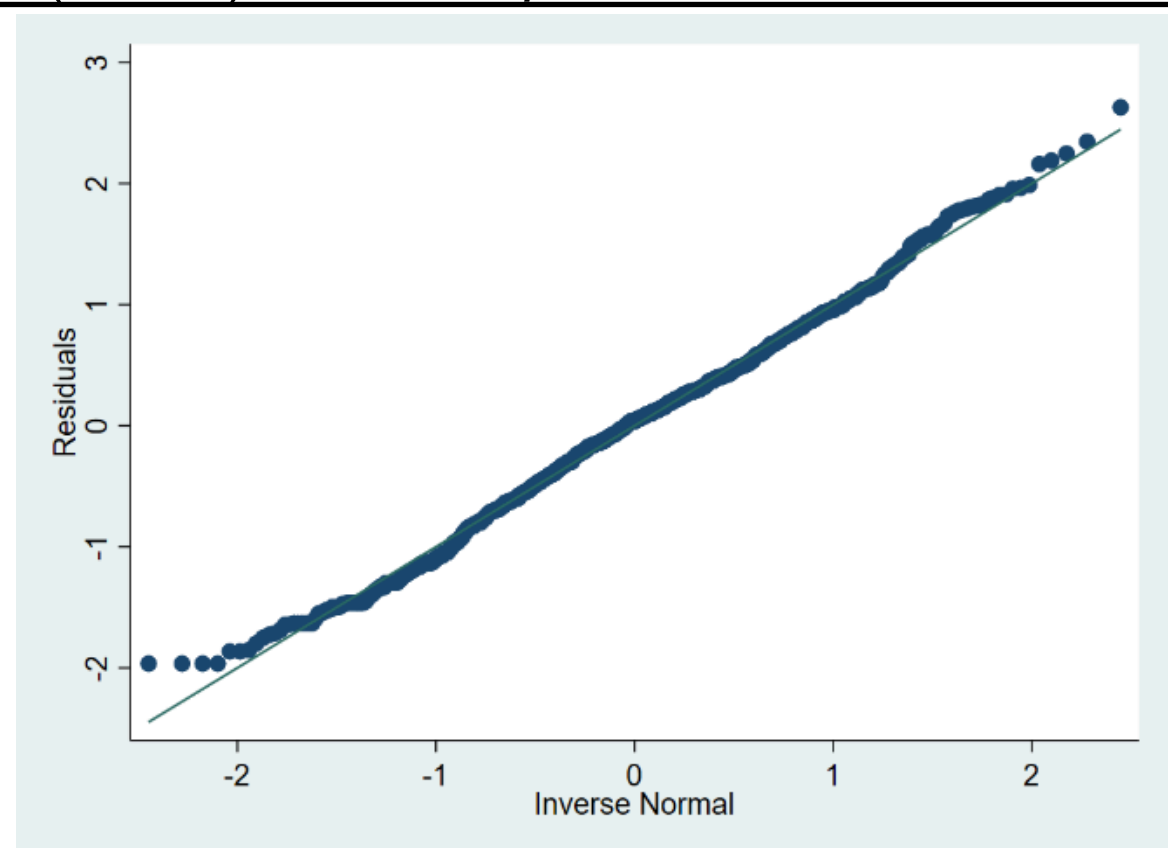<br><br>*F*(1, 973) =46.18, *p* < .001	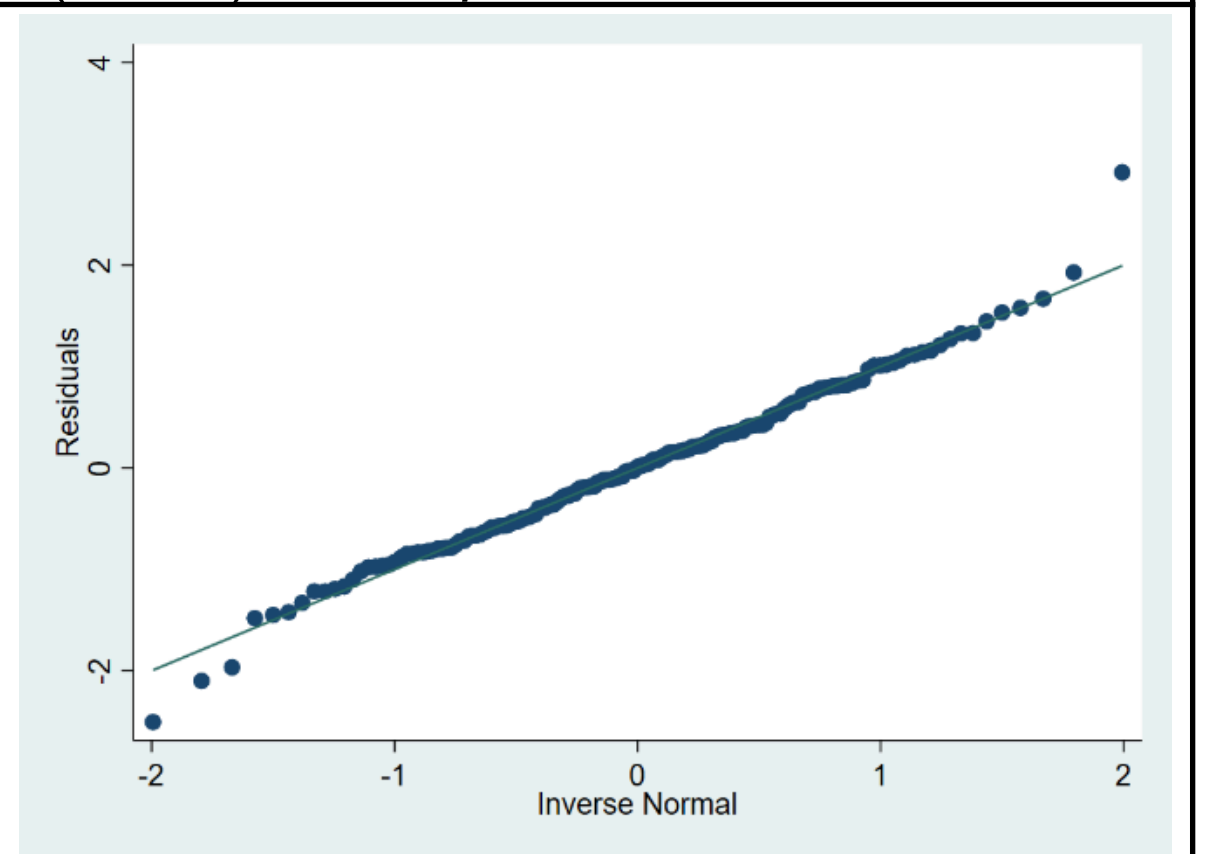<br><br>*F*(1, 172) = 4.42, *p* = .037
