# Supplementary material for: Revisiting evidence for an attentional tunneling effect caused by scarcity
Source: Nat Commun. 2026 Sep 26;17:10233. doi: 10.1038/s41467-026-78074-y (PMC13615970; doi:10.1038/s41467-026-78074-y)
Supplement: Supplementary file 2 [file 41467_2026_78074_MOESM2_ESM.pdf]

nature portfolio

Corresponding author(s): Fiona tho Pesch

Last updated by author(s): 02.03.2026

Reporting Summary

Nature Portfolio wishes to improve the reproducibility of the work that we publish. This form provides structure for consistency and transparency in reporting. For further information on Nature Portfolio policies, see our Editorial Policies and the Editorial Policy Checklist.

Statistics

For all statistical analyses, confirm that the following items are present in the figure legend, table legend, main text, or Methods section.

n/a	Confirmed	
☐	☒	The exact sample size (*n*) for each experimental group/condition, given as a discrete number and unit of measurement
☐	☒	A statement on whether measurements were taken from distinct samples or whether the same sample was measured repeatedly
☐	☒	The statistical test(s) used AND whether they are one- or two-sided<br>*Only common tests should be described solely by name; describe more complex techniques in the Methods section.*
☒	☐	A description of all covariates tested
☒	☐	A description of any assumptions or corrections, such as tests of normality and adjustment for multiple comparisons
☐	☒	A full description of the statistical parameters including central tendency (e.g. means) or other basic estimates (e.g. regression coefficient) AND variation (e.g. standard deviation) or associated estimates of uncertainty (e.g. confidence intervals)
☐	☒	For null hypothesis testing, the test statistic (e.g. F, t, r) with confidence intervals, effect sizes, degrees of freedom and P value noted<br>*Give P values as exact values whenever suitable.*
☒	☐	For Bayesian analysis, information on the choice of priors and Markov chain Monte Carlo settings
☒	☐	For hierarchical and complex designs, identification of the appropriate level for tests and full reporting of outcomes
☐	☒	Estimates of effect sizes (e.g. Cohen's d, Pearson's r), indicating how they were calculated

Our web collection on statistics for biologists contains articles on many of the points above.

Software and code

Policy information about availability of computer code

Data collection	We used the online game program we generously received access to by the original authors of the Shah et al. (2012) study. We adjusted the code to mirror our extension of the original game. The game can be accessed on our OSF project.
Data analysis	All data were analyzed using STATA, version BE 17.0, except for equivalence testing, which was performed in R version 4.4.3 (2025-02-28, "Trophy Case").

For manuscripts utilizing custom algorithms or software that are central to the research but not yet described in published literature, software must be made available to editors and reviewers. We strongly encourage code deposition in a community repository (e.g. GitHub). See the Nature Portfolio guidelines for submitting code & software for further information.

Data

Policy information about availability of data

All manuscripts must include a data availability statement. This statement should provide the following information, where applicable:

- Accession codes, unique identifiers, or web links for publicly available datasets
- A description of any restrictions on data availability
- For clinical datasets or third party data, please ensure that the statement adheres to our policy

We re-analysed two openly available existing datasets (Shah et al., 2012: http://theslab.uchicago.edu/browse/scidata; and Shah et al., 2019: https://osf.io/vzm23/). The data to our conceptual replication can be accessed here: https://doi.org/10.17605/OSF.IO/8PZW3

Research involving human participants, their data, or biological material

Policy information about studies with human participants or human data. See also policy information about sex, gender (identity/presentation), and sexual orientation and race, ethnicity and racism.

Reporting on sex and gender	We collected participants' gender identity by asking them to self-report which gender they idenfity as. We did not perform any gender-specific analyses, as we have no a priori assumptions about gender differences with regard to our research questions. Within our final sample, 42.53% identified as female, while 56.32% identified as male. 1.15% prefered not to say.<br><br>There is no demographic information for the sample of the Shah et al. (2012) publication. For the Shah et al. (2019) publication, we find that 56% of the sample identifies as female, and 44% as male (note that demographic information was missing for two participants).
Reporting on race, ethnicity, or other socially relevant groupings	We asked participants to indicate their race/ethnicity by selecting all options that apply to them from a list (Hispanic or Latino; African or African American; American Indian or Alaskan Native; Asian; Pacific Islander; White; Other (please list below)). We did so in order to get an idea of what our sample looked like. We did not perform any race-specific analyses, as we have no a priori assumptions about racial differences with regard to our research questions. Within our final sample, 85.06% identified as white, while 4.02% identified as African-American. 1.15% indicated "Prefer not to say", with the rest of the sample indicating other ethnicities.
Population characteristics	In our conceptual replication study, we asked participants for their age, in order to get an idea of what our sample looked like. We did not perform any age-specific analyses, as we have no a priori assumptions about age differences with regard to our research questions. The mean age of our sample was 40.2 years.<br>There is no demographic information for the sample of the Shah et al. (2012) publication. For the Shah et al. (2019) publication, we find a mean age of 34.0 years in the final sample (with demographic information missing for two participants).
Recruitment	We recruited participants through Amazon's crowdsourcing marketplace called mTurk. We used Amazon's mTurk as a recruitment platform to remain as consistent as possible with the original studies. For a discussion of self-selection bias and other shortcomings of this sampling method, see Aguinis et al., 2020. Participants received a flat fee of $3.00 and additionally earned $0.02 per point accumulated during the game, resulting in a performance-based bonus ranging between $0.46 and $7.03 (M = $3.20, SD = 2.17).
Ethics oversight	Review board of the University of California Berkely (CPHS Protocol Number: 2013-08-5546)

Note that full information on the approval of the study protocol must also be provided in the manuscript.

Field-specific reporting

Please select the one below that is the best fit for your research. If you are not sure, read the appropriate sections before making your selection.

☐ Life sciences ☒ Behavioural & social sciences ☐ Ecological, evolutionary & environmental sciences

For a reference copy of the document with all sections, see nature.com/documents/nr-reporting-summary-flat.pdf

Behavioural & social sciences study design

All studies must disclose on these points even when the disclosure is negative.

Study description	We conducted a quantitative experimental investigation of the effect of experiencing scarcity on the performance in a game called "Anrgy Blueberries".
Research sample	For the conceptual replication we conducted, we recruited a non-representative sample from Amazon's mTurk. Our final sample reported a mean age of 40.2; 56.32% indentified as male; and 85.06% as white (for more detail, see above).<br>We also re-analysed two datasets (Shah et al., 2012, 2019). Both dataset are publicly accessible.
Sampling strategy	We used a convenience sample from mTurk. We used the program G*Power to obtain an estimate of the sample size required to replicate the effect of scarcity on participants' time spent aiming the first shot of each level (our H1). Shah et al. (2012) found a large effect ($\eta p2 = .17$) in their original experiment and a medium-sized effect in a replication ($\eta p2 = .11$; Shah et al., 2019). Our analysis determined that replicating the medium effect would require a sample size of N = 90 for the replication (correlation among repeated measures = 0; power $1 - \beta = 0.90$).
Data collection	We sent out links to our online game "Angry Blueberries" through mTurk. Participants were then asked to play the game after giving informed consent. As this was an online study, participants used their personal computers at home.
Timing	24.11.2021 - 26.11.2021
Data exclusions	After reading the instructions of the game, participants received three comprehension check questions. Participants who failed any of these questions more than once were excluded from the game. Further, we only included data from participants who finish playing the game.

Non-participation	We recruited 200 participants. Of these 200 participants, 26 participants did not finish playing the game, resulting in a final sample of 174 participants.
Randomization	Allocation to groups was random.

Reporting for specific materials, systems and methods

We require information from authors about some types of materials, experimental systems and methods used in many studies. Here, indicate whether each material, system or method listed is relevant to your study. If you are not sure if a list item applies to your research, read the appropriate section before selecting a response.

Materials & experimental systems

n/a	Involved in the study	
☒	☐	Antibodies
☒	☐	Eukaryotic cell lines
☒	☐	Palaeontology and archaeology
☒	☐	Animals and other organisms
☒	☐	Clinical data
☒	☐	Dual use research of concern
☒	☐	Plants

Methods

n/a	Involved in the study	
☒	☐	ChIP-seq
☒	☐	Flow cytometry
☒	☐	MRI-based neuroimaging

Plants

Seed stocks	*Report on the source of all seed stocks or other plant material used. If applicable, state the seed stock centre and catalogue number. If plant specimens were collected from the field, describe the collection location, date and sampling procedures.*
Novel plant genotypes	*Describe the methods by which all novel plant genotypes were produced. This includes those generated by transgenic approaches, gene editing, chemical/radiation-based mutagenesis and hybridization. For transgenic lines, describe the transformation method, the number of independent lines analyzed and the generation upon which experiments were performed. For gene-edited lines, describe the editor used, the endogenous sequence targeted for editing, the targeting guide RNA sequence (if applicable) and how the editor was applied.*
Authentication	*Describe any authentication procedures for each seed stock used or novel genotype generated. Describe any experiments used to assess the effect of a mutation and, where applicable, how potential secondary effects (e.g. second site T-DNA insertions, mosiacism, off-target gene editing) were examined.*
