# Supplementary material for: Revisiting evidence for an attentional tunneling effect caused by scarcity
Source: Nat Commun. 2026 Sep 26;17:10233. doi: 10.1038/s41467-026-78074-y (PMC13615970; doi:10.1038/s41467-026-78074-y)
Supplement: Supplementary file 3 [file 41467_2026_78074_MOESM3_ESM.pdf]

nature portfolio

Peer Review File

Revisiting evidence for an attentional tunneling effect caused by scarcity

Corresponding Author: Dr Fiona tho Pesch

This file contains all reviewer reports in order by version, followed by all author rebuttals in order by version.

Version 1:

Reviewer comments:

Reviewer #1

(Remarks to the Author)
First of all, I'd like to thank the authors for this interesting submission. The main topic of this paper—examining the verisimilitude of the tunneling hypothesis and attentional neglect in the context of scarcity, through robustness testing of the original data and a conceptual replication using a new dataset—is highly relevant to a broad readership. There are many positives to the paper. Besides the relevance of the topic, it presents results that mostly contradict the original findings. I find publishing contradictory/null results extremely important for the progress of the field. I appreciate how the authors complement their findings with pragmatic explanations, rather than over-psychologizing them. The writing is mostly clear and easy to understand. As a strong proponent of open science practices, I particularly appreciate the authors' adherence to these principles.

However, this is one of those papers that left me with mixed thoughts about how to evaluate it. Although I'm very sympathetic to the authors' efforts and find their explanations much more plausible compared to the original claims, I'm still skeptical about the findings because of two main factors: (1) ecological and external validity (far-transfer) and (2) data quality. Given the design of the study (i.e., data re-analysis and conceptual replication), both are at least partly outside the authors' control. Much of my criticism is, therefore, directed toward the validity of the original claims. I'll elaborate on these points below and include a few comments regarding the methodology used. I hope the authors will find my feedback useful.

Ecological and external validity:

Nicely illustrated in the first two sentences of the abstract, the main goal of the tunneling hypothesis is to explain the psychological mechanisms behind poverty perpetuation. My main concern is that the experimental situation used in this study is, at best, a proxy for real-world scarcity settings, and it is unclear to what extent these findings can be transferred to the real world, which is far more complex.

In the "Angry Blueberries" game and similar tasks, participants operate in an artificial environment that is safe, has clear rules, and offers limited actions. The consequences of participants' decisions (e.g., shots, borrowing, insurance) are straightforward and apply only to the specific task for a limited time, unlike the complex decisions individuals in poverty face in their everyday lives. Real-world decisions involve more emotional (e.g., shame, frustration, anxiety), social (e.g., family needs, marginalization), and environmental factors (e.g., employment, healthcare, unexpected events) than those typically captured in a lab setting. Particularly in the context of scarcity, ongoing financial instability, cognitive load, social pressures, and systemic barriers to accessing resources can dramatically alter how people make decisions, thus creating mechanisms that may not be fully replicated in a simplified task.

A major role is likely also played by temporal factors. While real-world scarcity typically involves sustained, long-term lack of resources and opportunities, these simulated tasks and experimental manipulations of resource availability last only a few minutes. Participants can withdraw at any time, and the tasks have no real-life consequences. Neglecting these factors may result in discovering oversimplified patterns, rather than understanding the true behavioral mechanisms behind poverty perpetuation.

Again, I'd like to emphasize that I fully agree with the authors' reasoning that attributes the observed decision-making of the participants to the game design rather than to the psychological mechanisms behind the conditions of scarcity. I've been

considering how this can be improved in the paper—my suggestion would be to put more focus on the limitations of the experimental task used and potentially further investigate whether the current findings hold across different games and scenarios. This will be a very beneficial and essential step before attempting to explore how to test the verisimilitude of the hypothesis under real-world circumstances.

Data quality:

My second major concern is the quality of the data. Both the authors of the original study and the authors of the current study recruited participants using Amazon MTurk. MTurk is notoriously known for issues related to insufficient data quality (e.g., Chmielewski et al., 2019; Douglas et al., 2023; Webb & Tangney, 2022).

Regardless of the potential issues with MTurk data quality, there are other factors that might be affecting the results. The authors of the present study addressed careless responding by incorporating three comprehension checks after the instructions (I found no information about handling careless responding in the original paper). However, almost no further information about the participants and potentially relevant variables is provided. I assume that participants were randomly assigned to the experimental condition (poor vs. rich). However, with relatively modest sample sizes (N = 68 and N = 174; note that the authors of the current study also re-analyzed a self-replication of the original claim but have kept these results in the supplementary material), random assignment may not fully account for individual differences that could affect performance and decision-making. Variables such as previous experience with similar games, stress levels, cognitive abilities, or enjoyment of the task could introduce variability that the study does not control for. Measuring and accounting for these factors would provide stronger evidence for or against the tunneling hypothesis, as it would help isolate the psychological mechanisms at play and explore the influence of alternative explanations.

Following this, the authors state that they excluded data from participants who did not finish playing the game. Not finishing the game could indicate several possibilities: a participant may have found the game too difficult or frustrating (especially in the “poor” group), perhaps their performance didn’t meet their expectations, or maybe the game was boring, too easy, etc. Essentially, this presents a missing data problem, and the reasons for not finishing the task are likely unknown. Excluding these participants might introduce bias stemming from conditioning on a collider (experimental condition -> finishing the game <- performance). Therefore, taking a closer look at these participants and running a sensitivity analysis on all available data might reveal slightly different patterns. Lastly, in their preregistration, the authors state that they aimed to collect data from at least 200 participants. Since they report no deviation from the preregistered protocol, the effective sample of N = 174 likely indicates that at least 13% of the original sample either failed the comprehension checks or did not finish the game.

I also wonder how the authors dealt with surprisingly fast or slow shots or other potentially peculiar patterns (e.g., always borrowing). I understand they log-transformed the reaction times, but based on my quick glance at the data, I believe this issue deserves more attention (any substantial data cleaning should be reported explicitly) and ideally should be addressed through a sensitivity analysis.

Minor comments:

I’d suggest adding more clarity to the aims of the study and the corresponding design. Although they are mentioned throughout the text or implied, summarizing them would help readers better navigate the study. Additionally, mentioning the re-analysis of the self-replication by Shah et al. (2019) in the main text might provide a richer context regarding the stability of the claims.

The authors correctly decided to utilize equivalence testing to determine whether the outcomes of the experimental conditions are practically equivalent (i.e., whether the effect is within the equivalence bounds). However, they set their SESOI based on statistical criteria (i.e., an effect that the original study had a 33% power to detect) rather than a theoretical or practical justification. To move beyond simply re-analyzing and replicating the original findings, I’d suggest justifying the effect size based on its theoretical or practical significance. Additionally, the authors write that “Equivalence testing revealed that the study was underpowered to support the conclusion that the ‘poor’ and the ‘rich’ were statistically equivalent.” Can the authors clarify this in the main text? It is one of the focal claims, and without additional context (though partly available in the supplementary materials), it is difficult to determine what the SESOI was and to interpret whether the confidence interval was too wide and/or outside the predetermined equivalence region.
Related to the above, it would be beneficial to present a power curve for hypothetical sets of effect sizes and sample sizes, rather than relying solely on point estimates from the power analysis. This would be especially helpful given that the SESOI is somewhat arbitrary, and the study used N = 174 instead of the preregistered N = 200, on which the original power analysis was calculated.

I checked the supplementary materials on OSF. Kudos for sharing everything, including materials and instructions—that’s how transparent and responsible research should be! I noticed that the data collection for the conceptual replication also involved other experimental tasks as well as additional measures (https://osf.io/wf57c?view_only=19edfe9ed67f40009b677fb11c8c60f8). In the informed consent, the authors explicitly mention that participants would be asked to complete several tasks, which would take around 120 minutes. I fully understand that it’s common practice to collect more data at the same time. However, I wonder—could this have affected data quality and participants’ decision-making? Completing multiple experimental tasks and filling out questionnaires might become exhausting after a certain point. If this supplementary material information is correct, did the authors account for this, perhaps by randomizing the order of tasks? Furthermore, I noticed that the preregistration includes additional analyses (e.g., mediation through time and risk preferences), which are not mentioned in the paper. Just to clarify—were only some parts of the preregistered

analyses presented in the paper, or am I missing something? Were there any other (substantial) deviations from the preregistration that should be disclosed in the paper?

Overall comments and suggestions:

As much as I'm sympathetic to the authors' efforts and think that their pragmatic explanations of the tunneling hypothesis are valid, a more rigorous continuation of the current lab-based approach is needed to address the research question more thoroughly. Since collecting data in real-world settings would be too complex, potentially unethical, and likely too far removed from the authors' aims, continuing with controlled lab-based tasks is the most appropriate path forward. To build on this, I suggest conducting a multiverse analysis, varying and synthesizing multiple reasonable data cleaning and hypothesis-testing possibilities. Additionally, expanding the experimental scenarios—ideally with a within-person design that varies levels of scarcity throughout the game—could offer further valuable insights. Incorporating more relevant variables into the experimental design and analyzed models could also shed further light on the behavioral mechanisms underlying decision-making in the context of scarcity.

Again, I'd like to express that I'm aware much of the above criticism is related to the original claims. However, I'd like to see results that are robust enough to convince the field and allow researchers to confidently build upon them, helping the field move forward.

I hope this feedback helps.

All the best.

(Remarks on code availability)
I reviewed the code briefly, focusing on the parts written in R, as I'm not very familiar with Stata syntax, where most of the analyses and data cleaning were performed. From what I can tell, the code looks alright.

Reviewer #2

(Remarks to the Author)
I very much enjoyed engaging with this re-look at a seminal finding in the psychological study of poverty, a finding that has formed the foundation of a set of strong theoretical assumptions, most prominent of which is the claim that scarcity leads to 'tunneling'. It has long bothered me that the mechanistic assumptions made by the team who published the original set of studies on this have not been adequately scrutinized, preventing the opportunity for a subfield of the study of scarcity/poverty to develop in a theoretically precise and robust manner. Absent this strong theoretical footing, the recent failures to replicate some aspects of this work have cast a shadow on the field more generally, despite it being a promising and important one to advance. This matters not only for the health of a nascent subfield cutting across psychology, behavioral economics, and social policy, but also for the wider impact of the work, as the notion of a 'scarcity mindset' is now widely discussed in policy circles, in designing interventions for NGOs combatting poverty, and indeed in the plethora of self-help videos claiming to help people escape such a mindset. It is thus of critical importance, for the social and behavioral sciences and for the public debates that they inform, that the scientific base is as clearly and precisely understood.

The current paper does this critical work. It takes the early theoretical claims of what has become known as 'scarcity theory' at face value, digging into their implications, and then adjudicating on whether or not their empirical base is strong enough to warrant the claim that scarcity leads to a circumscribed form of enhanced performance due to greater focus, at the same time as suboptimal, present-oriented decisions due to attentional neglect. The clever reanalysis of the core findings supporting these claims is thoroughly convincing as to the need to reconsider both conclusions. Ideally, this would be the kind of analysis that is conducted in response to attentive reviewers of the original paper, but it is never too late for that work to be done! The additional analysis of the 2019 paper, in addition to the current authors' own conceptual replication, makes a strong package worthy of publication in a top pan-science journal such as Nature Communications.

I have very little feedback to offer as I found the writing to be clear and concise, the analyses to be appropriate and the reporting transparent and comprehensive. I did find I wanted to hear more from the authors about the implications of their argument, in the discussion, that much of what the poor face are not a case of decision-making constrained by a particular mindset that differs from that of the rich, but decision-making constrained within a different decision space or set of parameters than that of the rich. This basic point about the importance of structural constraints on decision-making has been neglected by the field, and is neatly demonstrated in this re-presentation of the parameters of the Angry Blueberries game. Perhaps another few lines on what implications this might have for how to research scarcity/poverty going forward would help increase the impact of the paper.

One place where I felt more clarity could be offered was on lines 497 to 500 on p.90, in the supplementals, where it appears the authors say it should be equivalent, then that this result implies it's not equivalent, then say therefore it was underpowered…? The logic of how this particular finding from an equivalence test implies that a test is underpowered, as opposed to evidence against the authors' prediction, could be spelled out more clearly.

I also caught the following minor grammatical issues/typos:
- Gamma footnote – repetition in saying '80% power…with a power of .8'
- Line 487 p.27 typo 'wohle'
- Line 498 p. 90 'did not reveal statistical equivalence…on neither the first shot…nor the second…' – should be 'on either the

first shot…or the second' (or 'revealed statistical equivalence…on neither the first shot…nor the second'
- Line 557 p.31 'significantly interaction effect'

All in all I was impressed with this paper and convinced that its publication would be a highly important contribution to the field, even if it challenges some core assumptions of thinking in that field…including that of my own!

Jennifer Sheehy-Skeffington.

(Remarks on code availability)

Reviewer #3

(Remarks to the Author)

The paper presents some re-analyses and re-interpretations of one study in Shah et al.'s multi-study 2012 paper, entitled "Some consequences of having too little." The original analyses are successfully replicated; the authors then present "additional analyses" to argue against some of the interpretations offered by the original authors. The critique mostly concerns the original authors' proposed explanations concerning participants' attention allocation, whereas the additional analyses "suggest instead that the observed behavioral differences between 'rich' and 'poor' participants can be more parsimoniously explained by the game's structure."

The distinction is hard to interpret. Since participants in the original studies were randomly allocated to "rich" and "poor," determined solely by the game's structure, it seems clear that it is, in fact, the game's structure that leads to the observed differences. How much of the data is attributable to what behavioral factors remains to be better understood. But since there are no actual "rich" and "poor" participants in the study, only participants made rich or poor by the budgets allocated to them by the game, the observed behavioral differences are bound to be explained by the game's structure.

The authors seem to think that the need of "poor" participants to borrow more often than the rich is somehow a problem, but, again, that appears to be in the nature of the study's design. Borrowing at very high interest in the original game was designed to be potentially harmful (as it is in life). The finding, apparently, is that being poor more easily leads one to take expensive loans, to their own detriment, whereas being rich allows one more easily to avoid it. As a result, the studies find that the poor, when they can borrow, do worse than when they cannot, contrary to the rich.

The authors write, "our additional analyses show that when examining only those cases in which participants had the opportunity to borrow, 'rich' participants always borrowed, while 'poor' participants only borrowed in 69% of cases." That is certainly false. Both rich and poor participants in the borrowing conditions were allowed to borrow throughout. What the authors might mean is not "the opportunity to borrow" (which both "rich" and "poor" have throughout the borrowing condition) but "having gotten to the point where one needs to borrow." That is a very different thing than having the opportunity to borrow, and here the comparison seems strange: A "rich" participant who needs to borrow if they want to continue has no budget left – they are now "poor." Again, if I understand correctly, there is nothing "poor" or "rich" about the participants, other than the budget they have. A participant who decides to borrow with noting left is a poor participant borrowing.

Much of the focus here is around the original paper's interpretation of the allocation of attention. The original authors proposed to define the extent of participants' focus by measuring how long participants spent aiming each shot in the game. The present Table 2 contrasts the original observation, "Amongst participants who could not borrow, 'poor' participants spent significantly more time aiming the first shot of each level," with the authors' current "additional analyses," namely, "Although these 'poor' participants who could not borrow spent longer aiming their first shot of each level compared to the 'rich', they did not earn significantly more points on this shot compared to the 'rich'." The "additional analyses" are that "the longer aiming time of 'poor' participants did not actually translate into better outcomes for those shots." This, the present authors argue, "casts doubt on whether 'poor' participants' longer aiming time reflected their greater focus on the task." But why is that? One can think of many tasks, both easy and hard, where greater focus would not lead to better performance. In fact, it would seem highly ill-advised to argue that one's relative lack of success at a task shows a lack of focus.

The paper describes a "conceptual replication experiment," involving the hypothetical purchase of insurance, where no significant difference in the tendency to purchase insurance is observed between "rich" and "poor" participants, except that "poor" participants were less likely to purchase negative expected value insurance. While the authors propose this as somehow providing additional evidence against the hypothesis that scarcity leads to attentional neglect of information, that interpretation remains entirely unclear. In fact, part of the argument by the original researchers is that scarcity often leads to greater attention to relevant information, which, to the extent there was any difference observed, it's what was observed here.

Interestingly, in their discussion of tunneling, a concept closely related to the aforementioned comments on focusing, the authors argue that, "Absent a clear
definition of what should fall within vs. outside the tunnel, it is impossible to falsify the theory, because it becomes possible, post-hoc, to attribute any process or decision that is enhanced among the poor to attentional focus, and to attribute any process/decision that is impaired to attentional neglect." This sounds right. And in light of this, one wonders about the authors' attempt to falsify the interpretations proposed in the study they are critiquing. The notion that participants' focus is better gauged by successful shots rather than time spent is precisely the sort of concept lacking a clear definition that the

authors so wisely advise against.

The authors conclude that, “the better performance of the 'poor' in the original experiment did not stem from their greater focus on the task, as proposed by the original authors.” Better than what? The only times the word “better” appears in the original study (apart from other studies concerning previews of information) is in the observation that “poor participants fared better when they could not borrow than when they could.” While, of course, it could be true that the observed effect does not stem from a greater focus, this reviewer sees no evidence supporting this conclusion.

The authors are right to point out that “a better understanding of the cognitive and behavioral consequences of experiencing scarcity is important for informing policy recommendations about how to combat poverty around the globe.” In that spirit, the authors would do well to present research that enriched our data and our understanding – the present critical examination sheds little light on the issues of interest.

(Remarks on code availability)

Version 2:

Reviewer comments:

Reviewer #2

(Remarks to the Author)
I am happy that the editor decided to take a second look at this paper, as I was supportive of it the first time around and disappointed to see that it did not progress further at Nature Communications.

Having now read the other reviews, I can see why the authors have resubmitted it for additional consideration and think they are fully justified in doing so.

The first review is focused on limitations of the ability of the experimental paradigm to shed light on decision-making in response to real-world financial scarcity and thus any potential psychological mechanisms at play therein. The first limitation is one of ecological validity, and the second questions the ability or relatively small sample sizes used in random assignment to rule out the role of individual differences that may be shaping game behavior. These criticisms are important and ones that I would also make of much experimental research in the field, including my own.

However, I concur with the authors that they are not pertinent to the evaluation of the submitted manuscript, as the manuscript itself does not attempt to use such experiments for the above purpose. Rather, it aims to take a critical look at two key claims from a seminal paper in this field which was published in possibly the leading journal in all of the sciences. If such a scientific paper published in a highly respected, pan-disciplinary journal after rounds of rigorous peer review is found to be flawed in the claims it makes, it is essential that the scholarly community is informed of this in another major outlet, so that the record can be corrected. The need for correction is even more urgent given that this original paper has been cited nearly 2,000 times (according to Google Scholar) since its original publication, and is having an impact in the applied fields of international development (see, for example, the World Development Report 2015: https://www.worldbank.org/content/dam/Worldbank/Publications/WDR/WDR 2015/WDR-2015-Full-Report.pdf) and domestic poverty alleviation programs (see, for example, the discussion of this research program in https://s3.amazonaws.com/empath-website/pdf/Research-UsingBrainScienceDesignPathwaysPoverty-0114.pdf).

I am thus satisfied with the authors’ response to Reviewer 1 concerning the empirical limitations of the original work. I am also happy with other responses to R1 and the clarifying text now added to the manuscript concerning participant dropout, exclusions, and deviations from the preregistration. The addition of power curves provides further insight into the power of the study given a range of SESOI. Relatedly, the explanation of how equivalence testing works in response to my own (naïve) comment on this in my review, as given by the authors in the response memo and in the revised manuscript, is satisfactory.

All in all, I agree with the authors that changes made in response to the detailed and thorough first review have improved the manuscript.

I’m also happy with how the other comment in my review was addressed, in terms of the short elaboration of the importance of these findings for the study of decision-making in poverty.

Finally, I do not think that the issues raised by the third reviewer challenge the credibility or importance of the current work, though they are useful as they have prompted the authors to add clarifying text that improves the manuscript. They key claim of the original paper by Shah et al. is indeed about the psychological mechanisms through which being ‘rich’ or ‘poor’ leads to particular decision-making patterns, not about the structural constraints imposed by the game. Yet the current manuscript convincingly demonstrates that for two of the key claims, it is the structural constraints that gave the appearance of a psychological effect where there is not in fact sound evidence for one. It is important for the scientific record, for policy applications, and indeed to correct how research in this area is taught in graduate courses, to know (1) that the increased

time-per-shot exhibited by ‘poor’ participants is not a case of the kind of increased focus that improves performance, and (2) that the greater borrowing by ‘poor’ (versus ‘rich’) participants is not a case of being more likely (than the ‘rich’) to borrow where there is an opportunity to borrow. I am satisfied with the authors’ response to R3 on these points and the clarifications made to the main text as a result.

Just one typo I noticed in the latest version on line 579: ‘distribution of participant’ is missing ‘s’ et the end.

In summary, I continue to be strongly supportive of the publication of this work in Nature Communications, and do not think there are persistent issues that could justifiably prevent this.

Jennifer Sheehy-Skeffington.

(Remarks on code availability)
n/a

Reviewer #3

(Remarks to the Author)
As before, the paper presents re-analyses and re-interpretations of a study in Shah et al.’s 2012 paper, entitled “Some consequences of having too little.” The original analyses are successfully replicated and the authors then present “additional analyses” to argue against some of the interpretations offered by the original authors.

The paper presents several prongs of critique: A different definition of attention, an analysis comparing the borrowing behaviors of the poor versus the rich who are out of budget, a new “insurance” intervention, and a structural analysis of the borrowing experiences of the ‘rich’ and ‘poor.’

First, the authors propose a different definition of attention and find that, using this new definition, attention does not explain the findings. Their new definition requires of attention to lead to improved performance. The additional analyses showing that the longer aiming time of 'poor' participants did not actually translate into better outcomes, it is argued, casts doubt on whether 'poor' participants' longer aiming time reflected a greater focus on the task. This is an argument with no support. As pointed out before, equating attention with better outcomes is highly problematic. It is certainly not any more plausible than the original interpretation of attention. Among other things, Shah et al. observe, “The amount of borrowing by the poor was significantly correlated with measures of engagement. On rounds where poor participants borrowed, the average amount of time spent aiming each shot in their paycheck correlated positively with how many shots they subsequently borrowed.”

Whereas the original Shah et al. paper presents five studies, the present critique of that research agenda looks at only one. Referring to Shah et al.’s paper, the preset authors write: “While the authors claim that Studies 3 to 5 provide additional supporting evidence, they acknowledge that these studies do not constitute direct evidence for the proposed cognitive mechanisms of tunneling.” It’s hard to know what to make of this claim. The word “tunneling” never occurs in the Shah et al. paper. And contrary to the authors’ suggestion, Shah et al. write, for example: “Experiment 5 offers more direct support for the notion that scarcity creates attentional neglect.”
(Reviewer 1 laments participants were all recruited through MTurk. In fact, Study 3 in the original involved participants in a lab.)

Next, the authors run a new “insurance” manipulation. And while the manipulation fails to observe any differences, the authors claim that “These findings provide additional evidence against the hypothesis that scarcity leads to attentional neglect of potentially beneficial information that does not pertain to present needs.” That’s a lot of evidence provided by a null result. There are obviously tons of other possible explanations for why this new intervention did not work. The authors explain: “based on the original authors’ theory that scarcity causes individuals to focus on immediate needs while neglecting future consequences, the logical prediction would be that poor participants should be less likely to purchase insurance.” This is strikingly weak. First, even if those in scarcity were to neglect future consequences, what ensures that those not in scarcity would buy more of said insurance? Maybe it’s bad insurance? Maybe participants failed to notice it? In fact, most standard treatments of insurance argue that the poor should buy *more* of it than the rich. So if they didn’t, maybe that shows they failed to attend to it sufficiently, as per the original authors’ theory. This “conceptual replication” with null findings sheds little light on the issues. (Further confusing is the fact that we know in the real world the poor do buy less insurance than the rich. So no difference is the 'wrong' result, unless, of course, one assumed all of this has nothing to do with simulating poverty.)

The present authors present an analysis comparing the behavior of ‘poor’ participants to that of ‘rich’ participants who are out of budget, and they find that there’s no difference. This is entirely perplexing. There are no ‘poor’ or ‘rich’ participants in the original study – only participants with smaller or larger budgets. A ‘rich’ participant without a budget is a poor participant. In fact, the comparison the authors run is between poor participants and poor participants and they find no difference. (Imagine we assigned one group of participants to consume alcohol (the drinkers) and another group we gave only water (the non-drinkers). Our hypothesis was that drinkers would then drive less well. What we did next, for a truly “clean” comparison was to compare the non-drinkers to the “drinkers” who abstained from drinking. And we found there was no difference, thus disproving that alcohol hampers driving. This is roughly the logic of comparing the ‘poor’ to the ‘rich who have nothing.’)

In fact, because there are no rich and poor participants, since they are all randomly assigned, the only difference between 'rich' and 'poor' participants is the budget they have been allocated. The authors explanation, "our analyses suggest instead that the observed behavioral differences between 'rich' and 'poor' participants can be more parsimoniously explained by the game's structure," is perplexing. Of course it's the game's structure. The only question is what in the structure yields the observed differences.

The flavor of the present paper is one of frustration. The authors are frustrated by the original work and look for ways to litigate it. But the critique come off as contrived. The authors write: "We show that the behavioral effects of overborrowing cannot be attributed to attentional neglect, but were instead due to differences in base rate frequencies with which the 'poor' and the 'rich' faced the option to borrow." Shah et al. (2019; the paper the present authors refer to and re-analyze) write: "We find weaker evidence for the hypothesis that scarcity-induced focus leads to attentional neglect. We fully replicated the experiments wherein greater scarcity led to more high-interest borrowing." It's very hard to tell the difference between those two claims.

The original Shah et al. study clearly is limited in scope, highly artificial, and requires further understanding. And its applicability to actual contexts of poverty certainly deserves further study. Unfortunately, beyond the frustration it expresses, the present paper does not shed light on the issues.

(Remarks on code availability)

Reviewer #4

(Remarks to the Author)
First of all, I think this is a great initiative to re-examine the seminal work by Shah et al. (2012). This paper received a lot of attention and opened an intriguing and important perspective on poverty with a huge impact on subsequent studies in particular and the literature on poverty in general. However, it also sparked a lot of skepticism (for good reasons), and the current paper is an attempt to reassess a core component of the theory by critically analyzing the empirical evidence and providing new evidence.

In my view, scarcity theory can be criticized on at least two fundamental grounds. First, while the groundbreaking studies – along with some follow-up work by different authors – have undoubtedly shaped the discourse, they are largely based on small samples and often lack research transparency. While such limitations were more widely accepted in the 2010s, the field has since evolved, and we must now push for greater transparency, replicability, and open science initiatives. Scientific progress depends on rigor, and if a theory is built on limited empirical evidence or flawed conclusions, it is essential to reassess its validity with stronger methodological standards.
Second, the core concepts of scarcity theory are only loosely defined. While this flexibility has allowed for broad applicability and adaptability – sometimes serving as a defense against criticism – it also introduces significant challenges. When concepts are ambiguous, it is difficult to pin down precise tests and measurements, leading to inconsistencies in findings and difficulties in theory advancement. This lack of conceptual clarity may explain why accumulating evidence increasingly challenges some of the theory's foundational claims, such as the bandwidth tax. The present paper is a compelling illustration of this issue, and the discussion provides valuable insights into how the field can move forward.

In this regard, I strongly agree with Reviewer 2 that for scarcity theory to advance meaningfully, it requires a firmer theoretical foundation. Strengthening conceptual clarity will enhance both its explanatory power and empirical applicability, ensuring that future studies can provide more robust and conclusive evidence either in support of or against its key propositions. Given its critical engagement with these issues, the current paper is highly relevant, well-argued, and deserves recognition.

However, I also see room for improvement. First, the sample size of the conceptual replication appears too small, especially considering that MTurk is known for its lower data quality. I also could not find information on quality controls, such as attention checks, etc. Although I see the reasons for using the same platform, I would have chosen a high-quality platform given the budget constraints. In the end, there are concerns about the reliability of the findings that outweigh the benefits of being as close as possible to the original study. Second, I find the strong focus on Study 2 unconvincing. If the existing data does not allow for a proper test of the attentional shift or tunneling hypothesis, this should be stated explicitly. However, the base rate argument should also apply to Studies 3 and 4. Study 5, which Shah et al. (2012) cite as providing "more direct support for the notion that scarcity creates attentional neglect," only reports performance results without addressing whether the "poor" participants actually had too little time to notice the previews. The authors should engage with this study as well. After all, demonstrating that tunneling is not at work in Study 2 does not mean it is absent in the other studies. (The game structure argument, however, applies in all studies.) Third, I have concerns about the reanalysis of the shots. The assumption that they are independent is problematic, as they likely depend on previous attempts, successes and failures, remaining targets, and other contextual factors. The analysis must account for these dependencies. A simple approach would be to average the first three rounds, but more sophisticated methods could be used for robustness.

(Remarks on code availability)

Reviewer #5

(Remarks to the Author)
Thank you for the opportunity to review “How does scarcity impact attention? A critical examination of the tunneling hypothesis.” I was particularly pleased to see the authors adopt equivalence testing and offer the following comments aimed at strengthening that component for a future revision.

Clarifying the SESOI
Deciding on an equivalence bound is admittedly challenging, yet it is fundamental to interpreting phrases such as “no difference.” At present the bound is anchored to a statistical benchmark (33% power), which is a useful starting point, but readers cannot judge the practical meaning of your results without knowing why a deviation of that size—or smaller—would be considered trivial in this context.

Discussing the bound in each interpretation
Whenever you conclude equivalence, consider reminding the reader to what the effect is equivalent (e.g., “within ±0.18 d, the smallest effect we have deemed practically important”). That transparency lets the audience evaluate the claim rather than the procedure.

Acceptable flexibility
It is perfectly reasonable to propose an exploratory SESOI—controversial or not—provided the rationale is explicit. You might present:
* a primary SESOI with your best theoretical or applied justification,
* a more liberal and a more conservative alternative (as you already explore in the sensitivity curves), and
* a brief discussion of how conclusions shift across them.
This lets future work refine the bound while still giving the present study a clear decision rule.

Relying on field expertise
Even though the task is artificial, you as domain experts are in the best position to offer an initial bound—whether grounded in prior literature, participant performance norms, or stakeholder costs.

By adding this targeted justification (and keeping the excellent sensitivity analyses), the manuscript’s equivalence claims will be both methodologically sound and far more impactful.

(Remarks on code availability)
Code was easy to follow and clear. No README file but do not think it was necessary.

Version 3:

Reviewer comments:

Reviewer #2

(Remarks to the Author)
I am satisfied that the authors have addressed all reviewer concerns, and think that the additional analyses run increase the contribution made even further. The clarifications and supplemental analyses also aid in reader interpretation.

(Remarks on code availability)
N/A

Reviewer #3

(Remarks to the Author)

The authors argue that the Shah et al. paper they are critically examining has had a lot of influence, and that, therefore, corrections and a deeper understanding are essential. That sounds right. Unfortunately, this paper does not provide the kind of analyses that helps move the research forward. It starts with an alternative interpretation for which the authors agree they have no evidence, it then establishes a lack of correlation contrary to the original paper, which the original authors have already established in follow-up work, and it continues with two experiments that shed absolutely no light on the issues at hand.

To begin, the present authors replicate some of the central analyses, and then “contest the original claims.” They point out that although ‘poor’ participants aimed longer, which Shah et al. interpreted as a sign of greater focusing, the ‘poor’ did not do better. This paper is purportedly a look at “tunneling,” an attentional phenomenon. The original authors saw “time spent” as a proxy for attention. The present authors’ critique is that the extra time spent did not improve performance. The present authors have zero evidence contradicting the original notion of greater focus among the ‘poor’ (they write: “Note that we do not present any evidence against the tunneling hypothesis; rather, we highlight the lack of evidence supporting it”); their objection is that the ‘poor’ did not do better, and therefore, that they might not have focused more, as originally assumed.

A problem with the foregoing argument is that attention simply need not improve performance. This is a central tenet in Kahneman's (1973) classic "Attention and Effort," and it has been confirmed innumerable times since. The reason society devotes so much effort to improving the design of cockpits and roads and complicated documents is precisely because attention often does not bring about better performance. (And, conversely, in cases of expertise, improvement can be achieved with no greater attention.)

The other problem with this argument is substantial research, by several authors, in good journals. "We have presented evidence here," the authors continue, "that the behavioral differences Shah and colleagues observed between 'rich' and 'poor' participants in their study did not arise from attentional differences but instead from elements of the game's structure." There are an increasing number of studies showing clear attentional differences – sometimes focus, sometimes neglect -- between rich and poor (experimental participants and real people) that cannot be attributed to any game's structure.

Older research in marketing has tracked the food shopping strategies of low-income consumers and found that income significantly influenced price recall, suggesting lower-income shoppers were more attentive to and knowledgeable about prices (e.g., Wakefield & Inman,1993; Estelami & Lehmann, 2001). Steenhuis et al. (2011) find that "Low-income consumers were significantly more conscious of value and price than higher-income consumers." Spiller (2011) finds that the poor engage in more tradeoff thinking. Ellwood-Lowe, et al. (2022) find that caregivers who are prompted to reflect on financial scarcity speak less to their 3-year-olds in a play session, relative to a control group. Similarly, caregivers engage in fewer back-and-forth exchanges with their children at end of the month—when there's more financial hardship—than the rest of the month. Duquennois (2022) observes that lower SES students show evidence of depressed performance on monetary questions, compared to numerical questions that are virtually identical. Meuris & Leana (2018) report that worry about money made truck drivers less attentive on the job. Kaur et al. (2025) found that financially constrained workers, when they had less cash on hand, worked slower and less attentively, and made more mistakes. Speaking of different mindsets of rich and poor, according to Huijsmans et al. (2019), "neuroimaging results suggest that a scarcity mindset affects neural mechanisms underlying goal-directed decision making."

One repeatedly puzzling claim is that the observed behavioral differences between 'rich' and 'poor' participants can be "more parsimoniously explained by the game's structure." In fact, the game's structure is the *only* difference leading to differential behaviors between the 'poor' and the 'rich.' The only serious, implicit question is whether that structure can explain the observed differences without any recourse to their effect on participants' mindsets. It's not clear whether that's what the current authors' believe -- everything we know from many other studies is that mindsets do tend to differ. But the conclusion that "we do not present any evidence against..; rather, we highlight the lack of evidence in support" is not terribly informative and strangely limited to this one study they are critiquing.

The "additional analyses" focusing only on those cases in which participants had the opportunity to borrow are truly puzzling. They compare participants who have no shots left with participants who have no shots left. Or to be more precise, they compare participants who have no shots left with participants who have no shots left but seconds ago had many (and apparently still keep on shooting..). Imagine a study of the habits of the rich and poor, where the rich are people who were born rich and now have nothing. If you did that, you would find, contrary to popular expectation, that both the rich and the poor sleep under bridges, go hungry, etc. This really makes no sense.

Similarly, the insurance study, being entirely orthogonal to the issues at hand, really sheds no light. It observes no difference for insurance with positive expected value, and it observes the poor to be more discerning for scam insurance. Shah et al. really don't seem to have anything precise to say, consistent or inconsistent with these patterns. The poor may be relatively neglectful of the positive insurance (which standard accounts say they should take more than the rich), or they might be attending to the scam insurance, which is highly financially threatening, and is where the poor are presumed to focus more. Like the original authors, the present authors have no clear predictions either, and both observed patterns, incidentally, contradict what happens between rich and poor in the real world.

So what, finally, does the paper provide? It argues, free of evidence, that a lack of improvement could be consistent with no greater focus. It establishes that there's a lack of significant correlation between aiming times and higher borrowing rates, an analysis that, as pointed out, Shah et al. reported failing to replicate several years ago. It provides a truly puzzling analysis where participants with a zero budget are called rich and found to behave like the poor, and it adds an "insurance study," which finds results that are inconsistent with what we observe in the real world, not predicted by the present authors, nor confirming or contradicting any clear prediction by the original authors. This is not a paper that will help a general readership better understand the issues at hand.

(Remarks on code availability)

Reviewer #4

(Remarks to the Author)
I am happy with the authors' responses to my comments, and I continue to strongly support the paper. I have just a few notes on the responses.

1) MTurk, sample sizes, and data quality: My point was that MTurk data quality is poor; therefore, I would have preferred a larger sample, despite the power analysis. The added sanity checks (now more prominently described) are reassuring. So

that's fine. The argument for using the same platform as the original is less convincing; it also applies to the current setup, i.e. observed differences could be attributed to unobserved effects over time.

2) Re-analysis of Studies 3-5: I am glad that the authors now also re-analyzed the other studies and that the results align with my prior. Although I am a bit surprised the original study's data is no longer available online (nor obtainable from the authors?). Since Study 3 cannot be re-analyzed, the manuscript and supplemental material should not claim that "Studies 3 to 5" were re-analyzed or that the critique of Study 2 "also applies to Studies 3 through 5." Such claims can only be made for Studies 4 and 5.

3) Non-independence of shots: My point is that the second (and third) shots are not independent observations, and the analysis should in some way account for it. An easy fix would be to average the three shots within the poor and rich conditions and compare those averages and only then analyze shots independently (of course, one could do more sophisticated stuff). Other than noted in the author's response, I do not see this analysis in the manuscript or supplemental material.

4) Additional measures, pre-registration: The authors clarify the issue about the additional measures. The pre-registration lists two additional hypotheses (H5 and H6), but they are not reported anywhere (unless I missed it). I think one should always follow through with the plan and report all pre-specified analyses somewhere. What ends up in the paper is a different story (only a few people can envision their complete paper before they have seen the data).

(Remarks on code availability)

Reviewer #5

(Remarks to the Author)
Thank you to the authors for the careful revisions and the additional interpretive clarity. The expanded discussion of equivalence tests in both the main text and supplement substantially improves readability, especially for readers less familiar with the method. I also appreciate the addition of alternative SESOI and the sensitivity analyses—this makes the manuscript more transparent and flexible.

I do want to touch upon two points, which I believe should be addressed to make the equivalence tests effective:
1. Substantive justification of the SESOI.
I understand the challenge of defining a real-world benchmark in an artificial task, and your decision to rely on the 33% power rule is methodologically defensible. That said, I still encourage you to consider at least a provisional domain-based rationale (e.g., performance differences that would matter for decision-making, or even qualitative stakeholder perspectives). Even a tentative justification would help anchor the SESOI in substantive meaning rather than leaving it entirely at a statistical convention. Without this, the "so what?" of equivalence remains harder for readers to evaluate.
2. Terminology of conclusions.
In a few places the manuscript equates equivalence with "no difference." I would suggest instead framing this as "negligible difference" or "no meaningful difference". The distinction matters because the SESOI defines what counts as negligible; differences outside it could still be meaningful (and conversely, non-zero differences that one finds are trivial as long as they are within the bounds). This refinement would better communicate the logic of equivalence testing.

Overall, the revisions have strengthened the paper, and addressing these two points would further align the manuscript with best practices in equivalence testing and improve the interpretability of your conclusions.

Thank you for a chance to read the manuscript and I wish the authors the best of luck in this interesting work!

(Remarks on code availability)
No different comments than before.

Open Access This Peer Review File is licensed under a Creative Commons Attribution 4.0 International License, which permits use, sharing, adaptation, distribution and reproduction in any medium or format, as long as you give appropriate credit to the original author(s) and the source, provide a link to the Creative Commons license, and indicate if changes were made.
In cases where reviewers are anonymous, credit should be given to 'Anonymous Referee' and the source.
The images or other third party material in this Peer Review File are included in the article's Creative Commons license, unless indicated otherwise in a credit line to the material. If material is not included in the article's Creative Commons license and your intended use is not permitted by statutory regulation or exceeds the permitted use, you will need to obtain permission directly from the copyright holder.
To view a copy of this license, visit https://creativecommons.org/licenses/by/4.0/

Answer letter to “How does scarcity impact attention? A critical examination of the tunneling hypothesis’”

Fiona tho Pesch, Antonia F. Langenhoff, and Mahesh Srinivasan

Note by authors: Please find the reviewers’ comments in normal font, and our answers to the reviewers’ comments in bold below.

Reviewer #1 (Remarks to the Author):

First of all, I’d like to thank the authors for this interesting submission. The main topic of this paper—examining the verisimilitude of the tunneling hypothesis and attentional neglect in the context of scarcity, through robustness testing of the original data and a conceptual replication using a new dataset—is highly relevant to a broad readership. There are many positives to the paper. Besides the relevance of the topic, it presents results that mostly contradict the original findings. I find publishing contradictory/null results extremely important for the progress of the field. I appreciate how the authors complement their findings with pragmatic explanations, rather than over-psychologizing them. The writing is mostly clear and easy to understand. As a strong proponent of open science practices, I particularly appreciate the authors’ adherence to these principles.

However, this is one of those papers that left me with mixed thoughts about how to evaluate it. Although I’m very sympathetic to the authors’ efforts and find their explanations much more plausible compared to the original claims, I’m still skeptical about the findings because of two main factors: (1) ecological and external validity (far-transfer) and (2) data quality. Given the design of the study (i.e., data re-analysis and conceptual replication), both are at least partly outside the authors’ control. Much of my criticism is, therefore, directed toward the validity of the original claims. I’ll elaborate on these points below and include a few comments regarding the methodology used. I hope the authors will find my feedback useful.

R1A1: Thank you for your kind words. We fully agree that both the original study and our conceptual replication have several limitations. However, the central objective of our manuscript is to highlight the lack of convincing empirical evidence supporting the tunneling hypothesis. Given that Shah et al.'s (2012) paper has been widely regarded as the primary, direct evidence for this hypothesis, our contribution is to demonstrate that their paradigm does not provide the necessary support for these claims. Importantly, we show that even if one were to accept the argument that such artificial experiments could simulate the real-world experience of poverty (which we also question), the data still do not support the original conclusions. We have now

added a couple of sentences on this topic to the introduction ("Generally speaking, experimental manipulations of scarcity can be criticized for not fully capturing the complexity of experiencing poverty (i.e. low external validity). Still, research on poverty often takes this approach, as manipulating poverty in real-world settings can be ethically and practically unfeasible. Importantly, even if the study of Shah et al. was assumed to reflect real-world conditions, our reanalysis demonstrates that the original claims do not hold up." (p. 4))

Ecological and external validity:

Nicely illustrated in the first two sentences of the abstract, the main goal of the tunneling hypothesis is to explain the psychological mechanisms behind poverty perpetuation. My main concern is that the experimental situation used in this study is, at best, a proxy for real-world scarcity settings, and it is unclear to what extent these findings can be transferred to the real world, which is far more complex.

In the "Angry Blueberries" game and similar tasks, participants operate in an artificial environment that is safe, has clear rules, and offers limited actions. The consequences of participants' decisions (e.g., shots, borrowing, insurance) are straightforward and apply only to the specific task for a limited time, unlike the complex decisions individuals in poverty face in their everyday lives. Real-world decisions involve more emotional (e.g., shame, frustration, anxiety), social (e.g., family needs, marginalization), and environmental factors (e.g., employment, healthcare, unexpected events) than those typically captured in a lab setting. Particularly in the context of scarcity, ongoing financial instability, cognitive load, social pressures, and systemic barriers to accessing resources can dramatically alter how people make decisions, thus creating mechanisms that may not be fully replicated in a simplified task.

A major role is likely also played by temporal factors. While real-world scarcity typically involves sustained, long-term lack of resources and opportunities, these simulated tasks and experimental manipulations of resource availability last only a few minutes. Participants can withdraw at any time, and the tasks have no real-life consequences. Neglecting these factors may result in discovering oversimplified patterns, rather than understanding the true behavioral mechanisms behind poverty perpetuation.

Again, I'd like to emphasize that I fully agree with the authors' reasoning that attributes the observed decision-making of the participants to the game design rather than to the psychological mechanisms behind the conditions of scarcity. I've been considering how this can be improved in the paper—my suggestion would be to put more focus on the limitations of the experimental task used and potentially further investigate whether the current findings hold across different games and scenarios. This will be a very beneficial and essential step before attempting to explore how to test the verisimilitude of the hypothesis under real-world circumstances.

R1A2: We agree with Reviewer 1 that the artificial and short-lived experimental manipulation of scarcity likely does a poor job of capturing the real-world experience of poverty, and we have added content to the revised manuscript to note these concerns about external validity. However, we also recognize–just as the reviewer

does–that lab experiments provide a unique means of establishing causality, and given the prominence of Shah et al.'s (2012) original paper, many researchers seem to believe that these experimental paradigms offer insights into real-world conditions. Importantly, our manuscript demonstrates that even if one were to accept that this experimental paradigm successfully induces a sense of scarcity, the data still do not support the tunneling hypothesis. Thus, our re-analysis does not rely on critiquing the experimental manipulation itself but shows that, regardless of whether one accepts the ecological validity of the manipulation, the data do not substantiate the original claims. We have added a short paragraph on this issue within the introduction (see R1A1). We would be happy to expand on general concerns with the approach of studying poverty through experimental manipulations of scarcity if desired, but because it is not the focus of our paper, we have kept this discussion brief.

Similarly, as our primary aim is to evaluate the strength of existing evidence for the tunneling hypothesis, conducting additional experiments with varied setups to validate it falls beyond the scope of our paper, though we now highlight the need for such efforts in the Discussion.

Data quality:

My second major concern is the quality of the data. Both the authors of the original study and the authors of the current study recruited participants using Amazon MTurk. MTurk is notoriously known for issues related to insufficient data quality (e.g., Chmielewski et al., 2019; Douglas et al., 2023; Webb & Tangney, 2022).

R1A3: While we agree with Reviewer 1 that the data quality of Amazon MTurk can be critiqued, the main goal of our manuscript is to show that show that Shah et al.'s (2012) study does not provide any evidence in support of the tunneling hypothesis. To do so, we kept the study protocol of our conceptual replication as close to the original study as possible. Since the original study was conducted online on MTurk, we decided to also conduct our conceptual replication on MTurk.

Regardless of the potential issues with MTurk data quality, there are other factors that might be affecting the results. The authors of the present study addressed careless responding by incorporating three comprehension checks after the instructions (I found no information about handling careless responding in the original paper). However, almost no further information about the participants and potentially relevant variables is provided. I assume that participants were randomly assigned to the experimental condition (poor vs. rich). However, with relatively modest sample sizes (N = 68 and N = 174; note that the authors of the current study also re-analyzed a self-replication of the original claim but have kept these results in the supplementary material), random assignment may not fully account for individual differences that could affect performance and decision-making. Variables such as previous experience with similar games, stress levels, cognitive abilities, or enjoyment of the task could introduce variability that the study does not control for. Measuring and accounting for these factors would provide stronger evidence for or against the tunneling hypothesis, as it would help isolate the psychological mechanisms at play and explore the influence of alternative explanations.

R1A3: We agree that the original publication should have accounted for individual differences. As Reviewer 1 points out, given the small sample size of the original study (Shah et al., 2012), random assignment might not fully account for individual differences. However, the large scale replication with 1010 participants yields essentially the same results, with very similar means on the most important parameters. We agree that if one were to argue in favor of the tunneling hypothesis, it would be essential to prove that any observed effects are due to tunneling itself, rather than factors like enjoyment, cognitive abilities, or other individual differences. Our objective, however, is not to argue in favor of or falsify the tunneling hypothesis, but rather to demonstrate that there is no empirical evidence supporting it from Shah et al's study, contrary to current perceptions within academia and beyond.

Following this, the authors state that they excluded data from participants who did not finish playing the game. Not finishing the game could indicate several possibilities: a participant may have found the game too difficult or frustrating (especially in the "poor" group), perhaps their performance didn't meet their expectations, or maybe the game was boring, too easy, etc. Essentially, this presents a missing data problem, and the reasons for not finishing the task are likely unknown. Excluding these participants might introduce bias stemming from conditioning on a collider (experimental condition -> finishing the game <- performance). Therefore, taking a closer look at these participants and running a sensitivity analysis on all available data might reveal slightly different patterns. Lastly, in their preregistration, the authors state that they aimed to collect data from at least 200 participants. Since they report no deviation from the preregistered protocol, the effective sample of N = 174 likely indicates that at least 13% of the original sample either failed the comprehension checks or did not finish the game.

R1A4: Regarding the issue of missing data and the exclusion of participants who did not finish the game, we agree that this is a potential concern. Unfortunately, we do not have information on potential dropouts, as the "Angry blueberries" game only recorded complete datasets. We received the base program to the game from the original authors, and as such we suspect that their data face the same limitation. However, the exclusions within our conceptual replication study followed the same approach as the original study, meaning that we only recorded data from participants who finished the whole game. While participant dropouts may introduce bias, this limitation stems from the original study design rather than our re-analysis. As our goal is to demonstrate that Shah et al. (2012) do not provide any evidence in favor of their claims, we do not think that this kind of bias is contradicting or interfering with our main conclusions. Nevertheless, we have added a sentence about this to our methods section: "Please note that we have no information on participant dropout within the original publication (Shah et al., 2012). In their large-scale replication, the authors note that participants were excluded from the analyses if they were missing subject ID numbers or if an error in the game led to an incorrect allotment of blueberries, such as if the game ended prematurely before they could use their blueberries or allowed them to continue playing after their blueberries were exhausted. After applying these exclusion criteria, 36 participants were removed, resulting in a final sample of 974 participants for the analyses. Furthermore, we have no further information on the participants who dropped out, such as demographics, in the original study. Therefore, we cannot rule out potential bias due to systematic

dropouts or incomplete randomization in that context. This issue also applies to our conceptual replication study, as we used the same program for the Angry Blueberries game, which did not retain information on dropouts." (p. 20).

Regarding the preregistration, we appreciate Reviewer 1 pointing out the inconsistencies. We acknowledge that our manuscript did not give the conceptual replication study the level of detail it deserved. We have now added a section titled "Deviations from Pre-registration," (p. 36) where we fully disclose and explain any inconsistencies, including the fact that our final sample size was slightly smaller than the intended 200 participants due to budgetary constraints.

I also wonder how the authors dealt with surprisingly fast or slow shots or other potentially peculiar patterns (e.g., always borrowing). I understand they log-transformed the reaction times, but based on my quick glance at the data, I believe this issue deserves more attention (any substantial data cleaning should be reported explicitly) and ideally should be addressed through a sensitivity analysis.

R1A5: Once again, we agree with Reviewer 1. However, as with the previous concerns, this criticism is primarily directed at the original experimental setup and the way the original authors handled the data. Since our aim is simply to show that Shah et al.'s (2012) study does not provide any evidence in support of the tunneling hypothesis, our ability to modify the original data analysis is limited. We do provide more clarity as to how the data was treated by the original authors in the introduction ("All reaction times (i.e. aiming times) were log-transformed, probably to normalize the data, reducing skewness and stabilizing variance, making it better suited for statistical analysis.", p. 5). Since we found no indication of data treatment by the original authors based on unexpected patterns in the results (like always borrowing or surprising aiming times), we likewise did not exclude any observations on this basis, to adhere as closely as possible to how they treated their data.

Minor comments:

I'd suggest adding more clarity to the aims of the study and the corresponding design. Although they are mentioned throughout the text or implied, summarizing them would help readers better navigate the study. Additionally, mentioning the re-analysis of the self-replication by Shah et al. (2019) in the main text might provide a richer context regarding the stability of the claims.

R1A4: Thanks for this recommendation. We added a sentence in the introduction, making the aim of our manuscript clearer ("Note that we do not present any evidence against the tunneling hypothesis; rather, we highlight the lack of evidence supporting it.", p. 4). We furthermore added information on our replication of all analyses in the large-scale replication of Shah et al. (2019) in the introduction ("Our re-analysis reproduces these effects, both in the original dataset, as well as in the large-scale replication from 2019.", p. 4).

The authors correctly decided to utilize equivalence testing to determine whether the outcomes of the experimental conditions are practically equivalent (i.e., whether the effect is

within the equivalence bounds). However, they set their SESOI based on statistical criteria (i.e., an effect that the original study had a 33% power to detect) rather than a theoretical or practical justification. To move beyond simply re-analyzing and replicating the original findings, I'd suggest justifying the effect size based on its theoretical or practical significance. Additionally, the authors write that "Equivalence testing revealed that the study was underpowered to support the conclusion that the 'poor' and the 'rich' were statistically equivalent." Can the authors clarify this in the main text? It is one of the focal claims, and without additional context (though partly available in the supplementary materials), it is difficult to determine what the SESOI was and to interpret whether the confidence interval was too wide and/or outside the predetermined equivalence region.
Related to the above, it would be beneficial to present a power curve for hypothetical sets of effect sizes and sample sizes, rather than relying solely on point estimates from the power analysis. This would be especially helpful given that the SESOI is somewhat arbitrary, and the study used N = 174 instead of the preregistered N = 200, on which the original power analysis was calculated.

R1A5: In determining the smallest effect size of interest (SESOI), we followed the guidelines of Lakens et al. (2018). While we agree that a theoretically or practically informed effect size would be preferable, the artificial nature of the task makes it difficult to establish a meaningful effect size based on theoretical or practical significance. As a result, we opted for a benchmark approach, which we believe is the most informative option in this case. However, we understand the limitations of this approach and appreciate the reviewer's suggestion. We acknowledge these limitations now within the supplemental materials: "While we acknowledge the limitations of basing the SESOI on a statistical criterion, the artificial nature of the experimental design does not allow for a practical justification of it." (p. 29).

Regarding Reviewer 2's suggestion to include more information on the power to detect certain effects, we added an additional section on this within our supplemental materials on page 31:
"Power curve for hypothetical sets of effect sizes and sample sizes
To gain a better understanding of the power achieved over the three different studies reported on in our manuscript, we plotted a power curve for hypothetical sets of effect sizes and sample sizes (see Figure S1). Additional sensitivity analyses taking into account the distribution of participant over different conditions indicate that the original study had 80% power to detect large effect sizes of up to d = .619. Our replication study had 80% power to find effect sizes of up to d = .379. Crucially, the large-scale replication of Shah et al. had 80% power to detect very small effect sizes of up to d = .161.
Figure S1
Power curve for different effect sizes.

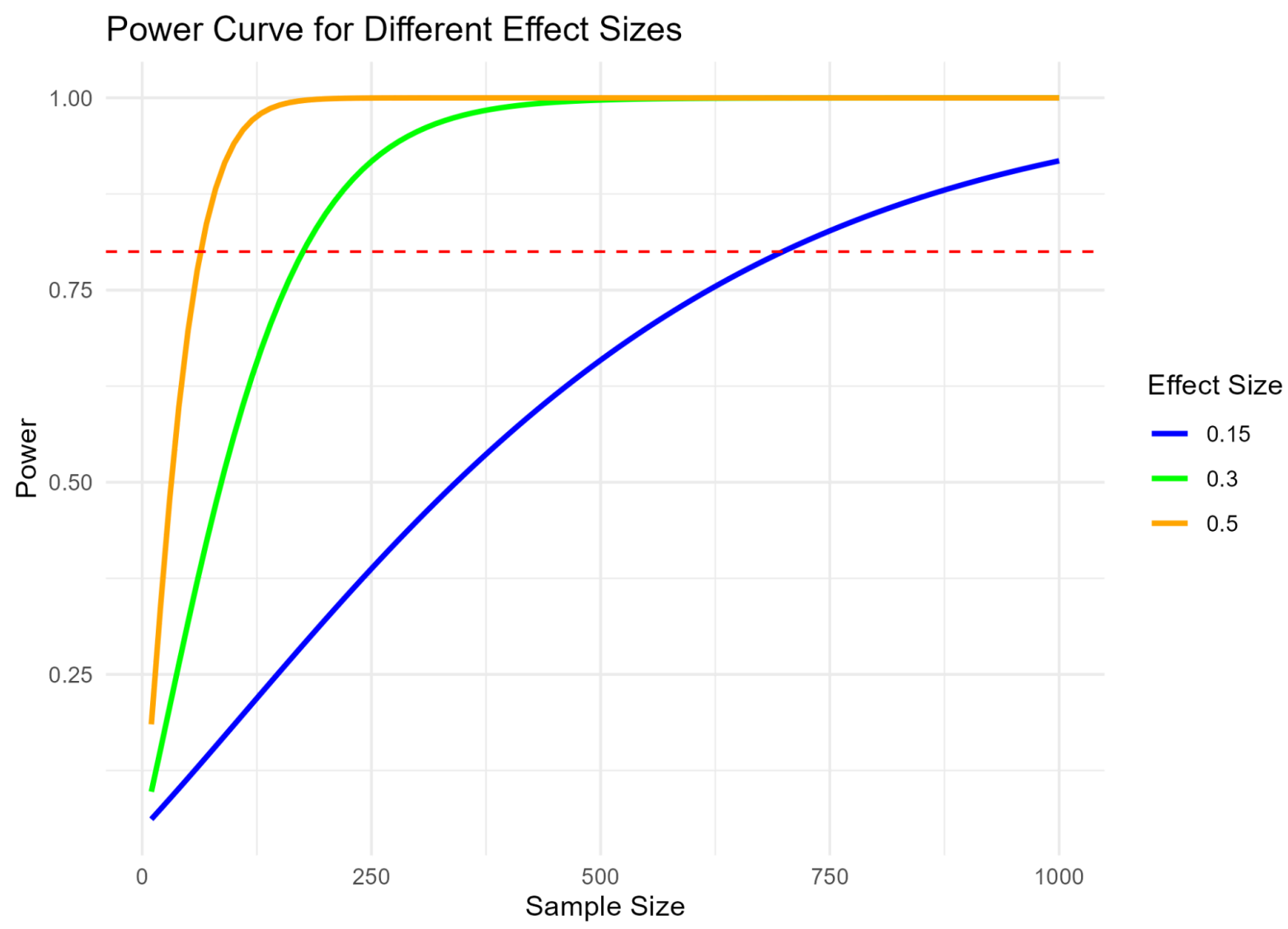

Furthermore, given that the SESOI we used is somewhat arbitrary, we explored different SESOI values to test equivalence in performance on shot 3, which showed numerically the largest (non-significant) difference between conditions in the large-scale replication of Shah et al. (2019) with *d* = 0.064. As shown in Figure S2, even with an SESOI of 0.2, the study is well-powered to detect equivalence.

Figure S2
Power curve for Two One-Sided Tests (TOST) equivalence test displaying different SESOIs

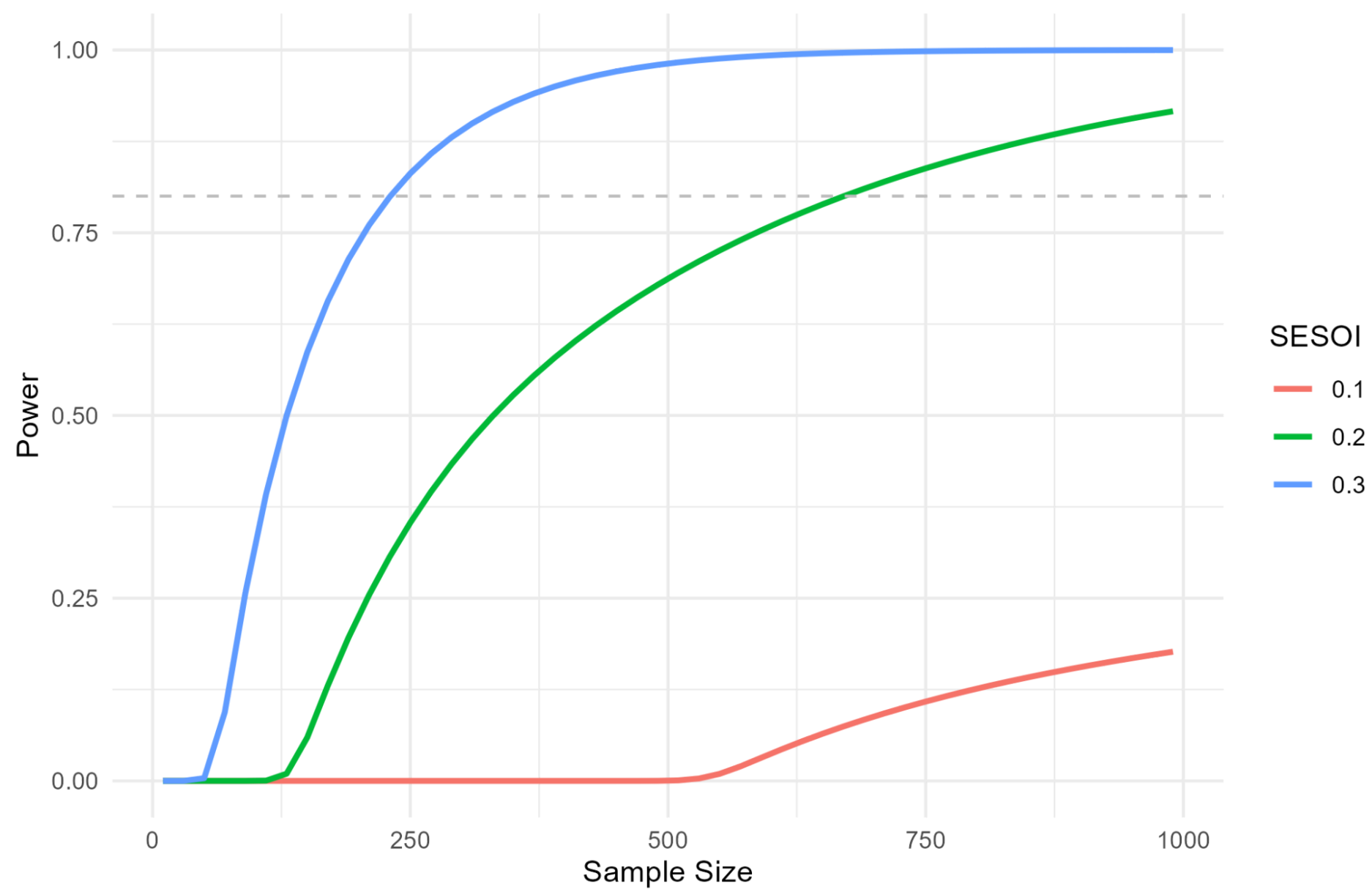

" (p. 30).

Regarding the clarification of our equivalence testing, we agree that our manuscript could have provided more context. We have now included additional details in the

main text to explain the SESOI we used and to clarify the interpretation of the results, particularly regarding the confidence intervals and the equivalence bounds (see p. 9).

I checked the supplementary materials on OSF. Kudos for sharing everything, including materials and instructions—that's how transparent and responsible research should be! I noticed that the data collection for the conceptual replication also involved other experimental tasks as well as additional measures (https://osf.io/wf57c?view_only=19edfe9ed67f40009b677fb11c8c60f8). In the informed consent, the authors explicitly mention that participants would be asked to complete several tasks, which would take around 120 minutes. I fully understand that it's common practice to collect more data at the same time. However, I wonder—could this have affected data quality and participants' decision-making? Completing multiple experimental tasks and filling out questionnaires might become exhausting after a certain point. If this supplementary material information is correct, did the authors account for this, perhaps by randomizing the order of tasks? Furthermore, I noticed that the preregistration includes additional analyses (e.g., mediation through time and risk preferences), which are not mentioned in the paper. Just to clarify—were only some parts of the preregistered analyses presented in the paper, or am I missing something? Were there any other (substantial) deviations from the preregistration that should be disclosed in the paper?

R1A6: We apologize for this misunderstanding. Indeed, we did not include any other studies or additional tasks within this data collection (the reviewer is referring to an informed consent form that outlines the range of measures that a study in the lab could in principle involve–but these were not presented to participants of the conceptual replication study). Participants only took part in the task outlined in the conceptual replication study, and all materials and procedures are fully disclosed. For further details on any deviations from the pre-registration, please refer to the section titled "Deviations from Pre-registration" (p. 36).

Overall comments and suggestions:

As much as I'm sympathetic to the authors' efforts and think that their pragmatic explanations of the tunneling hypothesis are valid, a more rigorous continuation of the current lab-based approach is needed to address the research question more thoroughly. Since collecting data in real-world settings would be too complex, potentially unethical, and likely too far removed from the authors' aims, continuing with controlled lab-based tasks is the most appropriate path forward. To build on this, I suggest conducting a multiverse analysis, varying and synthesizing multiple reasonable data cleaning and hypothesis-testing possibilities. Additionally, expanding the experimental scenarios—ideally with a within-person design that varies levels of scarcity throughout the game—could offer further valuable insights. Incorporating more relevant variables into the experimental design and analyzed models could also shed further light on the behavioral mechanisms underlying decision-making in the context of scarcity.

Again, I'd like to express that I'm aware much of the above criticism is related to the original claims. However, I'd like to see results that are robust enough to convince the field and allow researchers to confidently build upon them, helping the field move forward.

I hope this feedback helps.

All the best.

R1A7: Thank you for these interesting ideas. We agree that claims about the effects of scarcity on behavior should ideally be supported by within-person designs and multiple experimental approaches, and we have now included a statement in our revision on this within the discussion section ("It is crucial that any experimental manipulation rigorously ensures that observed effects genuinely stem from shifts in mindset, rather than from alterations within the decision space. To achieve this, employing multiple experimental methodologies including within-subject designs may be useful.", p. 19)

In response to Reviewer 1's suggestion to explore alternative methods for data analysis, we agree that this approach offers valuable insights into the extent to which the data supports a given hypothesis. We believe our re-analysis demonstrates the value of this approach, as we show that the original authors' claims are not supported by their own data.

Overall, we would like to thank Reviewer 1 for their thorough reading of our manuscript and for the attention given to our OSF repository. The feedback was very helpful and has significantly improved the manuscript.

Reviewer #1 (Remarks on code availability):

I reviewed the code briefly, focusing on the parts written in R, as I'm not very familiar with Stata syntax, where most of the analyses and data cleaning were performed. From what I can tell, the code looks alright.

Reviewer #2 (Remarks to the Author):

I very much enjoyed engaging with this re-look at a seminal finding in the psychological study of poverty, a finding that has formed the foundation of a set of strong theoretical assumptions, most prominent of which is the claim that scarcity leads to 'tunneling'. It has long bothered me that the mechanistic assumptions made by the team who published the original set of studies on this have not been adequately scrutinized, preventing the opportunity for a subfield of the study of scarcity/poverty to develop in a theoretically precise and robust manner. Absent this strong theoretical footing, the recent failures to replicate some aspects of this work have cast a shadow on the field more generally, despite it being a promising and important one to advance. This matters not only for the health of a nascent subfield cutting across psychology, behavioral economics, and social policy, but also for the wider impact of the work, as the notion of a 'scarcity mindset' is now widely discussed in policy circles, in designing interventions for NGOs combatting poverty, and indeed in the plethora of self-help videos claiming to help people escape such a mindset. It is thus of critical importance, for the social and behavioral sciences and for the public debates that they inform, that the scientific base is as clearly and precisely understood.

The current paper does this critical work. It takes the early theoretical claims of what has become known as ‘scarcity theory’ at face value, digging into their implications, and then adjudicating on whether or not their empirical base is strong enough to warrant the claim that scarcity leads to a circumscribed form of enhanced performance due to greater focus, at the same time as suboptimal, present-oriented decisions due to attentional neglect. The clever reanalysis of the core findings supporting these claims is thoroughly convincing as to the need to reconsider both conclusions. Ideally, this would be the kind of analysis that is conducted in response to attentive reviewers of the original paper, but it is never too late for that work to be done! The additional analysis of the 2019 paper, in addition to the current authors’ own conceptual replication, makes a strong package worthy of publication in a top pan-science journal such as Nature Communications.

I have very little feedback to offer as I found the writing to be clear and concise, the analyses to be appropriate and the reporting transparent and comprehensive. I did find I wanted to hear more from the authors about the implications of their argument, in the discussion, that much of what the poor face are not a case of decision-making constrained by a particular mindset that differs from that of the rich, but decision-making constrained within a different decision space or set of parameters than that of the rich. This basic point about the importance of structural constraints on decision-making has been neglected by the field, and is neatly demonstrated in this re-presentation of the parameters of the Angry Blueberries game. Perhaps another few lines on what implications this might have for how to research scarcity/poverty going forward would help increase the impact of the paper.

R2A1: Thank you for the positive review, and for the suggestion to add a bit more on the interpretation of our re-analysis for the field of researching the psychology of poverty. We added a couple more sentences on this within our discussion (“Even though the “Angry Blueberries” experiment of Shah et al. does not provide evidence for the tunneling hypothesis, it might mirror important structural effects that scarcity has on people. The environments we inhabit differ depending on one’s SES, and different structural factors of these environments shape our behavior. From this perspective, much of what the poor face may not simply be a case of decision-making shaped by a unique mindset; rather, it is constrained within a fundamentally different set of parameters or decision space than that of the rich. For example, in Shah and colleagues' study, as well as in the real world, the 'poor' face the decision of whether to borrow at inflated interest rates more often than the 'rich'. As such, the experiment tells us something about how having the option to borrow differentially affects the 'poor' and the 'rich' (even though this disparity cannot be attributed to the proposed attentional mechanism of tunneling). Importantly, this effectively illustrates the importance of considering structural constraints when studying decision-making in contexts of adversity—an aspect largely neglected by the field. Moving forward, researchers could examine these structural constraints and how they shape behavior differently depending on an individual's position within the social hierarchy.”, p. 19).

One place where I felt more clarity could be offered was on lines 497 to 500 on p.90, in the supplementals, where it appears the authors say it should be equivalent, then that this result implies it’s not equivalent, then say therefore it was underpowered…? The logic of how this

particular finding from an equivalence test implies that a test is underpowered, as opposed to evidence against the authors' prediction, could be spelled out more clearly.

R2A2: Thank you for raising this question. Indeed, if one is unable to find evidence for a difference between two groups (as shown by the regular ANOVA), and is also unable to find evidence for equivalence between the two groups (as shown by the subsequent equivalence test), the conclusion is that the study was underpowered to make any definitive claims (see Laakens et al., 2018). To clarify this, we have added a sentence to explain the reasoning more explicitly: "As both the condition factor within the ANOVA models and the equivalence tests were insignificant, this suggests that the study was underpowered to draw any conclusions regarding whether there were differences in performance between the 'poor' and the 'rich' on the first three shots of each level." (p. 29).

I also caught the following minor grammatical issues/typos:
- Gamma footnote – repetition in saying '80% power…with a power of .8'
- Line 487 p.27 typo 'wohle'
- Line 498 p. 90 'did not reveal statistical equivalence…on neither the first shot…nor the second…' – should be 'on either the first shot…or the second' (or 'revealed statistical equivalence…on neither the first shot…nor the second'
- Line 557 p.31 'significantly interaction effect'

R2A3: Thanks for pointing out our typos, which we have corrected.

All in all I was impressed with this paper and convinced that its publication would be a highly important contribution to the field, even if it challenges some core assumptions of thinking in that field…including that of my own!

Jennifer Sheehy-Skeffington.

Reviewer #3 (Remarks to the Author):

The paper presents some re-analyses and re-interpretations of one study in Shah et al.'s multi-study 2012 paper, entitled "Some consequences of having too little." The original analyses are successfully replicated; the authors then present "additional analyses" to argue against some of the interpretations offered by the original authors. The critique mostly concerns the original authors' proposed explanations concerning participants' attention allocation, whereas the additional analyses "suggest instead that the observed behavioral differences between 'rich' and 'poor' participants can be more parsimoniously explained by the game's structure."

The distinction is hard to interpret. Since participants in the original studies were randomly allocated to "rich" and "poor," determined solely by the game's structure, it seems clear that it is, in fact, the game's structure that leads to the observed differences. How much of the data is attributable to what behavioral factors remains to be better understood. But since there are

no actual “rich” and “poor” participants in the study, only participants made rich or poor by the budgets allocated to them by the game, the observed behavioral differences are bound to be explained by the game’s structure.

R3A1: Thank you for raising this point. We agree, of course, that the game’s structure defines who is “poor” and “rich.” However, the main argument of the original authors is that the experience of scarcity changes how people allocate their attention, which then leads to differences in behavioral outcomes. This is reflected in Shah et al.'s summary statement: “Taken together, these studies provide compelling support for the notion that scarcity elicits greater engagement and that a focus on some problems leads to neglect of others (manifesting in behaviors such as overborrowing).” If the original paper had simply stated that “rich” participants with more shots performed worse in terms of average points-per-shot and borrowed less often, we would have had no concerns. However, the original authors go further by claiming that the experience of scarcity (i.e., the experimental manipulation) alters behavior due to attentional differences between the two experimental groups (i.e., with scarcity leading to greater engagement causing higher points-per-shot among “poor” participants, and attentional neglect leading to increased borrowing among “poor” participants). Thus, the original paper goes beyond merely reporting behavioral differences and infers cognitive mechanisms—specifically attentional shifts—as driving the observed behavioral differences. Throughout our revised manuscript, we have tried to clearly distinguish between the behavioral effects of the manipulation, and the effects on attention. To this end, we have also added another sentence on this within the discussion: “We do not contest the effect of the manipulation on behavioral outcomes (such as improved performance and increased borrowing among the 'poor'); however, we suggest that these outcomes are driven more broadly by the structure of the game itself, rather than by a shift in attention.” (p. 15).

In our manuscript, we contest this notion that the manipulation affects attention, leading to the observed behavioral effects. Instead, we demonstrate that the behavioral effects directly stem from the game’s structure without altering participants' attention. Importantly, by changing the budget of participants, the experimental setup not only impacts the mindset of participants, but also puts very real constraints on behavior. For example, “rich” participants can only borrow once they exhausted their budget of 15 shots, meaning that a large fraction of the “rich” participants did not have the option to borrow on most of their levels, because they cleared all targets with these 15 shots. Our argument is that these real constraints on behavior are better explanations for the observed behavioral effects, rather than a shift in attention.

The authors seem to think that the need of “poor” participants to borrow more often than the rich is somehow a problem, but, again, that appears to be in the nature of the study’s design. Borrowing at very high interest in the original game was designed to be potentially harmful (as it is in life). The finding, apparently, is that being poor more easily leads one to take expensive loans, to their own detriment, whereas being rich allows one more easily to avoid it. As a result, the studies find that the poor, when they can borrow, do worse than when they cannot, contrary to the rich.

R3A2: We agree with Reviewer 3, as we also state in our discussion: “In Shah and colleagues' study, as well as in the real world, the 'poor' face the decision of whether to borrow at inflated interest rates more often than the 'rich'.” (now p. 19). The results of Shah and colleagues certainly highlight how the option to borrow affects the “poor” and the “rich” differently. However, as we also noted in the next sentence of the discussion: “As such, the experiment tells us something about how having the option to borrow differentially affects the 'poor' and the 'rich' (even though this disparity cannot be attributed to the proposed attentional mechanism of tunneling).” (p. 19) Unfortunately, the reviewer seems to overlook this central point of our argument. It is not surprising that the more often a behavior like borrowing is available as an option, the more often people will engage in it. What makes the claims of Shah et al. newsworthy–and what our paper takes issue with–is the psychological claim that scarcity itself may increase susceptibility to poor decision-making (in this case, overborrowing) via attentional shifts.

We have now added a new paragraph addressing this issue, following the recommendation of Reviewer 2 (see R2A1).

The authors write, “our additional analyses show that when examining only those cases in which participants had the opportunity to borrow, 'rich' participants always borrowed, while 'poor' participants only borrowed in 69% of cases.” That is certainly false. Both rich and poor participants in the borrowing conditions were allowed to borrow throughout. What the authors might mean is not “the opportunity to borrow” (which both “rich” and “poor” have throughout the borrowing condition) but “having gotten to the point where one needs to borrow.” That is a very different thing than having the opportunity to borrow, and here the comparison seems strange: A “rich” participant who needs to borrow if they want to continue has no budget left – they are now “poor.” Again, if I understand correctly, there is nothing “poor” or “rich” about the participants, other than the budget they have. A participant who decides to borrow with noting left is a poor participant borrowing.

R3A3: Thank you for bringing this up. We believe the reviewer has a misunderstanding of the original experimental setup. Participants could only borrow once they had run out of shots on a given level. It was technically impossible to borrow while they still had shots left in their budget for that level. In other words, even if a participant wanted to borrow shots from future rounds while still having shots left, it would not be possible, as there was no button to press to initiate borrowing. Instead, borrowing occurs automatically when a participant uses more shots than are available in their budget for a given level. We have added a sentence to the introduction to clarify this point: “Borrowing was only possible once participants exhausted their budget on a given level.” (p. 5). Thus, our original points about “poor” participants being presented the opportunity to borrow more frequently than “rich” participants (but not taking this opportunity as often) stand. We furthermore changed the description of borrowing within Table 1 to make this point clearer (“In the borrow condition, participants automatically “borrow” additional shots when they exceed their allotted shots for a given level. Borrowing only occurs after their budget is depleted, with a 100% interest rate applied: for each extra shot used, two shots are

deducted from the overall budget. This process is automatic, with no option for participants to initiate borrowing manually", p. 7).

Much of the focus here is around the original paper's interpretation of the allocation of attention. The original authors proposed to define the extent of participants' focus by measuring how long participants spent aiming each shot in the game. The present Table 2 contrasts the original observation, "Amongst participants who could not borrow, 'poor' participants spent significantly more time aiming the first shot of each level," with the authors' current "additional analyses," namely, "Although these 'poor' participants who could not borrow spent longer aiming their first shot of each level compared to the 'rich', they did not earn significantly more points on this shot compared to the 'rich'." The "additional analyses" are that "the longer aiming time of 'poor' participants did not actually translate into better outcomes for those shots." This, the present authors argue, "casts doubt on whether 'poor' participants' longer aiming time reflected their greater focus on the task." But why is that? One can think of many tasks, both easy and hard, where greater focus would not lead to better performance. In fact, it would seem highly ill-advised to argue that one's relative lack of success at a task shows a lack of focus.

R3A4: We agree of course with the reviewer that, generally speaking, greater focus on a task may not always lead to better performance on that task (indeed, in our Discussion, we write: "Of course, 'poor' participants could in principle have taken longer than 'rich' participants to aim their shots due to greater focus on the task, but absent a connection to better shooting performance, this interpretation is only one of many. It is equally plausible, given the data, that 'poor' participants were more inattentive or hesitant than 'rich' participants.", p. 15). However, as we note in our discussion, the original authors inferred greater focus in part from the behavioral outcomes of higher points-per-shot, in writing that "this engagement had some benefits for the poor." (quoted on p. 15). The inference of greater focus from connecting longer aiming times to better shooting performance also appears in Mullainathan and Shafir's (two of the original authors on the Shah et al paper) popular book on Scarcity, which we also quote in our paper: "...the poor did better: they were more accurate with their shots. This was not because of some magical improvement in visual acuity. The poor took more time on each shot. […] They aimed more carefully. They had fewer shots, so they were more judicious. The rich, on the other hand, just let the blueberries fly"

Our re-analysis shows very clearly that the poor did not have a higher average points-per-shot because they aimed better, but instead because they were restricted to taking the first three shots on each level, which were more profitable for all participants, since there were more targets to hit (moreover, as we show, average points-per-shot were not different between the poor and the rich when looking at only those first three shots). We do not claim in our paper that we can definitively rule out the possibility that the "poor" participants were more focused. However, given that their longer aiming times did not translate into better performance, it becomes more ambiguous what exactly is driving these longer aiming times. Being slower at a task does not necessarily equate to being more focused or engaged. What we can demonstrate is that, at least in this experiment, the supposed greater engagement did not result in any benefits for the poor, contrary to the original authors' claims.

The paper describes a “conceptual replication experiment,” involving the hypothetical purchase of insurance, where no significant difference in the tendency to purchase insurance is observed between “rich” and “poor” participants, except that “poor” participants were less likely to purchase negative expected value insurance. While the authors propose this as somehow providing additional evidence against the hypothesis that scarcity leads to attentional neglect of information, that interpretation remains entirely unclear. In fact, part of the argument by the original researchers is that scarcity often leads to greater attention to relevant information, which, to the extent there was any difference observed, it’s what was observed here.

R3A5: As we quote in our manuscript (p. 17), Shah and colleagues suggest in their original paper that "If scarcity creates a focus on pressing expenses today, then attention will go to a loan’s benefits but not its costs." By using insurance instead of borrowing, we retain the element of intertemporality: while the costs of insurance are incurred in the present, the benefits are realized in the future. Therefore, based on the original authors’ theory that scarcity causes individuals to focus on immediate needs while neglecting future consequences, the logical prediction would be that poor participants should be less likely to purchase insurance.

However, we agree more generally that the concept of tunneling proposed by the original authors is somewhat underspecified, as it is unclear when something should fall within versus outside the "tunnel."

Interestingly, in their discussion of tunneling, a concept closely related to the aforementioned comments on focusing, the authors argue that, “Absent a clear definition of what should fall within vs. outside the tunnel, it is impossible to falsify the theory, because it becomes possible, post-hoc, to attribute any process or decision that is enhanced among the poor to attentional focus, and to attribute any process/decision that is impaired to attentional neglect.” This sounds right. And in light of this, one wonders about the authors’ attempt to falsify the interpretations proposed in the study they are critiquing. The notion that participants’ focus is better gauged by successful shots rather than time spent is precisely the sort of concept lacking a clear definition that the authors so wisely advise against.

R3A6: In our manuscript, our goal is to evaluate the strength of evidence for the claims put forward by Shah et al. (2012, 2019). The original authors’ idea that "engagement had some benefits for the poor" (quoted on p. 15) is not something we introduced; it comes directly from the original publication and was a key basis for why the original authors inferred the presence of heightened attentional focus among “poor” participants. As noted above (R3A4), heightened focus is not the only explanation for longer reaction times, which we suspect is why it was important for the original authors to connect the longer reaction times of the poor to better shooting performance when inferring their heightened attentional focus.

The authors conclude that, “the better performance of the 'poor' in the original experiment did not stem from their greater focus on the task, as proposed by the original authors.” Better than what? The only times the word “better” appears in the original study (apart from other studies concerning previews of information) is in the observation that “poor participants fared

better when they could not borrow than when they could.” While, of course, it could be true that the observed effect does not stem from a greater focus, this reviewer sees no evidence supporting this conclusion.

R3A7: Thank you for pointing this out. We have revised the sentence for clarity. It now reads: “In sum, our additional analyses suggest that the better performance of the 'poor' compared to the 'rich' in the original experiment did not stem from their greater focus on the task, as proposed by the original authors.” (p. 10).

The authors are right to point out that “a better understanding of the cognitive and behavioral consequences of experiencing scarcity is important for informing policy recommendations about how to combat poverty around the globe.” In that spirit, the authors would do well to present research that enriched our data and our understanding – the present critical examination sheds little light on the issues of interest.

R3A8: We thank Reviewer 3 again for these comments, and hope that our responses have helped in clarifying the original authors’ claims, which our paper was directed at.

Point-by-point response for revisions of our manuscript “How does scarcity impact attention? A critical examination of the tunneling hypothesis”

Please find our point-by-point responses in bold below. We have not changed or deleted any part of the original reviewer letter.

REVIEWER COMMENTS

Reviewer #2 (Remarks to the Author):

I am happy that the editor decided to take a second look at this paper, as I was supportive of it the first time around and disappointed to see that it did not progress further at Nature Communications.

Having now read the other reviews, I can see why the authors have resubmitted it for additional consideration and think they are fully justified in doing so.

The first review is focused on limitations of the ability of the experimental paradigm to shed light on decision-making in response to real-world financial scarcity and thus any potential psychological mechanisms at play therein. The first limitation is one of ecological validity, and the second questions the ability or relatively small sample sizes used in random assignment to rule out the role of individual differences that may be shaping game behavior. These criticisms are important and ones that I would also make of much experimental research in the field, including my own.

However, I concur with the authors that they are not pertinent to the evaluation of the submitted manuscript, as the manuscript itself does not attempt to use such experiments for the above purpose. Rather, it aims to take a critical look at two key claims from a seminal paper in this field which was published in possibly the leading journal in all of the sciences. If such a scientific paper published in a highly respected, pan-disciplinary journal after rounds of rigorous peer review is found to be flawed in the claims it makes, it is essential that the scholarly community is informed of this in another major outlet, so that the record can be corrected. The need for correction is even more urgent given that this original paper has been cited nearly 2,000 times (according to Google Scholar) since its original publication, and is having an impact in the applied fields of international development (see, for example, the World Development Report 2015: https://www.worldbank.org/content/dam/Worldbank/Publications/WDR/WDR 2015/WDR-2015-Full-Report.pdf) and domestic poverty alleviation programs (see, for example, the discussion of this research program in https://s3.amazonaws.com/empath-website/pdf/Research-UsingBrainScienceDesignPathwaysPoverty-0114.pdf).

I am thus satisfied with the authors' response to Reviewer 1 concerning the empirical limitations of the original work. I am also happy with other responses to R1 and the clarifying text now added to the manuscript concerning participant dropout, exclusions, and deviations from the preregistration. The addition of power curves provides further insight into the power of the study given a range of SESOI. Relatedly, the explanation of how equivalence testing works in response to my own (naïve) comment on this in my review, as given by the authors in the response memo and in the revised manuscript, is satisfactory.

All in all, I agree with the authors that changes made in response to the detailed and thorough first review have improved the manuscript.

I'm also happy with how the other comment in my review was addressed, in terms of the short elaboration of the importance of these findings for the study of decision-making in poverty.

Finally, I do not think that the issues raised by the third reviewer challenge the credibility or importance of the current work, though they are useful as they have prompted the authors to add clarifying text that improves the manuscript. They key claim of the original paper by Shah et al. is indeed about the psychological mechanisms through which being 'rich' or 'poor' leads to particular decision-making patterns, not about the structural constraints imposed by the game. Yet the current manuscript convincingly demonstrates that for two of the key claims, it is the structural constraints that gave the appearance of a psychological effect where there is not in fact sound evidence for one. It is important for the scientific record, for policy applications, and indeed to correct how research in this area is taught in graduate courses, to know (1) that the increased time-per-shot exhibited by 'poor' participants is not a case of the kind of increased focus that improves performance, and (2) that the greater borrowing by 'poor' (versus 'rich') participants is not a case of being more likely (than the 'rich') to borrow where there is an opportunity to borrow. I am satisfied with the authors' response to R3 on these points and the clarifications made to the main text as a result.

Just one typo I noticed in the latest version on line 579: 'distribution of participant' is missing 's' et the end.

In summary, I continue to be strongly supportive of the publication of this work in Nature Communications, and do not think there are persistent issues that could justifiably prevent this.

Jennifer Sheehy-Skeffington.

A2.1: We thank the reviewer for her kind words and careful reading of our manuscript. We have corrected the typo she has pointed out, and have included a citation she suggested to highlight the policy implications of the original paper in our introduction (see p. 3 of the Manuscript). We sincerely appreciate her thoughtful and in-depth engagement with our work.

Reviewer #3 (Remarks to the Author):

As before, the paper presents re-analyses and re-interpretations of a study in Shah et al.'s 2012 paper, entitled "Some consequences of having too little." The original analyses are successfully replicated and the authors then present "additional analyses" to argue against some of the interpretations offered by the original authors.

The paper presents several prongs of critique: A different definition of attention, an analysis comparing the borrowing behaviors of the poor versus the rich who are out of budget, a new "insurance" intervention, and a structural analysis of the borrowing experiences of the 'rich' and 'poor.'

First, the authors propose a different definition of attention and find that, using this new definition, attention does not explain the findings. Their new definition requires of attention to lead to improved performance. The additional analyses showing that the longer aiming time of 'poor' participants did not actually translate into better outcomes, it is argued, casts doubt on whether 'poor' participants' longer aiming time reflected a greater focus on the task. This is an argument with no support. As pointed out before, equating attention with better outcomes is highly problematic. It is certainly not any more plausible than the original interpretation of attention. Among other things, Shah et al. observe, "The amount of borrowing by the poor was significantly correlated with measures of engagement. On rounds where poor participants borrowed, the average amount of time spent aiming each shot in their paycheck correlated positively with how many shots they subsequently borrowed."

A3.1: Thank you for sharing these critiques. We begin with the reviewer's last observation, of the correlation between amount of borrowing by the poor and average amount of time spent aiming each shot. As the reviewer suggests, this correlation has been used to support the idea that the increased aiming time reflects greater focus and thereby enhances performance on shooting, to the detriment of attention toward things "outside of the tunnel", leading to increased borrowing. Yet as we note in our manuscript, this correlation appears only within a narrowly defined and arbitrary subset of trials—specifically, those in which 'poor' participants chose to borrow. We do not observe a correlation between aiming time and borrowing when examining all trials, all borrowing trials among 'poor' participants, or trials involving both 'poor' and 'rich' participants who borrowed (see Table 2, Point 3.1, p. 9 in the Manuscript). The original authors do not explain why they focused on this specific subset, and importantly, this correlation did not replicate in their larger follow-up study (Shah et al., 2019), which we now note in a footnote (p. 13 of the Manuscript) and in Table 2, Point 3.1. As such, we do not believe this correlation reflects a meaningful or robust relationship.

Reviewer 3 further suggests that we have adopted a "different definition of attention" compared to the original authors. We are not sure which exact definitions the reviewer is referring to here. Shah et al. talk about the longer aiming times reflecting greater engagement, and that this "engagement had some benefits for the poor" (Shah et al., 2012, p. 683). Similarly, in their book, Mullainathan and Shafir (two of the original authors) write: "[...] the poor did better: they were more accurate with their shots. This was not because of some magical improvement in visual acuity. The poor took more

time on each shot. [...] They aimed more carefully. They had fewer shots, so they were more judicious. The rich, on the other hand, just let the blueberries fly." (Mullainathan and Shafir, 2013, p. 31). Our re-analysis simply shows that the poor did not do better due to better or more careful aiming, as noted on p. 10 of our Manuscript:

"Thus, the longer aiming time of 'poor' participants did not actually translate into better outcomes for those shots. This casts doubt on whether 'poor' participants' longer aiming time reflected their greater focus on the task, as originally claimed, or whether this could be explained by other factors, such as increased inattention or hesitation among 'poor' participants. Even if the longer aiming time of 'poor' participants reflected increased focus, it was not the kind of increased focus that led to better performance at shooting".

As this excerpt makes clear, we are not claiming that our re-analyses show that the 'poor' in this experiment were not more focused, however, given that the longer aiming times did not translate into better performance, it is more ambiguous what drove the longer aiming times. Being slower at a task does not necessarily mean being more focused. Our re-analyses show, at minimum, that in this experiment the longer aiming times did not have benefits for the poor, contrary to the claim of the original authors. As noted by Reviewer 2, correcting this claim is "...important for the scientific record, for policy applications, and indeed to correct how research in this area is taught in graduate courses".

Whereas the original Shah et al. paper presents five studies, the present critique of that research agenda looks at only one. Referring to Shah et al.'s paper, the preset authors write: "While the authors claim that Studies 3 to 5 provide additional supporting evidence, they acknowledge that these studies do not constitute direct evidence for the proposed cognitive mechanisms of tunneling." It's hard to know what to make of this claim. The word "tunneling" never occurs in the Shah et al. paper. And contrary to the authors' suggestion, Shah et al. write, for example: "Experiment 5 offers more direct support for the notion that scarcity creates attentional neglect."
(Reviewer 1 laments participants were all recruited through MTurk. In fact, Study 3 in the original involved participants in a lab.)

A3.2: First, Reviewer 3 correctly notes that the term "tunneling" was not introduced in Shah et al. (2012). However, the term was introduced shortly thereafter by some of the original authors (see Mullainathan & Shafir, 2013) and has since gained traction. The concept–and the original study by Shah and colleagues–is now widely recognized under this term (see, for example, de Bruijn & Antonides, 2022). For clarity, we also adopt this terminology in our paper.

Second, when stating that the original authors claim that Studies 3 to 5 only constitute additional supporting evidence, but do not constitute direct evidence for the proposed cognitive mechanism (i.e., a tunneling mindset leading to greater focus on scarce resources to the detriment of other potentially-beneficial information), we are referring to the following quote from Shah et al., 2012 in their discussion of Study 2: "An alternative account might be that the poor and rich approached these tasks

with the same mindset— playing each round until they were satisfied with their progress before moving on. By this account, the poor borrowed only because they were facing more severe constraints. But evidence from experiments 1 and 2 suggests that the poor and rich did not approach the tasks in the same way. The poor were more engaged" (p.684). Since Experiment 1 does not replicate (see Camerer et al., 2018; Shah, Mullainathan & Shafir, 2019), this leaves Experiment 2 as the main building block for the original paper's argument for a cognitive mechanism that explains both the positive and negative effects of scarcity, which is why we focus on it. We now further explain our reasoning in a footnote on p. 4 of our Manuscript.

Third, we note that the quote cited by Reviewer 3 suggests that Experiment 5 provides more direct support for the notion of attentional neglect, which represents only one component of the tunneling hypothesis (moreover, our additional re-analyses of Experiment 5, described below, contest the interpretation of attentional neglect). The main argument of our manuscript pertains to both aspects of the tunneling hypothesis—namely, that an increased focus on scarce resources leads to the neglect of other potentially beneficial future-related information . Accordingly, we (in agreement with the original authors) identified Study 2 as the central study supporting this argument, since it purports to show both increased focus and attentional neglect.

Finally, Reviewer 3's comment about our paper's focus on Study 2 motivated us to take another look at the available data for Studies 3 to 5, to see if we could reproduce the original analyses, as well as conduct analogues to our re-analyses of the Study 2 data. Not all details that would have been required for a full re-analysis were available online: specifically, we could not access the datasets of Shah et al. (2012) anymore. For Shah et al. (2019), the datasets that the original authors made available online were lacking appropriate labeling, and for Study 3, one variable seems to have been deleted from the dataset making it unsuitable for our analyses. However, we were able to reproduce the pattern observed in our re-analyses of Study 2 in Studies 4 and 5, as well.

That is, also in Studies 4 and 5 we can observe both a) the diminishing returns that created the interpretation of increased focus in Study 2, as well as b) the lower base rate frequency with which the 'rich' encountered the option to borrow from the future, which created the interpretation of attentional neglect in Study 2. In sum, our general critique of Study 2 - that the behavioral patterns that had been interpreted as reflecting increased focus and attentional neglect, can be better explained by structural features of the game participants played - also applies to Studies 4 and 5 of the paper. A detailed overview of these re-analyses can be found in the Supplemental Materials (p. **19-28*****), and it is alluded to in the main text in a footnote on p. 4.***

Next, the authors run a new "insurance" manipulation. And while the manipulation fails to observe any differences, the authors claim that "These findings provide additional evidence against the hypothesis that scarcity leads to attentional neglect of potentially beneficial information that does not pertain to present needs." That's a lot of evidence provided by a null result. There are obviously tons of other possible explanations for why this new

intervention did not work. The authors explain: “based on the original authors’ theory that scarcity causes individuals to focus on immediate needs while neglecting future consequences, the logical prediction would be that poor participants should be less likely to purchase insurance.” This is strikingly weak. First, even if those in scarcity were to neglect future consequences, what ensures that those not in scarcity would buy more of said insurance? Maybe it’s bad insurance? Maybe participants failed to notice it? In fact, most standard treatments of insurance argue that the poor should buy *more* of it than the rich. So if they didn’t, maybe that shows they failed to attend to it sufficiently, as per the original authors’ theory. This “conceptual replication” with null findings sheds little light on the issues. (Further confusing is the fact that we know in the real world the poor do buy less insurance than the rich. So no difference is the 'wrong' result, unless, of course, one assumed all of this has nothing to do with simulating poverty.)

A3.3: First, this is not an accurate characterization of our data from this study, as we did in fact observe significant differences between the ‘rich’ and the ‘poor’ in their purchasing of insurance in our conceptual replication study. Namely, the ‘poor’ were less likely to buy insurance with a negative expected value (i.e. “scam” insurance, or what Reviewer 3 calls “bad insurance”).

Second, we introduced insurances with varying expected value to address the point raised by the reviewer, namely that by simply observing a main effect of differences in buying insurances between the rich and poor, we cannot know where this main effect stems from. By varying the expected value, we can test whether participants buy (or avoid) insurance indiscriminately, or whether their choices depend on expected value—purchasing only those insurances with positive expected value. According to scarcity theory, people with fewer resources should focus more on immediate costs and therefore avoid buying insurance—even when it has a positive expected value. The ‘rich,’ by contrast, should be better able to consider future outcomes, choosing good insurance while rejecting exploitative, negative-value ones (as the reviewer suggests). In short, scarcity theory predicts that the rich should be more likely than the poor to buy only insurance with a positive expected value.

By contrast, we found in our conceptual replication study that the ‘rich’ and the ‘poor’ did not differ in their tendency to buy insurances with positive expected values. Critically, our analyses of this difference go beyond a simple null effect, but rather show with equivalence testing that there is actually no meaningful difference between the two groups, which allows us to interpret this null effect more meaningfully. As we argue in our revised manuscript, this indicates that –contrary to the original authors’ claim that scarcity leads to a <u>decreased</u> focus on decisions that pertain to the future–‘poor’ participants actually did process the information contained in the insurances, and if anything, showed an <u>increased</u> focus on the future compared to the ‘rich’ in this setup (given that they were significantly more likely to avoid purchasing scam insurances as noted above). We have clarified these points in our revised Manuscript on p. 14.

The present authors present an analysis comparing the behavior of ‘poor’ participants to that of ‘rich’ participants who are out of budget, and they find that there’s no difference. This is

entirely perplexing. There are no ‘poor’ or ‘rich’ participants in the original study – only participants with smaller or larger budgets. A ‘rich’ participant without a budget is a poor participant. In fact, the comparison the authors run is between poor participants and poor participants and they find no difference. (Imagine we assigned one group of participants to consume alcohol (the drinkers) and another group we gave only water (the non-drinkers). Our hypothesis was that drinkers would then drive less well. What we did next, for a truly “clean” comparison was to compare the non-drinkers to the “drinkers” who abstained from drinking. And we found there was no difference, thus disproving that alcohol hampers driving. This is roughly the logic of comparing the ‘poor’ to the ‘rich who have nothing.’)

In fact, because there are no rich and poor participants, since they are all randomly assigned, the only difference between ‘rich’ and ‘poor’ participants is the budget they have been allocated. The authors explanation, “our analyses suggest instead that the observed behavioral differences between ‘rich’ and ‘poor’ participants can be more parsimoniously explained by the game's structure,” is perplexing. Of course it’s the game’s structure. The only question is what in the structure yields the observed differences.

A3.4: First, we think it is important to clarify that our re-analysis did find behavioral differences between ‘poor’ participants and ‘rich’ participants who were out of budget. However, our results do not replicate the original authors’ findings, but instead point in the opposite direction. That is, when facing the decision of whether to borrow, ‘rich’ participants borrowed significantly more often, borrowing in 100% of trials, compared to ‘poor’ participants, who borrowed in only 69% of trials. This was despite the fact that the option to borrow was arguably more attractive for 'poor' participants, as they had significantly more targets left to clear, on average, after exhausting their budget (seeTable 2, point 2.1, p. 9).

That said, Reviewer 3 suggests here a novel way of conceptualizing the data: one could argue that with every shot used on the level, participants get more and more poor (though the inequity between poor and rich resets on each new level). This would mean that the binary comparison of ‘poor’ vs. ‘rich’ cannot be employed, but rather that scarcity would have to be conceptualized as a continuous variable, with people in different conditions starting at different starting budgets. This is an interesting perspective, but one that has neither been raised or addressed by the original authors, nor in any of the replication studies. One thing that needs to be kept in mind if one were to employ this approach is that when people use their shots there are also fewer and fewer targets to be cleared on the level. So participants’ increasing scarcity would have to be computed in relation to the targets remaining, which would make analyses highly complex. Crucially, the original authors also conceptualized their experimental manipulation as binary, meaning that they classified participants as ‘rich’ or ‘poor’ regardless of how many shots they had already used. Given this complexity, and since the aim of our re-analysis is to speak to the original results, we continue to follow the original conception of the manipulation as introducing a binary distinction between ’poor’ and ‘rich’ participants.

The flavor of the present paper is one of frustration. The authors are frustrated by the original work and look for ways to litigate it. But the critique come off as contrived. The

authors write: “We show that the behavioral effects of overborrowing cannot be attributed to attentional neglect, but were instead due to differences in base rate frequencies with which the ‘poor’ and the ‘rich’ faced the option to borrow.” Shah et al. (2019; the paper the present authors refer to and re-analyze) write: “We find weaker evidence for the hypothesis that scarcity-induced focus leads to attentional neglect. We fully replicated the experiments wherein greater scarcity led to more high-interest borrowing.” It’s very hard to tell the difference between those two claims.

The original Shah et al. study clearly is limited in scope, highly artificial, and requires further understanding. And its applicability to actual contexts of poverty certainly deserves further study. Unfortunately, beyond the frustration it expresses, the present paper does not shed light on the issues.

A3.5: We believe the critical distinction between these two claims reiterated by Reviewer 3 here lies in the strength of the evidence. While Shah et al. (2012) suggest that there is strong evidence for scarcity-induced focus (i.e., tunneling), and Shah et al. (2019) suggest that there is weak evidence for it, <u>we argue that these studies provide no evidence for it.</u> As we elaborate in our manuscript, we also find no empirical support for tunneling in the broader literature.

Finally, we would like to clarify that the current project was not born out of a motivation to litigate the original findings or to find ways to challenge them. On the contrary, we re-analyzed the dataset because we were genuinely impressed by the original work and hoped to build on it in our own research. However, we are convinced that our re-analyses demonstrate that the data presented in Shah et al. (2012, 2019) do not support the conclusions drawn—both by the original authors and by the broader research community. Given the significant influence this work has had, both within academia and in policy discussions, we believe it is essential to revisit and clarify these findings (a sentiment also echoed by the other reviewers). This process of critical engagement and reassessment is a core part of how science progresses. We sincerely hope that our critique will not be seen as a personal attack, but rather as a constructive contribution to the ongoing scientific dialogue.

Reviewer #4 (Remarks to the Author):

First of all, I think this is a great initiative to re-examine the seminal work by Shah et al. (2012). This paper received a lot of attention and opened an intriguing and important perspective on poverty with a huge impact on subsequent studies in particular and the literature on poverty in general. However, it also sparked a lot of skepticism (for good reasons), and the current paper is an attempt to reassess a core component of the theory by critically analyzing the empirical evidence and providing new evidence.

In my view, scarcity theory can be criticized on at least two fundamental grounds. First, while the groundbreaking studies – along with some follow-up work by different authors – have undoubtedly shaped the discourse, they are largely based on small samples and often lack research transparency. While such limitations were more widely accepted in the 2010s, the field has since evolved, and we must now push for greater transparency, replicability, and

open science initiatives. Scientific progress depends on rigor, and if a theory is built on limited empirical evidence or flawed conclusions, it is essential to reassess its validity with stronger methodological standards.
Second, the core concepts of scarcity theory are only loosely defined. While this flexibility has allowed for broad applicability and adaptability – sometimes serving as a defense against criticism – it also introduces significant challenges. When concepts are ambiguous, it is difficult to pin down precise tests and measurements, leading to inconsistencies in findings and difficulties in theory advancement. This lack of conceptual clarity may explain why accumulating evidence increasingly challenges some of the theory's foundational claims, such as the bandwidth tax. The present paper is a compelling illustration of this issue, and the discussion provides valuable insights into how the field can move forward.

In this regard, I strongly agree with Reviewer 2 that for scarcity theory to advance meaningfully, it requires a firmer theoretical foundation. Strengthening conceptual clarity will enhance both its explanatory power and empirical applicability, ensuring that future studies can provide more robust and conclusive evidence either in support of or against its key propositions. Given its critical engagement with these issues, the current paper is highly relevant, well-argued, and deserves recognition.

A4.1: We thank Reviewer 4 for the thoughtful and encouraging feedback and for reading both our manuscript as well as the original paper so carefully. We fully agree that conceptual clarity and methodological rigor are essential for the theory's progress, and our paper aims to contribute on both fronts.

However, I also see room for improvement. First, the sample size of the conceptual replication appears too small, especially considering that MTurk is known for its lower data quality. I also could not find information on quality controls, such as attention checks, etc. Although I see the reasons for using the same platform, I would have chosen a high-quality platform given the budget constraints. In the end, there are concerns about the reliability of the findings that outweigh the benefits of being as close as possible to the original study.

A4.2: First of all, we want to state clearly that we would be happy to collect more data once we understand what the additional data would be needed for. This said, let us respond to the issues raised by the reviewer's comments point-by-point:

- Sample size: We believe that our hypothesis and equivalence tests indicate that our current sample size for our conceptual replication study was already sufficient to detect the effects of interest (see Supplemental Materials, p. 11-16, and a new footnote in the main text on p. 15).

First, we were able to replicate the main findings of our re-analyses of the two datasets of Shah et al. (2012, 2019) in this new study, showing that, while the 'poor' spent more time aiming their shots and earned more points-per-shot across the game, this was not because of better shooting performance, but instead because later shots on each level were significantly less profitable for all participants, and the 'poor' were restricted to employing significantly fewer shots on each level. Since this new study replicates results that were already observed in two other datasets (one of them with

a highly powered study of N = 974), it is highly unlikely that we are observing false positives here.

Second, we further replicated the equivalence tests on the performance on shots 1 to 3, showing statistical equivalence in performance between the 'poor' and the 'rich'. We discuss power curves for hypothetical sets of effect sizes and sample sizes on p. 7-8 of the Supplemental Materials, which reveal that we were sufficiently powered to detect the Smallest Effect Size of Interest (SESOI) as defined in the manuscript.

The only equivalence test that suggested potential underpowering was the comparison between 'rich' and 'poor' participants in purchasing any type of insurance, irrespective of its expected value. However, this analysis conflates insurance with positive and negative expected values. The more central, theory-driven test involves a comparison between the 'rich' and the 'poor' in the purchasing of positive expected value (EV) insurance, as we noted in our pre-registration: "...we predict that the scarcity mindset will lead participants in scarcity-conditions to be less likely to purchase insurances than participants not under scarcity (H3). Specifically, we expect an interaction between condition (scarce vs. non-scarce) and the EV of the insurance, such that the difference in the likelihood of purchasing insurances is higher for insurances with higher EVs (H3a)." We have now clarified this prediction from scarcity theory on p. 14 of the revised Manuscript, and highlight that our analyses revealed equivalence between the two groups on this key contrast:

"We note that, just as in the case of the borrowing option presented in the original study, the insurance purchase option in our conceptual replication study had an intertemporal component: While participants had to pay the costs of purchasing insurance by sacrificing shots on their current level, the gains would only be realized in the future. Recall that scarcity theory claims that scarcity leads individuals to focus on immediate, pressing needs, while neglecting potential effects for their future well-being. Thus, by this account, the 'poor' should generally not purchase insurance, reflecting their attentional neglect of future outcomes, which means that they could miss out on insurance with positive expected value that could help them in the future. The 'rich', however, should selectively purchase only insurance with positive expected value, as they should recognize insurances with negative expected value as exploitative. As such, scarcity theory predicts that there should be a difference between the 'poor' and the 'rich' particularly in their purchasing of insurances with a positive expected value.Thus, according to the theory that scarcity makes individuals focus on current, pressing needs, while neglecting negative effects on future well-being., 'poor' participants should have been less likely than 'rich' participants to buy insurance.

Contrary to this hypothesis, we did not find a significant difference in the likelihood to purchase insurance with a positive expected value between the 'poor' (4.74 ± 2.84) and 'rich' participants (4.92 ± 2.92) in our study , F(1, 172) = 0.171.08, p = .682299, $\eta^2(1)$ = .0016. Indeed, equivalence testing revealed that the likelihood of purchasing insurance with a positive expected value was statistically equivalent between the 'rich' and the 'poor', t(169.56) = -2.052, p = 0.021, given our SESOI of d = .374 (see above). Moreover, comparing purchasing rates of insurances with negative expected value revealed that, iIf anything, 'poor' participants were more discerning in their

decision making: they were significantly less likely than 'rich' participants to purchase 'scam' insurances in which costs were higher than expected returns, $F(1, 172) = 8.24$, $p = .005$, $\eta^2(1) = .046$, while there was no significant difference in the likelihood to purchase 'reasonable' insurances with a positive expected value, $F(1, 172) = .17$, $p = .682$, $\eta^2(1) = .001$. Equivalence testing revealed that the likelihood to buy 'reasonable' insurances was statistically equivalent between the 'rich' and the 'poor', $t(169.56) = -2.052$, $p = 0.021$ (for more information on our conceptual replication study, see Supplemental Materials). These findings provide additional evidence against the hypothesis that scarcity leads to attentional neglect of potentially beneficial information that does not pertain to present needs."

In sum, if we were to conduct an additional study, our hypothesis of equivalence between the 'rich' and 'poor' would target the purchasing of insurance with positive expected value specifically, and power analyses would be benchmarked accordingly. Yet as already noted, our pre-registration focused on this specific contrast, and equivalence testing on this contrast indicates that our current sample already provides adequate power since the equivalence test was significant.

- MTurk: Reviewer 4 also raised questions about data quality on MTurk. A key reason we used the same platform (MTurk) as Shah et al. (2012; 2019) was to stay as close as possible to the original study design and participant population. We regard this as crucial, since the goal of our conceptual replication was to test whether the original empirical claims hold to our re-analytical approach. In other words, the key question underlying our work concerns the credibility of Shah and colleagues' original evidence, which has had significant scientific and policy impact. If we were to use a different platform, like Prolific, observed differences from the original studies could be attributed to unknown differences in the participant populations.

- Quality controls and attention checks: With regards to the data quality concerns, we failed to mention in our previous submission that we had already implemented several such quality control measures. The fact that these measures were already implemented adds to our confidence in the data collected from mTurk. We have now added these clarifications to the description of our conceptual replication study on p. 12 of the Supplemental Materials:

"To ensure reasonable data quality, we included bot-detection questions at the beginning of the study, as well as a comprehension check—consisting of three multiple-choice questions—administered before participants entered the Blueberry Game. Participants who failed these comprehension checks were excluded from further participation (for the exact wording, see our materials on OSF)."

Second, I find the strong focus on Study 2 unconvincing. If the existing data does not allow for a proper test of the attentional shift or tunneling hypothesis, this should be stated explicitly. However, the base rate argument should also apply to Studies 3 and 4. Study 5, which Shah et al. (2012) cite as providing "more direct support for the notion that scarcity creates attentional neglect," only reports performance results without addressing whether the "poor" participants actually had too little time to notice the previews. The authors should

engage with this study as well. After all, demonstrating that tunneling is not at work in Study 2 does not mean it is absent in the other studies. (The game structure argument, however, applies in all studies.)

A4.3: We agree with the reviewer and thank them for this suggestion, which we believe has considerably strengthened the paper. Per the reviewer's recommendations, we decided to include further re-analyses and discussions on Studies 3 to 5 in the Supplemental Materials. As outlined in response A3.2 in more detail, these new re-analyses provide additional support for our critique. That is, also in Studies 4 and 5 we can observe both a) the diminishing returns that created the interpretation of increased focus in Study 2, as well as b) the lower base rate frequency with which the 'rich' encountered the option to borrow from the future, which created the interpretation of attentional neglect in Study 2. We also note that the data needed to run these analyses for Study 3 were not available online, and we do not apply our re-analysis to Study 1 since it has already been found to not replicate (see Camerer et al., 2018; Shah, Mullainathan & Shafir, 2019).

A detailed overview of these new re-analyses can be found in the Supplemental Materials (p. 19-28), and are referenced in the main text in a footnote on p. 4. We believe that these new analyses further strengthen our suggestion that the observed behavioral effects that were interpreted by the original authors as reflecting tunneling–across different studies in the paper–can be better explained by structural aspects of the games that participants played.

Third, I have concerns about the reanalysis of the shots. The assumption that they are independent is problematic, as they likely depend on previous attempts, successes and failures, remaining targets, and other contextual factors. The analysis must account for these dependencies. A simple approach would be to average the first three rounds, but more sophisticated methods could be used for robustness.

A4.4: We agree with Reviewer 4 regarding the assumption about the independence of shots. In our view, it is precisely this assumption of independence that played an important role in the misleading interpretation of Shah et al. (2012)'s data. In fact, our re-analysis shows that the contextual factors mentioned by the reviewer significantly influence performance (as can be seen by the diminishing returns of shots over the course of each level, since with each additional shot, there are less targets to be cleared on a level). We also address the suggested analysis—examining only the first three shots of each level—by analyzing each shot individually. We find no differences, either when looking at the shots separately or collectively. Thus, even with this simple approach, we can conclude that there is no difference in performance on the first three shots of each level.

** Editorial note: Reviewer #4 also allowed us to share the following comment with regards to your rebuttal to Reviewer #1's concerns.

"They write, “Indeed, we did not include any other studies or additional tasks within this data collection […]. Participants only took part in the task outlined in the conceptual replication study, and all materials and procedures are fully disclosed.”

This author's statement is wrong (or their supplementary material, i.e., survey transcript, is wrong). The authors seemed to elicit time and risk preferences through two simple questions after the game. The authors need to clarify (i) why they did not follow the pre-analysis plan and (ii) why they provided a false response."

A4.5: We apologize for the confusion. We elicited time and risk preferences using the method developed by Falk et al. (2018), which includes 25 hypothetical choice items, with each participant answering five of these items per domain (not two questions, as the reviewer believed). While these risk and time preference measures were not part of the original Shah et al. (2012; 2019) study, we had included them in our pre-registration for our conceptual replication study (under the heading “Other”), and they can be found in the study protocol on OSF. Because we had thought of these tasks as part of our study, we did not think of these measures as “additional tasks within this data collection”, explaining why we responded to Reviewer 1 as we did. To prevent further misunderstanding, we have added a note to the methods section of our conceptual replication study in the Supplemental Materials on p. 12:

“After finishing their budget, participants were asked to complete measures of their time and risk preferences (based on Falk et al.; not reported here).”

Regarding deviations from the pre-registered analysis plan, although our analyses tested hypotheses that we pre-registered, we deviated from our planned analyses because we had misunderstood the structure of the original data files. Specifically, we had not anticipated that participants who used fewer points per level would contribute more observations to the results than those who used more shots per level. This endogeneity made the multilevel analytic approach that we had pre-registered inappropriate for these data. Instead, we adopted the analytical approach of the original authors (Shah et al., 2012), which also facilitates comparison of results. All deviations from the pre-registration are described on p. 17 of the Supplemental Materials.

Reviewer #5 (Remarks to the Author):

Thank you for the opportunity to review “How does scarcity impact attention? A critical examination of the tunneling hypothesis.” I was particularly pleased to see the authors adopt equivalence testing and offer the following comments aimed at strengthening that component for a future revision.

Clarifying the SESOI
Deciding on an equivalence bound is admittedly challenging, yet it is fundamental to interpreting phrases such as “no difference.” At present the bound is anchored to a statistical benchmark (33% power), which is a useful starting point, but readers cannot judge the practical meaning of your results without knowing why a deviation of that size—or

smaller—would be considered trivial in this context.

Discussing the bound in each interpretation
Whenever you conclude equivalence, consider reminding the reader to what the effect is equivalent (e.g., “within ±0.18 d, the smallest effect we have deemed practically important”). That transparency lets the audience evaluate the claim rather than the procedure.

Acceptable flexibility
It is perfectly reasonable to propose an exploratory SESOI—controversial or not—provided the rationale is explicit. You might present:
* a primary SESOI with your best theoretical or applied justification,
* a more liberal and a more conservative alternative (as you already explore in the sensitivity curves), and
* a brief discussion of how conclusions shift across them.
This lets future work refine the bound while still giving the present study a clear decision rule.

Relying on field expertise
Even though the task is artificial, you as domain experts are in the best position to offer an initial bound—whether grounded in prior literature, participant performance norms, or stakeholder costs.

By adding this targeted justification (and keeping the excellent sensitivity analyses), the manuscript’s equivalence claims will be both methodologically sound and far more impactful.

A5.1: We thank Reviewer 5 for their careful reading of our manuscript and their expert feedback on our equivalence and sensitivity analyses. We are glad to hear that the reviewer supports our overall approach.

As the reviewer rightly notes, many readers may not be familiar with equivalence testing, and we agree that greater clarity around how to interpret these results is essential. In response, we have revised the manuscript and supplemental materials to take the reviewer’s suggestion of adding explanations throughout the text. For example, we now include a footnote on p. 10, explaining and justifying our smallest effect size of interest:

“Following the guidelines of Lakens et al., the SESOI was set to the smallest effect size that the original study would have 33% power to detect. This SESOI was used for all equivalence tests reported here. See Supplemental Material for more details on our equivalence tests, and for sensitivity analyses showing similar conclusions with more conservative and liberal SESOI values.”

We further included brief interpretative statements alongside each equivalence test in the Supplemental Materials, explicitly reminding readers what it means for an effect to fall within our equivalence bounds. For example, on p. 6-7 of our Supplemental Materials, we now write:

“Similarly, performing equivalence tests on our conceptual replication study revealed statistical equivalence between the ‘rich’ and the ‘poor’ on the first shot, $t(170.6) = -1.851$, $p = .033$, as well as the second, $t(129.53) = 1.668$, $p = .049$, and third shot, $t(80.7) = 1.980$, $p = .026$. This means that also in our conceptual replication study the performance of the ‘rich’ and the ‘poor’ were statistically equivalent on all three shots, meaning that there was no difference between the two groups. These findings indicate that any differences between groups in the conceptual replication are smaller than our SESOI of $d = 0.374$ and therefore any differences observed can be considered to not be meaningful, given this criterion.”

We have also expanded the section on the SESOI in the Supplemental Materials (see pages 7-9) to justify our chosen threshold more clearly and now present and discuss several alternative SESOI. However, we do not adopt a SESOI derived from real-world effect sizes because it is difficult to define a practically significant effect size of interest in this context; the experimental setup is highly artificial, making it challenging to determine how these findings would translate to real-world outcomes (e.g., differences in overborrowing). Instead, we take the approach of examining whether and how our conclusions shift across more liberal versus more conservative bounds, as suggested by the reviewer. We believe these revisions improve the interpretability and impact of our findings and make our equivalence claims more accessible and meaningful to a broad scientific audience.

Reviewer #5 (Remarks on code availability):

Code was easy to follow and clear. No README file but do not think it was necessary.

** See Nature Portfolio’s author and referees' website at www.nature.com/authors for information about policies, services and author benefits.

This email has been sent through the Springer Nature Tracking System NY-610A-NPG&MTS

Confidentiality Statement:

This e-mail is confidential and subject to copyright. Any unauthorised use or disclosure of its contents is prohibited. If you have received this email in error please notify our Manuscript Tracking System Helpdesk team at http://platformsupport.nature.com .

Details of the confidentiality and pre-publicity policy may be found here http://www.nature.com/authors/policies/confidentiality.html

Privacy Policy | Update Profile

Point-by-point response to reviewers

Note that page numbers refer to the clean version of the manuscript/supplemental materials, and not the the version including tracked-changes

Reviewer #2 (Remarks to the Author):

I am satisfied that the authors have addressed all reviewer concerns, and think that the additional analyses run increase the contribution made even further. The clarifications and supplemental analyses also aid in reader interpretation.

R2A1: Thanks for your useful feedback and support throughout the peer review process.

Reviewer #3 (Remarks to the Author):

The authors argue that the Shah et al. paper they are critically examining has had a lot of influence, and that, therefore, corrections and a deeper understanding are essential. That sounds right. Unfortunately, this paper does not provide the kind of analyses that helps move the research forward. It starts with an alternative interpretation for which the authors agree they have no evidence, it then establishes a lack of correlation contrary to the original paper, which the original authors have already established in follow-up work, and it continues with two experiments that shed absolutely no light on the issues at hand.

To begin, the present authors replicate some of the central analyses, and then “contest the original claims.” They point out that although ‘poor’ participants aimed longer, which Shah et al. interpreted as a sign of greater focusing, the ‘poor’ did not do better. This paper is purportedly a look at “tunneling,” an attentional phenomenon. The original authors saw “time spent” as a proxy for attention. The present authors’ critique is that the extra time spent did not improve performance. The present authors have zero evidence contradicting the original notion of greater focus among the ‘poor’ (they write: “Note that we do not present any evidence against the tunneling hypothesis; rather, we highlight the lack of evidence supporting it”); their objection is that the ‘poor’ did not do better, and therefore, that they might not have focused more, as originally assumed.

A problem with the foregoing argument is that attention simply need not improve performance. This is a central tenet in Kahneman’s (1973) classic “Attention and Effort,” and it has been confirmed innumerable times since. The reason society devotes so much effort to improving the design of cockpits and roads and complicated documents is precisely because attention often does not bring about better performance. (And, conversely, in cases of expertise, improvement can be achieved with no greater attention.)

R3A1: We agree with the reviewer that, in general, greater attention or focus on a task does not necessarily lead to better performance, a point well established in the literature (e.g., Kahneman, 1973). Indeed, as we note in our Discussion, longer aiming times among 'poor' participants could in principle reflect greater focus, but absent a connection to improved performance, this interpretation is only one of several plausible explanations.

However, the original authors explicitly linked longer aiming times to better performance, interpreting them as evidence of greater engagement that "had some benefits for the poor" (Shah et al., 2012, p. 683). This interpretation is also articulated in Mullainathan and Shafir's book, which attributes the poor's higher points-per-shot to more careful aiming resulting from longer aiming times (see p. 5 of our manuscript). Our re-analysis directly addresses this claim. We show that the higher average points-per-shot among 'poor' participants was driven by their restriction to the first three shots on each level, which were more profitable for all participants due to a higher density of targets. When analyses are restricted to these shots, points-per-shot do not differ between 'poor' and 'rich' participants.

We do not claim that our analyses definitively rule out the possibility that 'poor' participants were more focused. Rather, our results show that their longer aiming times did not translate into better shooting performance, making the interpretation of longer aiming times as evidence of increased focus more ambiguous. At a minimum, our findings demonstrate that, in this experiment, longer aiming times did not confer performance benefits to the poor, contrary to the interpretation advanced by the original authors.

The other problem with this argument is substantial research, by several authors, in good journals. "We have presented evidence here," the authors continue, "that the behavioral differences Shah and colleagues observed between 'rich' and 'poor' participants in their study did not arise from attentional differences but instead from elements of the game's structure." There are an increasing number of studies showing clear attentional differences – sometimes focus, sometimes neglect -- between rich and poor (experimental participants and real people) that cannot be attributed to any game's structure.

Older research in marketing has tracked the food shopping strategies of low-income consumers and found that income significantly influenced price recall, suggesting lower-income shoppers were more attentive to and knowledgeable about prices (e.g., Wakefield & Inman,1993; Estelami & Lehmann, 2001). Steenhuis et al. (2011) find that "Low-income consumers were significantly more conscious of value and price than higher-income consumers." Spiller (2011) finds that the poor engage in more tradeoff thinking. Ellwood-Lowe, et al. (2022) find that caregivers who are prompted to reflect on financial scarcity speak less to their 3-year-olds in a play session, relative to a control group. Similarly, caregivers engage in fewer back-and-forth exchanges with their children at end of the month—when there's more financial hardship—than the rest of the month. Duquennois (2022) observes that lower SES students show evidence of depressed performance on monetary questions, compared to numerical questions that are virtually identical. Meuris & Leana (2018) report that worry about money made truck drivers less attentive on the job. Kaur et al. (2025) found that financially constrained workers, when they had less cash on hand, worked slower and less attentively, and made more mistakes. Speaking of

different mindsets of rich and poor, according to Huijsmans et al. (2019), “neuroimaging results suggest that a scarcity mindset affects neural mechanisms underlying goal-directed decision making.”

R3A2: We thank the reviewer for this thorough overview of research documenting attentional differences associated with poverty and financial scarcity. We agree that a substantial body of evidence suggests that socioeconomic conditions can affect attention in various ways, including leading to increased focus, neglect, or changes in trade-off thinking, both in experimental settings and in the field. As we also note in the manuscript (both in the introduction, p. 6, and the discussion, p. 15-16), our intention is not to claim that poverty has no effect on attention per se.

Rather, our point is more specific. The original Shah et al. (2012) paper invoked a tunneling account—namely, that increased focus on certain aspects of a task comes at the expense of attention to other relevant elements—to explain downstream behaviors such as overborrowing. Our re-analyses address this specific claim by showing that the behavioral differences observed in the original task can be fully accounted for by structural features of the game, without invoking attentional focus and neglect.

Thus, while we acknowledge and agree with the broader literature showing that scarcity and poverty can shape attention, our results speak to the interpretation of this particular experimental paradigm as evidence for a tunneling mechanism. Further, in our review of the literature, we found no direct empirical evidence of such a tunneling effect in other work, either (see our Discussion section).

One repeatedly puzzling claim is that the observed behavioral differences between ‘rich’ and ‘poor’ participants can be “more parsimoniously explained by the game's structure.” In fact, the game’s structure is the *only* difference leading to differential behaviors between the ‘poor’ and the ‘rich.’ The only serious, implicit question is whether that structure can explain the observed differences without any recourse to their effect on participants’ mindsets. It’s not clear whether that’s what the current authors’ believe -- everything we know from many other studies is that mindsets do tend to differ. But the conclusion that “we do not present any evidence against..; rather, we highlight the lack of evidence in support” is not terribly informative and strangely limited to this one study they are critiquing.

R3A3: We agree with the reviewer that the game’s structure is the sole experimental source of behavioral differences between “rich” and “poor” participants, and that the key question is whether these structural differences must be interpreted as operating through changes in participants’ mindsets. Our position is not that mindset differences cannot exist—indeed, substantial prior work suggests they often do—but rather that, in this specific task, the observed behavioral differences can be fully accounted for by structural features of the game without requiring an additional attentional or mindset-based explanation. Accordingly, our statement that we do not present evidence

against tunneling but instead highlight a lack of evidence in its support is intended to be narrow and specific to the experimental paradigms used in Shah et al. (2012, 2019). Note that we have added further analyses on Studies 4 and 5 of the original publication showing how the same logic applied to Study 2 also extends to these studies. Further, we review the literature for other studies claiming to find support for the tunneling effect, without finding any convincing evidence (see our Discussion section).

The “additional analyses” focusing only on those cases in which participants had the opportunity to borrow are truly puzzling. They compare participants who have no shots left with participants who have no shots left. Or to be more precise, they compare participants who have no shots left with participants who have no shots left but seconds ago had many (and apparently still keep on shooting..). Imagine a study of the habits of the rich and poor, where the rich are people who were born rich and now have nothing. If you did that, you would find, contrary to popular expectation, that both the rich and the poor sleep under bridges, go hungry, etc. This really makes no sense.

R3A4: With respect to the additional analyses focusing on borrowing opportunities, we agree that the task allows for more complex interpretations, as all participants become increasingly constrained over the course of a level and across the game. One could, as the reviewer seems to suggest, conceptualize scarcity as a continuous variable that evolves with each shot. However, this was neither how the original authors conceptualized or analyzed the manipulation, nor how it has been treated in subsequent replications. Importantly, any such continuous approach would also need to account for the simultaneous reduction in remaining targets, substantially complicating the analysis.

Because our aim is to evaluate and reinterpret the original findings on their own terms, we follow the original authors in treating scarcity as a binary manipulation (‘rich’ vs. ‘poor’), regardless of shots already expended. Our analyses are therefore designed to speak directly to the original claims, rather than to propose or test a fundamentally different conceptualization of the task.

Similarly, the insurance study, being entirely orthogonal to the issues at hand, really sheds no light. It observes no difference for insurance with positive expected value, and it observes the poor to be more discerning for scam insurance. Shah et al. really don’t seem to have anything precise to say, consistent or inconsistent with these patterns. The poor may be relatively neglectful of the positive insurance (which standard accounts say they should take more than the rich), or they might be attending to the scam insurance, which is highly financially threatening, and is where the poor are presumed to focus more. Like the original authors, the present authors have no clear predictions either, and both observed patterns, incidentally, contradict what happens between rich and poor in the real world.

R3A4: As we quote in our manuscript (p. 18), Shah and colleagues suggest in their original paper that "If scarcity creates a focus on pressing expenses today, then attention will go to a loan's benefits but not its costs." By using insurance instead of borrowing, we retain the element of intertemporality: while the costs of insurance are incurred in the present, the benefits are realized in the future. Therefore, based on the original authors' theory that scarcity causes individuals to focus on immediate needs while neglecting future consequences, the logical prediction would be that poor participants should be less likely to purchase insurance. We further want to note that we pre-registered our hypotheses in line with this logic.

So what, finally, does the paper provide? It argues, free of evidence, that a lack of improvement could be consistent with no greater focus. It establishes that there's a lack of significant correlation between aiming times and higher borrowing rates, an analysis that, as pointed out, Shah et al. reported failing to replicate several years ago. It provides a truly puzzling analysis where participants with a zero budget are called rich and found to behave like the poor, and it adds an "insurance study," which finds results that are inconsistent with what we observe in the real world, not predicted by the present authors, nor confirming or contradicting any clear prediction by the original authors. This is not a paper that will help a general readership better understand the issues at hand.

R3A5: We thank Reviewer 3 again for these comments and hope that our responses have helped to clarify the issues raised.

Reviewer #4 (Remarks to the Author):

I am happy with the authors' responses to my comments, and I continue to strongly support the paper. I have just a few notes on the responses.

1) MTurk, sample sizes, and data quality: My point was that MTurk data quality is poor; therefore, I would have preferred a larger sample, despite the power analysis. The added sanity checks (now more prominently described) are reassuring. So that's fine. The argument for using the same platform as the original is less convincing; it also applies to the current setup, i.e. observed differences could be attributed to unobserved effects over time.

R4A1: We agree that MTurk data quality can be a concern. However, a positive aspect of our results is that the effects replicate robustly both in a substantially larger sample and across a long time span: more than ten years have passed between the first (Shah et al., 2012) and the most recent (our conceptual replication) data collection. This temporal robustness reduces the likelihood that our findings are driven by idiosyncratic features of a single dataset or moment in time. We note, however, that replication on the same platform does not rule out the possibility that shared data-quality issues contribute to

similar results across studies. For this reason, our manuscript prominently describes the added data-quality and sanity checks, which help mitigate—though not fully eliminate—these concerns (see p. 22): "To ensure reasonable data quality, we included bot-detection questions at the beginning of the study, as well as a comprehension check—consisting of three multiple-choice questions—administered before participants entered the Blueberry Game. Participants who failed these comprehension checks were excluded from further participation (for the exact wording, see our materials on OSF)."

Importantly, the central goal of our paper is to test whether there is empirical support for the claims made by Shah et al. (2012). Our results suggest that this is not the case, and we believe that this conclusion can be appropriately drawn from our re-analyses of the original data, combined with our well-powered, carefully checked replication study conducted on MTurk. We have now included a limitations section in the Discussion of our manuscript (p. 21): "While our reanalysis and conceptual replication provide evidence that the behavioral effects reported by Shah et al.[1] cannot be attributed to the proposed attentional tunneling mechanism, several limitations should be noted. Both the original studies and our replication rely on highly artificial laboratory paradigms that may not capture the complexity of real-world poverty, limiting external validity—although such approaches are common given the ethical and practical constraints of manipulating poverty in the field. In addition, all studies used online samples recruited via MTurk, which raises general concerns about data quality and platform-specific influences. These considerations suggest caution regarding generalizability, even though they do not undermine our central conclusion that the original data do not support the proposed mechanism."

2) Re-analysis of Studies 3-5: I am glad that the authors now also re-analyzed the other studies and that the results align with my prior. Although I am a bit surprised the original study's data is no longer available online (nor obtainable from the authors?). Since Study 3 cannot be re-analyzed, the manuscript and supplemental material should not claim that "Studies 3 to 5" were re-analyzed or that the critique of Study 2 "also applies to Studies 3 through 5." Such claims can only be made for Studies 4 and 5.

R4A2: Thank you for pointing this out. We have revised the manuscript and supplemental materials to remove claims that "Studies 3 to 5" were re-analyzed or that the critique of Study 2 applies to Studies 3 through 5, and instead state explicitly that these points apply only to Studies 4 and 5, as Study 3 cannot be re-analyzed (for example, on p. 8).

3) Non-independence of shots: My point is that the second (and third) shots are not independent observations, and the analysis should in some way account for it. An easy fix would be to average the three shots within the poor and rich conditions and compare those averages and only then analyze shots independently (of course, one could do more sophisticated stuff). Other

than noted in the author's response, I do not see this analysis in the manuscript or supplemental material.

R4A3: Thank you for clarifying your point. We agree that this analysis provides a cleaner comparison. We have now added this analysis for all three datasets to the manuscript (see, for example, Table 2 in the manuscript; Table S1 in the Supplemental Materials; and p. 10 of the manuscript). We have also included the corresponding equivalence tests (see p. 7 of the Supplemental Materials). Across all datasets, the results show the same pattern as the analyses of individual shots, and thus the overall interpretation remains unchanged.

4) Additional measures, pre-registration: The authors clarify the issue about the additional measures. The pre-registration lists two additional hypotheses (H5 and H6), but they are not reported anywhere (unless I missed it). I think one should always follow through with the plan and report all pre-specified analyses somewhere. What ends up in the paper is a different story (only a few people can envision their complete paper before they have seen the data).

R4A4: We agree that it is good practice to follow through on all pre-specified analyses and to report them somewhere, regardless of whether they ultimately appear in the main text. We have therefore added a discussion of the pre-registered additional hypotheses (H4 and H5) to the Supplemental Materials (p. 13): "Hypotheses 4 and 5. In addition to the hypotheses reported in the manuscript, we preregistered two further hypotheses (H4 and H5) in the "Other" section of the preregistration. In H4, we predicted that time preferences and risk preferences would mediate the relationship between the scarcity manipulation and insurance purchasing. However, there are several reasons why testing this mediation is not warranted. First, we do not observe a main effect of the scarcity manipulation on insurance purchasing ($t(172) = 1.041$, $p = .150$). Second, we find no significant differences between participants in the 'poor' versus 'rich' conditions with respect to time preferences ($t(172) = 0.510$, $p = .306$) or risk preferences ($t(172) = -1.132$, $p = .130$).

With respect to H5, we employed two different scarcity manipulations: one involving income shocks and one without income shocks (see Materials on OSF for details). We predicted that scarcity induced via income shocks would produce stronger behavioral effects than scarcity manipulations based solely on high versus low income levels. Specifically, we hypothesized an interaction between the type of scarcity manipulation (income shock vs. no income shock) and condition (scarce vs. non-scarce) across our three main hypotheses (H1–H3). However, similar to H4, we do not observe differences attributable to the income shock manipulation in key behavioral outcomes (e.g., points per shot or time spent aiming; all $ps > .157$). Consequently, conducting mediation analyses is not warranted."

Reviewer #5 (Remarks to the Author):

Thank you to the authors for the careful revisions and the additional interpretive clarity. The expanded discussion of equivalence tests in both the main text and supplement substantially improves readability, especially for readers less familiar with the method. I also appreciate the addition of alternative SESOI and the sensitivity analyses—this makes the manuscript more transparent and flexible.

I do want to touch upon two points, which I believe should be addressed to make the equivalence tests effective:

1. Substantive justification of the SESOI.

I understand the challenge of defining a real-world benchmark in an artificial task, and your decision to rely on the 33% power rule is methodologically defensible. That said, I still encourage you to consider at least a provisional domain-based rationale (e.g., performance differences that would matter for decision-making, or even qualitative stakeholder perspectives). Even a tentative justification would help anchor the SESOI in substantive meaning rather than leaving it entirely at a statistical convention. Without this, the “so what?” of equivalence remains harder for readers to evaluate.

R5A1: We agree that providing context for the SESOI would be valuable and that relying solely on a statistical convention can make it harder for readers to evaluate the practical meaning of equivalence. After careful consideration and discussion with colleagues familiar with similar experimental paradigms, we have therefore added an interpretation of the SESOI within the context of our task. Specifically, we now relate the SESOI to performance differences in points-per-shot, thereby offering a concrete sense of what such a difference would imply within our experimental setting. While we acknowledge that any such interpretation remains necessarily provisional given the artificial nature of the task, we believe this addition helps anchor the SESOI in more substantive terms and clarifies its relevance for decision-making in the context of the experiment. We now added in the Supplemental Materials (p. 6): “When applied to the measure of points-per-shot, this effect size corresponds to a mean difference of approximately 0.271 points-per-shot, given the pooled standard deviation of .724 points-per-shot observed in Shah et al. (2012).”

2. Terminology of conclusions.

In a few places the manuscript equates equivalence with “no difference.” I would suggest instead framing this as “negligible difference” or "no meaningful difference". The distinction matters because the SESOI defines what counts as negligible; differences outside it could still be meaningful (and conversely, non-zero differences that one finds are trivial as long as they are within the bounds). This refinement would better communicate the logic of equivalence testing.

R5A2: Thank you for this suggestion. We have revised the manuscript to replace statements equating equivalence with “no difference” with language referring to “no meaningful difference,” and clarified that equivalence is defined relative to the SESOI.

Overall, the revisions have strengthened the paper, and addressing these two points would further align the manuscript with best practices in equivalence testing and improve the interpretability of your conclusions.

Thank you for a chance to read the manuscript and I wish the authors the best of luck in this interesting work!
